# The interplay between stack pressure, mechanical expansion and degradation pathways in lithium-ion batteries

**DOI:** 10.1038/s41560-026-02087-6

**Published:** 2026-06-29

**Authors:** Heng Wang, Rui Wang, Christopher A. O’Keefe, Erik Björklund, Daniela Proprentner, Joe C. Stallard, Hwee Jien Tan, Wesley M. Dose, Louis F. J. Piper, Robert S. Weatherup, Angkur J. D. Shaikeea, Clare P. Grey, Michael De Volder

**Affiliations:** 1 Department of Engineering, University of Cambridge, Cambridge, United Kingdom; 2 The Faraday Institution, Quad One, Harwell Science and Innovation Campus, Didcot, United Kingdom; 3 Yusuf Hamied Department of Chemistry, University of Cambridge, Cambridge, United Kingdom; 4 Department of Materials, University of Oxford, Oxford, United Kingdom; 5 Warwick Manufacturing Group, University of Warwick, Coventry, United Kingdom; 6 School of Chemistry, University of Sydney, Camperdown, New South Wales Australia

**Keywords:** Batteries, Mechanical engineering

## Abstract

While electrochemical degradation mechanisms in lithium-ion batteries are well studied, the influence of mechanical factors remains poorly understood. Here we introduce a high-precision stack-pressure control and dilatometry tool to apply a uniform and constant stack pressure on electrodes independent of electrode swelling. By increasing stack pressure fourfold over typical initial values, we double the lifetime of graphite ‖ LiNi_0.8_Mn_0.1_Co_0.1_O_2_ cells, an industrially relevant battery chemistry, without altering active materials or electrolytes. This suggests that many lithium-ion batteries operate under sub-optimal stack-pressure conditions, leading to curtailed lifetimes. We demonstrate that different degradation mechanisms emerge outside the optimal pressure window: low stack pressure accelerates cathode cracking, whereas high pressure promotes lithium plating. Our findings highlight coupled mechanical–electrochemical degradation mechanisms and identify stack-pressure optimization as a practical solution for increasing cycling stability.

## Abstract (web summary)

While electrochemical degradation in lithium-ion batteries is well studied, the role of stack pressure in long-term cycling remains unclear. Here the researchers use a high-precision pressure-controlled dilatometry tool to optimize stack pressure and understand how it prolongs battery lifetime.

## Main

The widespread use of lithium-ion batteries (LIBs) in applications ranging from consumer electronics to electric vehicles necessitates continuous enhancements in energy and power density, safety and longevity^1^. To improve sustainability, substantial efforts have focused on extending cycle life by elucidating the correlations between degradation and key factors, including active material chemistry, electrolyte composition, cycling protocols, operational environments and manufacturing conditions.

The importance of stack pressure is well established for solid-state and metal-anode batteries^2^, and also in standard LIBs for maintaining good particle contact^3^, reducing ionic resistivity^4–7^, preventing delamination^8^, limiting particle and excessive solid electrolyte interphase (SEI) cracking^5^ and expelling generated gases^6,9^. However, excessive pressure could reduce electrolyte infiltration^10^, limit active surface area^6^ and cause separator pore closure^8,11^. While there have been several studies focusing on short-term effects during the initial tens of cycles, the effect of stack pressure on long-term cycling stability remains underexplored in classic LIBs.

The fixed casing of coin cells makes them impractical for stack-pressure studies, particularly as electrode thickness changes with state-of-charge (SOC) and state-of-health (SOH)^8,12–15^. By contrast, the flexible packaging of pouch cells offers the opportunity to apply external pressure independently of electrode swelling. Currently, stack pressure in pouch cells is typically controlled by compressing the cells between plates using bolts and springs (Supplementary Note 1 and Supplementary Fig. 1). However, such pressure varies with the SOC and across the lifetime of the cell as the volume of the battery materials changes and materials creep (Supplementary Figs. 2 and 3). In the case of graphite (Gr) ‖ LiNi_0.8_Mn_0.1_Co_0.1_O_2_ (NMC811) cells, this pressure is the highest at the top of charge, which is when the cathode active material is in its mechanically weakest state^16,17^, which may be sub-optimal.

In this Article, we have developed a dilatometer with compliant pneumatic bellows that define the stack-pressure to investigate how this influences the long-term cycling stability of Gr ‖ single-crystal NMC811 cells (see details in Supplementary Table 1). We tested our cells at 3 bar (denoted as low pressure, LP) and compared the results with those obtained at 1.5 bar (extra low pressure, XLP), 6.5 bar (medium pressure, MP), 12.5 bar (optimal pressure, OP) and 37.5 bar (high pressure, HP). Remarkably, a twofold increase in cycling life was obtained by applying a constant pressure that was approximately four times higher than the standard initial pressure used in conventional coin (Supplementary Fig. 4) and prismatic cells^7,18^. By combining operando high-resolution dilatometry with post-mortem analyses, a counter-intuitive process was discovered. Low stack pressures accelerated cathode cracking, leading to an increase in transition metal (TM) dissolution and excessive SEI formation. At high pressure, on the other hand, degradation mechanisms linked to Li plating on the anode were observed. This study elucidates the interplay between mechanical and electrochemical degradation as a function of stack pressure and suggests strategies to extend battery lifetime.

## Combining stack-pressure control and dilatometry

**Fig. 1:**
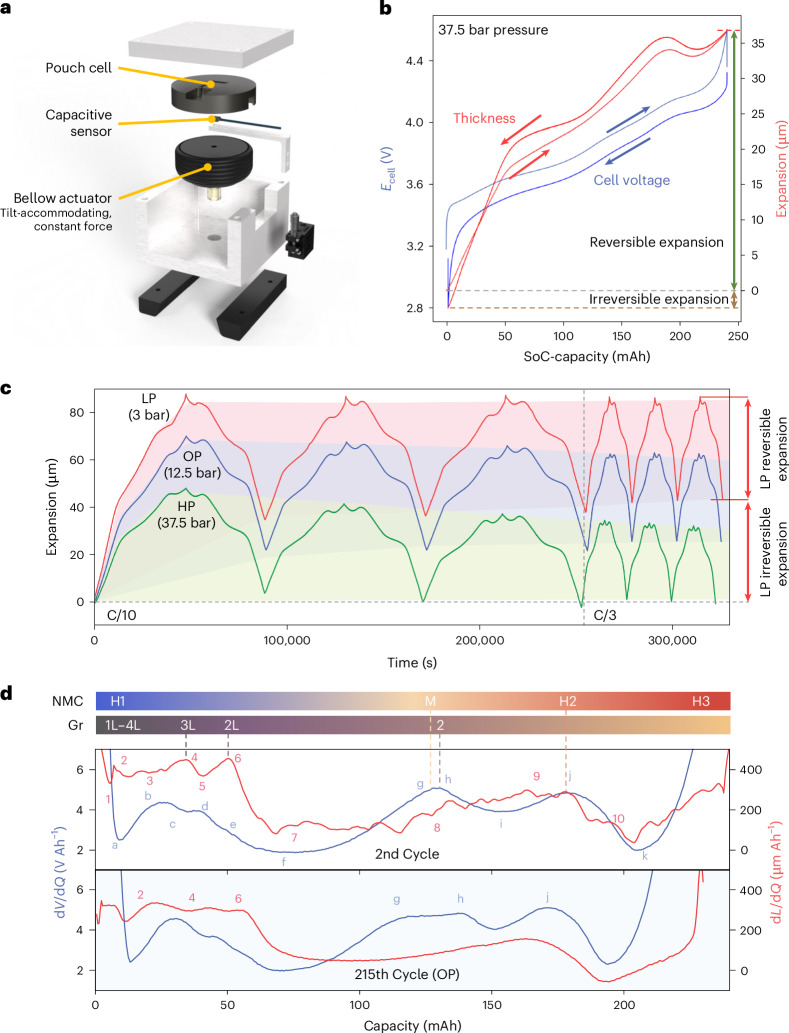
Operando dilatometry reveals pressure-dependent thickness evolution in lithium-ion pouch cells. **a**, Exploded view of the components of the custom-built dilatometer and stack-pressure control tool. **b**, Cell thickness expansion (red) and voltage (blue) plotted against state-of-charge capacity during the third C/10 formation cycle under the HP stack-pressure condition. The data show a 36.7 μm expansion during charging, followed by a 39.1 μm contraction during discharging, corresponding to a 2.4 μm irreversible contraction. **c**, Cell thickness expansion during the initial three C/10 slow formation cycles and subsequent three C/3 cycles under LP (red), OP (blue) and HP (green) conditions. The colour-shaded regions indicate the reversible expansion ranges for each pressure condition. **d**, Differential cell thickness expansion (d*L*/d*Q*) and differential voltage (d*V*/d*Q*) plotted against capacity during charging for a representative early cycle (cycle 2) and a cycle after ageing (cycle 215). From left to right, the upper colour scale shows the NMC structural evolution regions during delithiation, while the lower colour scale shows the graphite lithiation stages. The key features are indicated by labels 1–10 in d*L*/d*Q* and labels a–k in d*V*/d*Q*, identifying the corresponding features captured in the dilatometry and voltage responses. The H1–M–H2–H3 labels follow conventional notation from LiNiO_2_ (LNO) literature and are used here to describe analogous regions of structural evolution in NMC811, although these do not represent distinct phase transitions; instead a solid-solution behaviour is seen throughout and for this reason we refer to these regions as ‘H1’, ‘M’ and so on^19,20^. Source data

We have developed a dilatometer with compliant pneumatic bellows (Fig. 1a) using pressure control valves. This has the advantage of maintaining a constant stack pressure on pouch cells, independent of the SOC or cycling age (Supplementary Note 2 and Supplementary Figs. 3 and 5). A second advantage of bellow actuators is that they are compliant and self-align to the cell surface to eliminate stress concentrations that can arise from manufacturing imperfections, as illustrated in Supplementary Fig. 3. This design enables homogeneous pressure distribution and limits pressure fluctuations below 0.8% (Supplementary Fig. 6), which is about an order of magnitude lower than conventional bolted (~20%) or spring-loaded (~6%) in the setups we have characterized (Supplementary Fig. 2). Our setup also mitigates up to 20% initial relaxation-induced stack-pressure loss in spring-loaded or bolt-loaded systems, which can make accurate stack-pressure optimization challenging.

In addition to controlling stack pressure, the tool also measures the cell expansion and contraction using a high-accuracy capacitive sensor with a resolution of 0.38 nm and a drift of less than 100 nm per month (details in Supplementary Table 2). Additionally, the pouch cells have a large ‘bag’ (Supplementary Fig. 6c) to accommodate gases outside the area where the electrodes are located, thereby separating gas evolution processes from the applied stack pressure.

Figure 1b shows that our dilatometer is sufficiently accurate to distinguish between reversible and irreversible electrode expansion and to capture mechanical events such as the *c*-lattice collapse of the NMC cathode at high SOC (refs. ^4,12,19^). The reversible expansion stems from inherent particle expansion with SOC (refs. ^8,12^), while irreversible expansion is correlated to processes including particle reorganization, SEI layer formation, Li plating and particle cracking^13–15^.

In the first six cycles, we find that the stack pressure directly influences the thickness expansion profile (Fig. 1c). Higher stack pressures reduce irreversible expansion in the initial formation cycles, and result in a more ‘symmetrical’ reversible expansion and contraction profile between charge and discharge (Fig. 1c and Supplementary Fig. 7).

## Long-term cycling and differential voltage analysis

All cells were cycled in a standard LP57 electrolyte (1 M LiPF_6_ in ethylene carbonate/ethyl methyl carbonate, 3:7 v/v). This baseline was deliberately used to eliminate the effects of additives, which might complicate the interpretation of pressure-induced electrode degradation. For completeness, we compared the stability of a cell with and without 2 wt% vinylene carbonate (VC) additive added to the electrolyte (Supplementary Fig. 8).

The voltage window between 2.8 and 4.6 V was chosen because a nominal upper cutoff voltage (UCV) of 4.6 V corresponds to an effective UCV of ~4.3 V at C/3 (Supplementary Fig. 9) due to an overpotential of approximately 0.3 V at high SOC (ref. ^20^). Therefore, cycling to a UCV of 4.6 V ensures that all cathodes undergo the *c*-parameter collapse (also referred to as the H2–H3 transition, see Fig. 1d) that may influence the degradation behaviour (Supplementary Note 3).

**Fig. 2:**
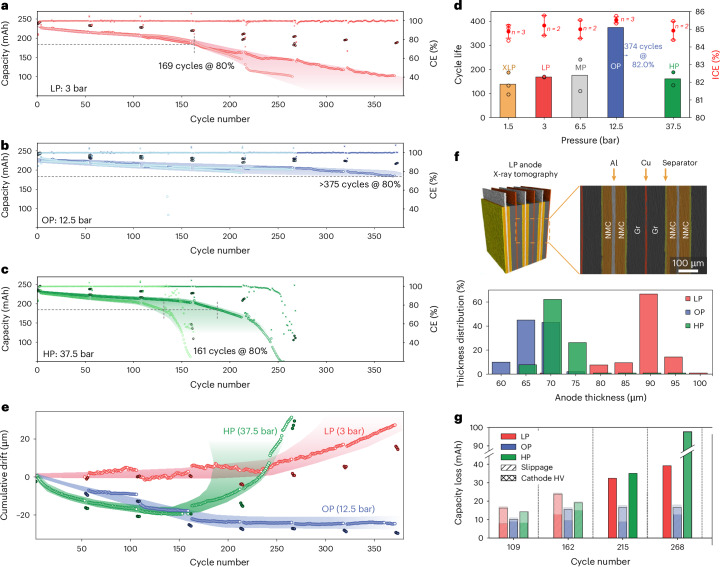
Stack pressure governs cycle life and irreversible expansion in pouch cells. **a–c**, Long-term cycling capacities and Coulombic efficiency under LP (**a**), OP (**b**) and HP (**c**) conditions. The cells were cycled at a C-rate of C/3 (70 mA) in a voltage window of 2.8–4.6 V, with slow C/10 (21 mA) cycles during formation and recovery, indicated by solid points. **d**, Cycle life and initial Coulombic efficiency (ICE) as a function of stack pressure in log scale. Individual data points are shown alongside mean values. The error bars for ICE represent the standard deviation (*n* indicated). **e**, Cumulative irreversible cell expansion under LP, OP and HP conditions from cycle 3 onwards. The solid points represent slow C/10 recovery cycles. **f**, XCT analysis of the thickness of the cycled anodes under LP (374 cycles), OP (374 cycles) and HP conditions (268 cycles). The anode thickness of the segmented volume of three-dimensional reconstructions was sampled using a randomly distributed sphere-fitting algorithm, with LP anodes showing the highest thickness on average. **g**, Differential voltage analysis of the C/10 cycles, highlighting the fitted contributions of slippage loss and cathode high-voltage (HV) capacity loss to the overall measured capacity loss under LP, OP and HP conditions. The hatched regions represent the fitted DVA contributions, while the difference between the hatched sections and the solid-colour bars indicates the residual difference between the fitted losses and the measured total capacity loss. Source data

In the first 100 cycles, a similar depth of (dis)charge is achieved in all stack-pressure conditions (Fig. 2a–c and Supplementary Fig. 10a,b). Specifically, the charge capacities are all within 1.1% and the discharge capacities within 2.7% difference in the first cycle (Supplementary Fig. 10c), indicating similar levels of lithium utilization during initial operation.

Figure 2a-c shows how the capacity degradation varies with stack pressure, with an average 80% capacity retention reached after 169 cycles for LP cells and 161 cycles for HP cells, both less than half of the cycle life of OP cells (>375 cycles). This is remarkable as OP cells operate at 12.5 bar, about 4 times higher pressure than we measured in standard coin cells (Supplementary Fig. 4) and above typical initial values reported for other cell formats^5–8,18,21^.

Figure 2d shows the cycling stability and initial Coulombic efficiency (ICE) as a function of pressure (1.5 to 37.5 bar), and again indicates that an optimum is reached around 12.5 bar (more data in Supplementary Figs. 10 and 11).

Figure 2e shows the cumulative irreversible expansion (change in thickness in the discharged state) of the cells. When applying pressures that are too high (HP, 37.5 bar), the cell compacts incrementally during the first ~150 cycles (probably due to a decreasing porosity), at which point it starts expanding irreversibly on each cycle (more data in Supplementary Fig. 12). It is interesting to note that this start in expansion coincides with the onset of accelerated battery degradation, suggesting a coupling between degradation and irreversible cell expansion. When insufficient pressure is applied (LP, 3 bar), the cell initially swells slightly every cycle, and after ~220 cycles the swelling rate increases, which again coincides with accelerated degradation rates. However, the shape of the irreversible expansion curves differs for LP and HP, suggesting distinct degradation mechanisms. In the case of our optimal pressure (OP, 12.5 bar), the cell slowly compacts during the first 200 cycles and then reaches a stable thickness.

To understand whether irreversible thickness expansion is caused by the anode, cathode, separator or some combination of these, X-ray computed micro-tomography (XCT) scans of cycled pouch cells were performed, with a false colour three-dimensional reconstruction provided in Fig. 2f. While the thickness of cathodes and separators remained similar within the accuracy of the tool (Supplementary Fig. 13), segmented tomography analysis showed that the average cycled anode thickness increased most for LP (89 µm), followed by HP (72 µm) and OP (67 µm). This ordering of anode–thickness growth was verified using a micrometre gauge (Supplementary Fig. 14).

To identify how capacity is lost in different stack-pressure scenarios, we first carried out differential voltage analysis (DVA), which allows for quantitative identification of the levels of capacity loss due to anode, cathode and Li-inventory loss by examining the evolution of the differential voltage (d*V/*d*Q*) curves over time (Supplementary Note 4 and Supplementary Fig. 15)^22–24^.

Figure 1d shows a characteristic d*V/*d*Q* curve as well as analogous curves tracking changes in thickness (d*L/*d*Q*) (more data in Supplementary Fig. 16). The latter allows better identification of electrochemical transitions in the anode (features labelled 1 to 6 in Fig. 1d) that are less visible in d*V/*d*Q* (see the full assignment of phases in Supplementary Table 3)^4^, illustrating the synergy between voltage and expansion data.

After the first 109 cycles, the loss of Li inventory (slippage) was similar for all stack pressures (Fig. 2g) and accounted for about half or more of the total capacity loss, as expected from the literature^22,23^. However, at this stage, both the LP and HP cells show a higher cathode capacity loss (note that for all conditions and cycle numbers fitted here, the NMC capacity losses are predominantly above 4.1 V, see Supplementary Fig. 15f).

At 162 cycles, the capacity loss due to slippage remains similar in OP cells, but increases in LP cells and increases even more severely in HP cells. We will demonstrate further below that HP cells show signs of Li plating, which is consistent with the observed slippage. The anode capacity in LP cells calculated from DVA appears to decrease after 162 cycles (Supplementary Fig. 15e), which may be linked to the increase in thickness discussed above (Fig. 2e) with isolated graphite becoming inactive, for which more evidence is provided later in the manuscript.

Beyond 200 cycles, DVA of LP and HP cells becomes unreliable due to the loss of pronounced features within the voltage curves. By contrast, the OP cells exhibit limited changes in slippage and high-voltage cathode capacity loss (Fig. 2g).

## Anode degradation mechanisms

To identify the actual anode degradation mechanisms, we combined optical microscopy, nuclear magnetic resonance (NMR) spectroscopy, synchrotron X-ray diffraction (sXRD), X-ray photoelectron spectroscopy (XPS), microwave plasma atomic emission spectroscopy (MP-AES) and cross-sectional time-of-flight secondary ion mass spectrometry (ToF-SIMS) analyses.

**Fig. 3:**
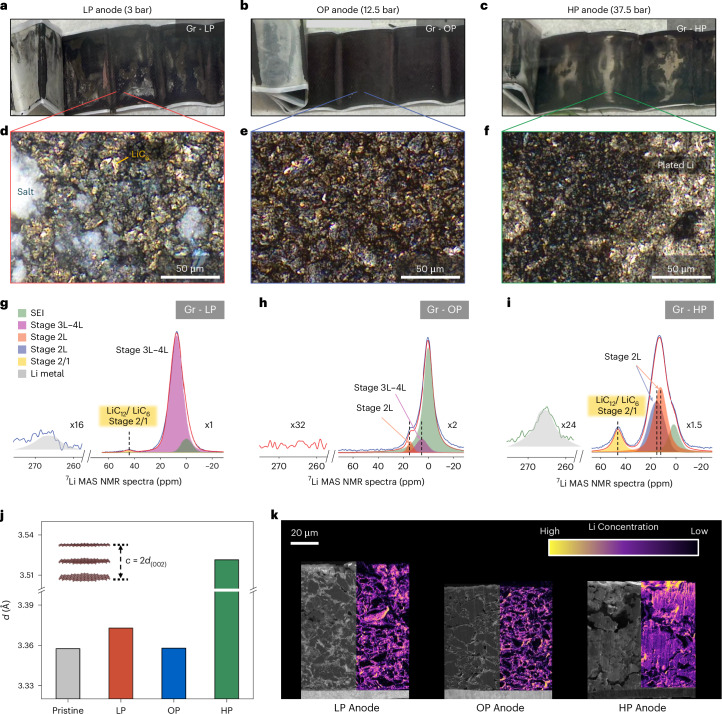
Pressure dependent lithium heterogeneity and structural evolution in cycled graphite anodes. **a**–**i**, Post-mortem analyses, including photographs (**a**–**c**), optical microscopy images (**d**–**f**) and ^7^Li solid-state NMR spectra (**g**–**i**) of cycled graphite anodes under LP, OP and HP conditions after 268 cycles transferred under argon. NMR spectra were acquired at a field strength of 16.4 T and a magic angle spinning speed of 30 kHz. Scaling factors (for example, ×1–×32) denote intensity magnification relative to the unscaled spectra to aid visualization of low-intensity features. **j**, Lattice layer distances, defined as half of the graphite lattice parameter *c*, of pristine and cycled graphite anodes under LP, OP and HP conditions after 268 cycles, calculated from post-mortem sXRD patterns. **k**, Post-mortem cross-sectional SEM and ToF-SIMS highlighting electrode morphology and the concentration maps of Li in the cycled graphite anodes under LP, OP and HP conditions after 268 cycles. The colour scale indicates relative Li concentration. Source data

Figure 3a–f and Supplementary Fig. 17 show pictures and optical microscope images of discharged anodes cycled 268 times at different pressures. During disassembly, LP anodes are particularly flaky and seemingly dry, whereas the HP anodes are hard to peel off the separators, with some electrolyte seemingly squeezed out of the electrode area.

Optical microscopy of the OP anodes (12.5 bar, Fig. 3e) shows a relatively homogeneous graphite surface. However, the LP anodes show white regions that might be crystallized electrolyte salt or side products from electrolyte decomposition (see further), as well as some contrasting golden particles (Fig. 3d), which is a signature of LiC_6_. This suggests that some graphite particles were disconnected from the anode in the lithiated state, maybe due to insufficient stack pressure holding the electrode together. This corroborates both the loss of Li inventory and the poorer anode capacity retention observed in DVA (Fig. 2g and Supplementary Fig. 15e). On the other hand, in the case of HP anodes (Fig. 3c), shiny silver-coloured deposits were observed (Fig. 3f). This is indicative of Li plating (see further) and corroborates the sudden electrode expansion at end-of-life and increased slippage loss in DVA. ^7^

Li solid-state NMR spectroscopy was employed to examine the bulk composition of the graphite anodes (Fig. 3g–i). In the spectrum of the graphite extracted from discharged OP cells (Fig. 3h), the ^7^Li signal mainly comes from the SEI (isotropic chemical shift (*δ*_iso_) ~ 0 ppm) with very small contributions from peaks typically assigned to dilute lithiated graphite stages, including stage 3L–4L (LiC_27-40_, *δ*_iso_ = 6.5 ppm) and stage 2L (LiC_18-24_, *δ*_iso_ = 14.5 ppm)^25–27^. In contrast to the OP anode, the discharged LP (Fig. 3g) and HP (Fig. 3i) anodes show strong signals from various lithiated graphite phases including the dense stages LiC_12_/ LiC_6_ (*δ*_iso_ = 45 ppm) and lithium metal (*δ*_iso_ = 265 ppm).

Echoing the NMR results, sXRD confirms that stack pressure influences the state of lithiation of graphite particles in aged, discharged cells. Supplementary Fig. 18 shows characteristic graphite peaks ((002), (004) and (110)), highlighting strong signals from stage 3L and stage 2L (LiC_30_–LiC_18_) for HP samples.

The interlayer spacing parameter *d*_(002)_ was calculated from the sXRD (Fig. 3j), which shows that for OP samples, there is only a slight change compared to pristine graphite, whereas LP and, in particular, HP samples show large increases in *d*_(002)_. While ‘golden’ LiC_6_ graphite particles were not as clearly visible in the microscopy of HP samples, they are likely to be located directly under the plated Li regions and therefore obscured.

To confirm this, we carried out cross-sectional scanning electron microscopy (SEM) imaging and ToF-SIMS mapping of Li on anodes after 268 cycles at different stack pressures (Fig. 3k). First, these cross-sections show the LP anode being thicker than OP and HP cells, in agreement with the tomography analysis above. At OP, the cross-section of the graphite particles shows up as black (fully delithiated as intended) in the SIMS map, with Li signals between the particles originating from the SEI. In the LP samples, there is seemingly more SEI. Similarly, Supplementary Fig. 19 shows SEM images of the LP and OP graphite anodes after 374 cycles, with the LP anode showing a thick, gel-like coating. This may also be indicative of excessive SEI formation, in agreement with the increase in slippage observed in the DVA.

In the HP samples, the strong Li signal at the top of the electrode is in agreement with the observed Li plating (Fig. 3k). In addition, the cross-section of most HP graphite particles is purple (lithiated) rather than black (delithiated).

The plating in HP cells is likely to be the result of impaired lithium-ion transport and increasing local overpotential due to electrolyte being squeezed out of the electrode as noted above during the post-mortem disassembly, along with a decrease in porosity of the anodes and potentially localized separator pore closure at high stack pressure^11,28,29^. This transport limitation in HP cells is further supported by rate performance tests in aged cells (Supplementary Fig. 20), which show a faster capacity loss at high rate than LP and OP cells.

**Fig. 4:**
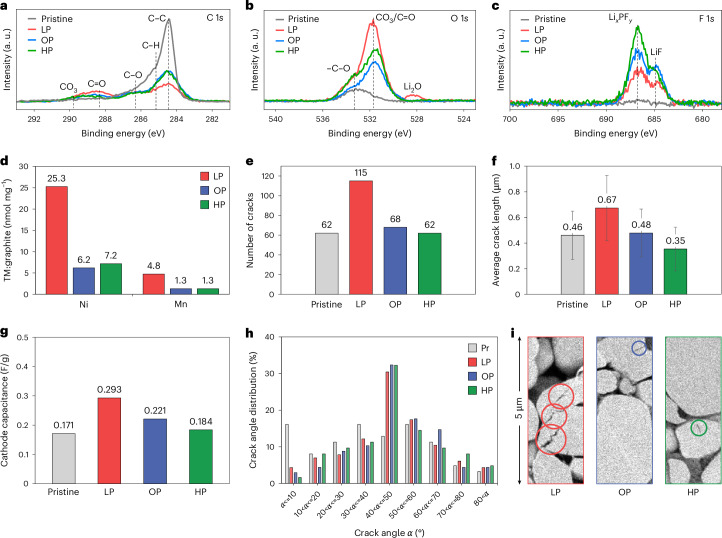
Pressure dependent interfacial degradation and mechanical degradation in cycled electrodes. **a**–**c**, Post-mortem XPS of the C 1*s* (**a**), O 1*s* (**b**) and F 1*s* (**c**) core levels of pristine graphite anodes and cycled graphite anodes under LP, OP and HP conditions after 268 cycles, transferred under argon. Intensities are shown in arbitrary units (a.u.). **d**, MP-AES analysis of TM elements on cycled graphite anodes under LP, OP and HP conditions after 268 cycles. **e**,**f**, Total number of cracks (**e**) and average crack length (**f**), extracted from cross-sectional SEM images of pristine and cycled cathodes under HP (268 cycles), OP (374 cycles) and LP (374 cycles) conditions. Error bars for crack length represent the standard deviation (*n* is the number of cracks in **e**). **g**, Electrode capacitance for pristine, LP (268 cycles), OP (268 cycles) and HP (268 cycles) cathodes measured in blocking conditions using impedance methods^39^. The capacitance values, directly correlated to the specific surface area of the electrodes, were determined at a frequency (*ω*_0_) of 180 mHz. **h**, Distribution of the angle of cracks with the current collector extracted from cross-sectional SEM images of pristine (Pr) and cycled cathodes under HP (268 cycles), OP (374 cycles) and LP (374 cycles) conditions. **i**, Cross-sectional SEM images of the representative cracks highlighted by coloured circles in cycled cathodes under HP (268 cycles), OP (374 cycles), LP (374 cycles) conditions. Source data

Next, we studied the differences in SEI composition with stack pressure using XPS after 268 cycles. The C 1*s* spectra of the LP anode (Fig. 4a) show stronger C=O signals at ~289 eV, indicating a higher concentration of organic species (see fitting results in Supplementary Fig. 21) containing molecular motifs similar to those found in esters or carbonates^30,31^. These organic components are reported to form a more porous and less protective SEI, possibly leading to more lithium-inventory loss over time^22^.

Conversely, the OP and HP anodes exhibit stronger C−C and C−H signals, and furthermore, the OP anode has weaker C−O and CO_3_ peaks. These trends are further confirmed by the O 1*s* spectra (Fig. 4b), where the peaks corresponding to C−O, C=O and CO_3_ species are less intense in the OP anode, indicating less solvent decomposition.

The presence of the Li_2_O signal in LP anodes from the O 1*s* spectra suggests the presence of inorganic species potentially arising from a higher degree of parasitic side reactions. One of the possible pathways includes the reduction of lithium-containing compounds such as lithium formate or reactions involving LiOH (ref. ^32^). The Li 1*s* spectra (Supplementary Fig. 22) also confirm the presence of more Li species for the LP anodes, consistent with more SEI formation.

In the F 1*s* spectra, the OP anodes show a slightly higher intensity for the LiF peak (Fig. 4c and Supplementary Fig. 21f), consistent with a more stable LiF-rich SEI with less coverage by organic species^33^. The peak at 687 eV is attributable to Li_*x*_PF_*y*_ species related to salt degradation, and its higher intensity for the HP anodes may relate to increased degradation associated with the low local potentials reached that induce Li plating.

## Cathode degradation mechanisms and cross-over

We believe there are at least two reasons for the differences in the SEI thickness and composition observed in the LP electrodes. First, the larger anode expansion and contraction in LP cells possibly tensions and shears the SEI (Supplementary Note 5 and Supplementary Fig. 23)^34,35^. Second, cathode TM dissolution and cross-over is higher in LP cells as shown by MP-AES of dissolved SEI. We found higher nickel (Ni) and manganese (Mn) content on LP anodes (Fig. 4d). This suggests an increased dissolution and migration of TM from the cathode, which has been reported to influence SEI composition and accelerate degradation at the anode interface^22,36,37^. This is also confirmed by SEM-energy dispersive X-ray spectroscopy (EDX) mapping (Supplementary Fig. 24) and is linked to the cathode degradation mechanisms (see further) and increased mid-frequency electrochemical impedance spectroscopy (EIS) impedance in LP cells, which is typically assigned to cathode charge transfer resistance (Supplementary Fig. 25)^23,38^.

The increase in TM dissolution at low stack pressure is likely to be linked to an increase in cathode surface area, prompting further investigation of crack signatures in NMC811. First, the extent of cracking is analysed in ion-milled cross-sections (Fig. 4e,f and Supplementary Figs. 26–29), showing both longer and more frequent cracks in LP samples after 374 cycles. Some cathode cracks are known to be much smaller than we can observe with cross-sectional SEM and, therefore, we further verified changes in surface area by a previously reported impedance-based method (Fig. 4g)^39^. This method confirms the above trends, showing a 40% higher increase in electrode area after 268 cycles at LP compared to OP (Supplementary Fig. 30). After cycling, we observed that in all conditions, there is a higher probability of cracks at angles roughly between 40 and 50° to the current collector (Fig. 4h,i). This is not the case in pristine conditions and therefore they are unrelated to calendaring or other manufacturing processes (see the discussion on crack mechanisms below and Supplementary Note 6).

## Discussion

Using high-resolution dilatometry with precise stack-pressure control, we demonstrate a strong correlation between irreversible expansion and degradation, with the onset of accelerated capacity loss coinciding with irreversible swelling.

**Fig. 5:**
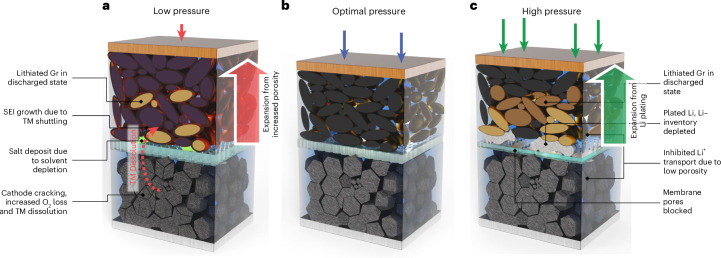
Stack-pressure dependent degradation mechanisms in Gr ‖ NMC811 pouch cells. **a**, Under LP, increased cathode cracking and electrode porosity promote transition metal dissolution, electrolyte decomposition and unstable SEI growth, with anode thickness increase driven by isolation of lithiated graphite. **b**, At OP, balanced interparticle contact and porosity enable stable SEI formation and minimize mechanical degradation. **c**, Under HP, reduced porosity, limited electrolyte access and separator pore blockage hinder ionic transport, leading to accelerated degradation with thickness growth driven by lithium plating.

Figure 5 summarizes the degradation pathways observed under different stack pressures. When the applied pressure is too large (Fig. 5c), the dilatometry reveals a reduction in electrode thickness over initial cycling, followed by a rapid and substantial growth in thickness (Fig. 2e). When the electrodes are at their thinnest, the reduced porosity (Fig. 2f and Supplementary Fig. 27), together with possible local blockage of the separator pores and squeezing of the electrolyte out of the active area^11,28,29^, hamper Li transport (Supplementary Fig. 20). This leads to elevated local overpotential and lithium plating, ultimately increasing the Li-inventory loss through a combination of ‘dead’ Li, SEI and lithiated graphite in discharged conditions as confirmed by NMR, sXRD, ToF-SIMS (Fig. 3) and DVA (Fig. 2g).

By contrast, if the pressure is too low (Fig. 5a), we observe a counter-intuitive increase in cathode particle cracking (Fig. 4e–g). The observation aligns at least in part with the force chain theory developed for granular materials (Supplementary Note 6 and Supplementary Fig. 31)^40^. At low applied pressure (LP), a small number of particles are in physical contact and bear most of the load, leading to high-stress concentrations that could explain increased crack initiation. As stack pressure (*p*_Stack_) increases, the particle coordination $Z$ on average increases according to *Z* = *Z*_0_ + *c*log(*p*_Stack_) (refs. ^41,42^), meaning that more particles come into contact (in agreement with our observed reduction in thickness in OP and HP cells, see Fig. 2e), leading to more parallel force chains and reduced local stress concentration (Hertzian contact, ${P}_{\mathrm{Hertz}}\propto {Z}^{\,-\frac{1}{3}}$)^40–42^, reducing crack formation.

Once cracks initiate, their propagation depends on shear stress. As such, lower pressure results in higher deviatoric stress, promoting crack growth observed between 40 and 50° with the current collector (Fig. 4h)^42,43^, which is the orientation of maximum shear stress under compression. The occurrence of force chains under such angles with the substrate has also been reported in granular media literature^43,44^. Particles with their (003) planes roughly along the orientation of maximum shear stress are more susceptible to cracking^17,45,46^. Higher pressure suppresses deviatoric stress, reducing the likelihood of crack propagation. We note that the behaviour of particles in battery electrodes can deviate from materials studied in classic granular medium theory as they change in volume and mechanical properties with SOC and SOH (Supplementary Note 6).

Overall, the increase in cathode surface area at LP leads to more surface exposed to electrolyte and more cathode oxygen loss, which is recorded indirectly by an increase in TM dissolution by MP-AES (Fig. 4d) and SEM-EDX (Supplementary Fig. 24). According to the literature, oxygen loss is associated with under-coordinated TMs that are then released from the cathode surface^22,47–49^, and is further implied by the elevated cathode interfacial impedance under LP conditions (Supplementary Fig. 25d). TM dissolution is known to lead to faster SEI growth as well as a different anode SEI composition, which is supported here by NMR (Fig. 3g–i), XPS (Fig. 4a–c) and SEM (Supplementary Fig. 19). In addition, the larger expansion and contraction of LP anodes during cycling observed by dilatometry (Supplementary Note 5) might also contribute to SEI layers being sheared off or damaged, resulting in additional SEI formation.

Finally, optical microscopy, NMR and sXRD of LP anodes (Fig. 3) show the presence of lithiated graphite particles in discharged cells, which are probably detached from the electrode. Both excessive SEI-related side reactions and the isolation of lithiated graphite contribute to the loss of lithium inventory and active material capacity in LP cells, as confirmed by our DVA (Fig. 2g and Supplementary Fig. 15). After this Li-inventory loss exceeds a threshold, it has been demonstrated previously that the anode does not return to its lowest voltage plateau at the end of charge, which increases the effective cathode UCV, resulting in further degradation^23^. The above degradation pathway under LP is illustrated in Fig. 5a.

Supplementary Fig. 32 shows the coupling between mechanical parameters and electrochemical degradation, revealing how feedback between the two domains governs the evolution of cell ageing.

Overall, the tool and analysis methods developed in this paper provide clear guidelines for stack-pressure optimization in both academic laboratories and industrial test centres (Supplementary Fig. 33), which are applicable to a wide range of cell formulations (Gr ‖ polycrystalline NMC811 in Supplementary Fig. 34 and Gr ‖ LFP in Supplementary Fig. 35). Furthermore, we anticipate that stack-pressure optimization is synergetic to existing strategies such as electrolyte additives to enhance battery lifetime, as demonstrated by a 96.2% capacity retention after 1,100 cycles in a Gr ‖ single-crystal NMC811 cell cycled to 4.2 V with fluoroethylene carbonate (FEC), VC and LiBF_4_ additives (Supplementary Fig. 36). For larger cells, we propose using two bellow actuators in parallel, which still results in a uniform pressure distribution as demonstrated by pressure paper (Supplementary Fig. 35b). This system can also hold multiple cells in parallel (four 5-Ah cells in Supplementary Fig. 35a) and achieves stable cycling results (Supplementary Fig. 35c,d).

In conclusion, this study shows that the stack pressure plays a more important role in the cycling stability of Li-ion batteries than previously assumed. We introduce a tool that employs a compliant pneumatic actuator capable of maintaining stack-pressure fluctuations below 0.8%, independent of cell expansion. For a representative high-energy LIB chemistry (Gr ‖ NMC811), applying and maintaining a constant stack pressure about four times greater than the typical initial pressure used in coin cells and prismatic formats results in a twofold improvement in battery lifetime. Excessively high stack pressure gradually compresses the cells, ultimately leading to lithium plating on the anode. Conversely, when the pressure is too low, we observe accelerated cracking of the cathode. Optimizing stack pressure balances these degradation pathways, offering a straightforward strategy to extend battery lifespan.

## Methods

### Preparation of pouch cells

Machine made, dry (no pre-filled electrolyte) 402035-size (4 × 20 × 35 mm) multi-layer pouch cells with ~210 mAh nominal capacity and areal capacities of 3.26 mAh cm^−2^ from Li-FUN Technology were used in this study (see Supplementary Table 1 for detailed cell parameters). All pouch cells were filled with 700 μl of standard LP57 electrolyte (1 M LiPF_6_ in ethylene carbonate (EC)/ethyl methyl carbonate (EMC) (3:7, v/v)) with no additives in an Ar-filled glove box and sealed with a pouch sealer. The cells were rested in a climate chamber at room temperature (26 °C) for at least 24 h before formation cycles to facilitate full wetting of the jelly roll.

### Electrochemical characterization

The operando dilatometers were coupled with BioLogic VMP3 systems to perform all formation and charge–discharge cycling with simultaneous thickness measurements. Additional electrochemical cycling, conducted under stack pressure but without thickness sensors, was carried out using a BioLogic BCS-805 system. The formation protocol consisted of three galvanostatic charge–discharge cycles at a C/10 rate (21 mA) within a voltage window of 2.8–4.6 V. After formation, cells were cycled at an increased rate of C/3 (70 mA). In all cycles, there were 60 s rest steps between each charge and discharge to help indicate the polarization in the full cell at the top of charge and bottom of discharge. After every 50 fast cycles, three additional recovery cycles at C/10 constant current (CC) were performed. A minimum of two cells in all stack pressure conditions were tested to ensure reproducibility. All cells were cycled within individual dilatometers placed in a climate chamber (SciQuip Incu-80S) at 26 °C. Electrochemical impedance spectroscopy (EIS) was measured before formation, after formation and during long-term cycling using a Biologic VMP3 potentiostat. Cells were cycled to 3.8 V (constant current at C/10 rate and constant-voltage hold until C/20), followed by a 2 h rest, before EIS was performed at 3.8 V in a climate chamber set at 26 °C with an amplitude of 10 mV and a scanning frequency range between 500 kHz and 10 mHz. The measured EIS data were fitted to the equivalent circuit models using the Python software package impedance.py (ref. ^50^).

### DVA

To better identify the processes contributing most substantially to capacity fading, DVA was applied to the C/10 recovery cycles. This involved plotting d*V/*d*Q* versus capacity and fitting half-cell data to the full-cell data. The double-sided coated electrodes were taken from pristine dry cells in the glove box environment (MBraun, H_2_O and O_2_ levels < 0.5 ppm) and carefully converted into single-sided coated electrodes by scraping and wiping using a sponge and *N*-methyl-2-pyrrolidone (NMP) solvent, then punched into 10 mm diameter circular electrode discs. The standard LP57 electrolyte was used. The cell stack was crimped with an MSK-110 hydraulic crimping machine at 1,100 psi. All assembled cells were rested for 24 h for electrolyte infiltration. The half-cell data were collected using a multichannel BioLogic BCS-805 potentiostat. Constant current charge–discharge cycling at a C-rate of C/20 was performed within the voltage window of 0.005–2.0 V for the graphite anode and 2.5–4.7 V for the NMC811 cathode.

### Characterizations

For post-mortem study, the double-sided coated electrodes were taken from cycled pouch cells, rinsed with dimethyl carbonate and dried. All disassembly procedures were performed in a glove box environment (MBraun, H_2_O and O_2_ levels < 0.5 ppm).

#### Optical microscopy

The optical images of the electrode for post-mortem analysis were collected using an Olympus LC30 3.1-megapixel camera attached to a BX53M optical microscope (Olympus). Each sample was sealed in the Ar-filled glove box (MBraun, H_2_O and O_2_ levels < 0.5 ppm) between a coverslip and a microscope slide using epoxy and then transferred to the microscope. All samples were imaged under constant illumination conditions, constant camera exposure and calibrated white balance.

#### SEM

A Phenom Pro G5 Desktop SEM instrument was used to study the anode surface morphologies of the cycled anodes. A ZEISS Gemini 300 was used to study the cycled cathode surface morphologies. All electrode samples were mounted onto stubs using an adhesive carbon tape and imaged at an accelerating voltage between 5 and 15 kV in secondary electron mode.

#### Cross-sectional SEM of anode samples

The samples were transferred under air-protected conditions from a glove box into a Helios 5 Hydra plasma focused ion beam – SEM system (PFIB-SEM; Thermo Fisher Scientific) for site-specific milling of cross-sections. To protect the top surface from accidental ion beam sputtering, a mixed Pt and C layer was deposited at 20 nA for an initial 0.5 µm layer followed by 70 nA for an additional 2 µm protective layer using a 12 kV Xe^+^ beam. The final polish of the cross-section was done using an Ar^+^ beam with a 4° rocking polish at 30 kV and 0.12 µA. SIMS maps were produced with an Ar^+^ ion beam at 30 kV and 60 pA with a Tofwerk ToF-SIMS.

#### Cross-sectional SEM of cathode samples

The cross-sections were prepared using air-protected transfer from a glove box to a Hitachi IM4000 Plus broad beam ion miller at an acceleration voltage of 6 kV and a discharge voltage of 1.5 kV. The samples were milled for 3 h at 30° rocking angles. Microstructural images were collected on a FIB/SEM Scios Dualbeam equipped with a Hiden quadrupole SIMS.

#### XCT

Tomographic imaging of unopened pouch cells was carried out using the Zeiss Versa 510 system at an excitation voltage of 140 kV, with an optical magnification of 4× and an exposure time of 4 s, achieving a spatial resolution of ~1 µm. A 0.1 mm copper filter was applied at the source. A total of ~2,000 two-dimensional projections (2,032 × 1,966 pixels) were acquired and reconstructed using the filtered back-projection (FBP) method with beam hardening corrections. The resulting tomograms were segmented in VGStudioMax 2024.1 using the Paint and Segment tool, which employs machine learning to streamline the process by allowing users to mark small regions with distinct colours, enabling automatic segmentation based on grey values. Although the grey value differences between many cell components were negligible, the tool proved highly effective. Segmentation quality was iteratively improved through manual adjustments guided by visual feedback, allowing accurate identification of key components such as the anode, cathode and separator. Following segmentation, individual components were extracted as separate regions of interest (ROIs) and assigned false colour labels for visual clarity. Wall thickness analysis was then performed on the segmented volumes using a sphere-fitting algorithm, which randomly places spheres within each region and determines the diameter of the largest possible sphere that fits locally to estimate thickness. The thickness distributions of the anode and cathode were presented as histograms, revealing characteristic differences between pouch samples. The automated thickness measurements were further validated through manual spot checks using geometric measurement tools in VGStudioMax, confirming their accuracy.

#### XPS

The XPS measurements were acquired using an ULVAC Phi Versaprobe III XPS, utilizing a monochromatic Al K_α_ source (1,486.6 eV). The electrodes were transferred from an Ar-filled glove box into the XPS instrument using an inert transfer device, preventing air exposure. The spectra were background subtracted using a Shirley-type background and energy calibrated to the carbon black/hydrocarbon feature set to 284.5 eV. All samples were measured within the same session and their absolute intensities were plotted without further normalization. Curve fitting was performed using the CasaXPS software package, applying Voigt functions with GL(30) peak shape. Relative intensity comparisons were only made within a given core level, meaning relative sensitivity factors did not need to be accounted for.

#### NMR

Samples for NMR experiments were prepared by scraping the discharged and harvested graphite anodes. ^7^Li solid-state NMR (SSNMR) spectra were collected on a Bruker Avance IIIHD spectrometer equipped with a 16.4 T magnet (*ν*_0_(^1^H) = 700.28 MHz, ν_0_(^7^Li) = 272.16 MHz). A Bruker double channel 1.3 mm magic angle spinning (MAS) probe was used with a rotor-synchronized Hahn echo experiment and MAS rate of 30 kHz. Spin-lattice relaxation time constants (*T*_1_) were measured using a saturation recovery experiment and recycle delays were set to five times the longest *T*_1_. ^7^Li chemical shifts were referenced using a capillary of 1 M LiCl aqueous solution (*δ*_iso_ = 0 ppm).

#### MP-AES

Samples for MP-AES experiments were prepared by scraping the discharged and harvested graphite anodes. The anode powder samples were soaked in 4 ml of aqua regia [1 ml of 70% nitric acid (15.7 M):3 ml of 32% hydrochloric acid (10.2 M)] for 10 days. Then 500 μl of the solution was taken and diluted with 3.5 ml of water for each sample for the analysis.

#### sXRD

Materials were scraped off from the graphite anode electrodes and loaded into 0.5 mm diameter quartz capillaries within a glove box. The capillaries were then sealed with two-component epoxy to ensure airtightness. The sXRD experiments were conducted at Beamline I11 of the Diamond Light Source, UK, using an X-ray wavelength of 0.82686 Å.

## Supplementary information

Supplementary Information Supplementary Figs. 1–36, Tables 1–3 and Notes 1–6.

## Source data

Source Data Fig. 1 Statistical source data.

Source Data Fig. 2 Statistical source data.

Source Data Fig. 3 Statistical source data.

Source Data Fig. 4 Statistical source data.

## Data Availability

The data supporting the findings of this study are available within the Article and its Supplementary Information and in the Cambridge Research Repository (Apollo) at 10.17863/CAM.129108. Source data are provided with this paper.

## References

[1] Larcher, D. & Tarascon, J. M. Towards greener and more sustainable batteries for electrical energy storage. *Nat. Chem.* **7**, 19–29 (2015). 25515886 10.1038/nchem.2085

[2] Li, Q. et al. The critical importance of stack pressure in batteries. *Nat. Energy* **10**, 1064–1073 (2025).

[3] Barai, A. et al. The effect of external compressive loads on the cycle lifetime of lithium-ion pouch cells. *J. Energy Storage* **13**, 211–219 (2017).

[4] Laufen, H. et al. Correlation between voltage, strain, and impedance as a function of pressure of a nickel-rich NMC lithium-ion pouch cell. *Adv. Mater. Technol.* **9**, 2301965 (2024).

[5] Mussa, A. S., Klett, M., Lindbergh, G. & Lindström, R. W. Effects of external pressure on the performance and ageing of single-layer lithium-ion pouch cells. *J. Power Sources* **385**, 18–26 (2018).

[6] Müller, V., Scurtu, R. G., Memm, M., Danzer, M. A. & Wohlfahrt-Mehrens, M. Study of the influence of mechanical pressure on the performance and aging of Lithium-ion battery cells. *J. Power Sources* **440**, 227148 (2019).

[7] Li, R. et al. Effect of external pressure and internal stress on battery performance and lifespan. *Energy Storage Mater.* **52**, 395–429 (2022).

[8] Cannarella, J. & Arnold, C. B. Stress evolution and capacity fade in constrained lithium-ion pouch cells. *J. Power Sources* **245**, 745–751 (2014).

[9] Berckmans, G. et al. Electrical characterization and micro X-ray computed tomography analysis of next-generation silicon alloy lithium-ion cells. *World Electr. Veh. J.* **9**, 43 (2018).

[10] Lee, S. G. & Jeon, D. H. Effect of electrode compression on the wettability of lithium-ion batteries. *J. Power Sources* **265**, 363–369 (2014).

[11] Peabody, C. & Arnold, C. B. The role of mechanically induced separator creep in lithium-ion battery capacity fade. *J. Power Sources* **196**, 8147–8153 (2011).

[12] Spingler, F. B., Kücher, S., Phillips, R., Moyassari, E. & Jossen, A. Electrochemically stable in situ dilatometry of NMC, NCA and graphite electrodes for lithium-ion cells compared to XRD measurements. *J. Electrochem. Soc.* **168**, 040515 (2021).

[13] Prado, A. Y. R., Rodrigues, M.-T. F., Trask, S. E., Shaw, L. & Abraham, D. P. Electrochemical dilatometry of Si-bearing electrodes: dimensional changes and experiment design. *J. Electrochem. Soc.* **167**, 160551 (2020).

[14] Michael, H. et al. A Dilatometric study of graphite electrodes during cycling with X-ray computed tomography. *J. Electrochem. Soc.* **168**, 010507 (2021).

[15] Daubinger, P., Ebert, F., Hartmann, S. & Giffin, G. A. Impact of electrochemical and mechanical interactions on lithium-ion battery performance investigated by operando dilatometry. *J. Power Sources* **488**, 229457 (2021).

[16] Stallard, J. C. et al. Mechanical properties of cathode materials for lithium-ion batteries. *Joule* **6**, 984–1007 (2022).

[17] Stallard, J. C. et al. Effect of lithiation upon the shear strength of NMC811 single crystals. *J. Electrochem. Soc.* **169**, 040511 (2022).

[18] Chen, Z. et al. Detection of jelly roll pressure evolution in large-format Li-ion batteries via in situ thin film flexible pressure sensors. *J. Power Sources* **566**, 232960 (2023).

[19] Xu, C., Reeves, P. J., Jacquet, Q. & Grey, C. P. Phase behavior during electrochemical cycling of Ni-rich cathode materials for Li-ion batteries. *Adv. Energy Mater.* **11**, 2003404 (2021).

[20] Märker, K., Reeves, P. J., Xu, C., Griffith, K. J. & Grey, C. P. Evolution of structure and lithium dynamics in LiNi_0.8_Mn_0.1_Co_0.1_O_2_ (NMC811) cathodes during electrochemical cycling. *Chem. Mater.* **31**, 2545–2554 (2019).

[21] Nadimpalli, S. P. V., Sethuraman, V. A., Abraham, D. P., Bower, A. F. & Guduru, P. R. Stress evolution in lithium-ion composite electrodes during electrochemical cycling and resulting internal pressures on the cell casing. *J. Electrochem. Soc.* **162**, A2656–A2663 (2015).

[22] Björklund, E. et al. Cycle-induced interfacial degradation and transition-metal cross-over in LiNi_0.8_Mn_0.1_Co_0.1_O_2_-graphite cells. *Chem. Mater.* **34**, 2034–2048 (2022). 35557994 10.1021/acs.chemmater.1c02722 PMC9082506

[23] Dose, W. M., Xu, C., Grey, C. P. & De Volder, M. F. L. Effect of anode slippage on cathode cutoff potential and degradation mechanisms in Ni-rich Li-ion batteries. *Cell Reports Phys. Sci.* **1**, 100253 (2020).

[24] Bloom, I. et al. Differential voltage analyses of high-power, lithium-ion cells 1. Technique and application. *J. Power Sources* **139**, 295–303 (2005).

[25] Maxwell, D. C., O’Keefe, C. A., Xu, C. & Grey, C. P. ^13^C NMR study of the electronic structure of lithiated graphite. *Phys. Rev. Mater.* **7**, 65402 (2023).

[26] Zaghib, K. et al. 7Li - NMR of well-graphitized vapor-grown carbon fibers and natural graphite negative electrodes of rechargeable lithium-ion batteries. *J. Electrochem. Soc.* **146**, 2784–2793 (1999).

[27] Letellier, M., Chevallier, F. & Morcrette, M. In situ ^7^Li nuclear magnetic resonance observation of the electrochemical intercalation of lithium in graphite; 1st cycle. *Carbon N. Y.* **45**, 1025–1034 (2007).

[28] Koo, J. K. et al. Detrimental electrochemical behavior caused by excessive high pressure on Li-ion pouch-type full cell. *Electrochem. Commun.* **152**, 107518 (2023).

[29] Müller, V. et al. Effects of mechanical compression on the aging and the expansion behavior of Si/C-composite|NMC811 in different lithium-ion battery cell formats. *J. Electrochem. Soc.* **166**, A3796–A3805 (2019).

[30] Boulanger, P., Riga, J., Delhalle, J. & Verbist, J. J. An XPS study of hexagonal polyoxymethylene with various bulk morphologies: surface modification under X-ray exposure. *Polymer (Guildf)* **29**, 797–801 (1988).

[31] Laoharojanaphand, P., Lin, T. J. & Stoffer, J. O. Glow discharge polymerization of reactive functional silanes on poly(methyl methacrylate). *J. Appl. Polym. Sci.* **40**, 369–384 (1990).

[32] Rinkel, B. L. D., Hall, D. S., Temprano, I. & Grey, C. P. Electrolyte oxidation pathways in lithium-ion batteries. *J. Am. Chem. Soc.* **142**, 15058–15074 (2020). 32697590 10.1021/jacs.0c06363

[33] Phelan, C. M. E. et al. Role of salt concentration in stabilizing charged Ni-rich cathode interfaces in Li-ion batteries. *Chem. Mater.* **36**, 3334–3344 (2024). 38617803 10.1021/acs.chemmater.4c00004 PMC11008099

[34] Niu, S. et al. Analysis on the effect of external press force on the performance of LiNi_0.8_Co_0.1_Mn_0.1_O_2_/graphite large pouch cells. *J. Energy Storage* **44**, 103425 (2021).

[35] Dong, G. & Wei, J. A physics-based aging model for lithium-ion battery with coupled chemical/mechanical degradation mechanisms. *Electrochim. Acta* **395**, 139133 (2021).

[36] Pieczonka, N. P. W. et al. Understanding transition-metal dissolution behavior in LiNi_0.5_Mn_1.5_O_4_ high-voltage spinel for lithium ion batteries. *J. Phys. Chem. C* **117**, 15947–15957 (2013).

[37] Jung, R. et al. Nickel, manganese, and cobalt dissolution from Ni-rich NMC and their effects on NMC622-graphite cells. *J. Electrochem. Soc.* **166**, A378–A389 (2019).

[38] Gilbert, J. A. et al. Cycling behavior of NCM523/graphite lithium-ion cells in the 3–4.4 V range: diagnostic studies of full cells and harvested electrodes. *J. Electrochem. Soc.* **164**, A6054–A6065 (2017).

[39] Oswald, S., Pritzl, D., Wetjen, M. & Gasteiger, H. A. Novel method for monitoring the electrochemical capacitance by in situ impedance spectroscopy as indicator for particle cracking of nickel-rich NCMs: Part I. Theory and validation. *J. Electrochem. Soc.* **167**, 100511 (2020).

[40] Peters, J. F., Muthuswamy, M., Wibowo, J. & Tordesillas, A. Characterization of force chains in granular material. *Phys. Rev. E* **72**, 041307 (2005). 10.1103/PhysRevE.72.041307 16383373

[41] Agnolin, I. & Roux, J. N. Internal states of model isotropic granular packings. I. Assembling process, geometry, and contact networks. *Phys. Rev. E* **76**, 061302 (2007). 10.1103/PhysRevE.76.061302 18233840

[42] Roux, J. N. Geometric origin of mechanical properties of granular materials. *Phys. Rev. E* **61**, 6802–6836 (2000). 10.1103/physreve.61.6802 11088375

[43] Zhang, L. et al. The role of force chains in granular materials: from statics to dynamics. *Eur. J. Environ. Civ. Eng.* **21**, 874–895 (2017).

[44] Mandal, R., Casert, C. & Sollich, P. Robust prediction of force chains in jammed solids using graph neural networks. *Nat. Commun.* **13**, 4424 (2022). 35908018 10.1038/s41467-022-31732-3 PMC9338954

[45] Yan, P. et al. Intragranular cracking as a critical barrier for high-voltage usage of layer-structured cathode for lithium-ion batteries. *Nat. Commun.* **8**, 14101 (2017). 28091602 10.1038/ncomms14101 PMC5241805

[46] Morzy, J. K. et al. Origins and importance of intragranular cracking in layered lithium transition metal oxide cathodes. *ACS Appl. Energy Mater.* **7**, 3945–3956 (2024). 38756866 10.1021/acsaem.4c00279 PMC11094680

[47] Dose, W. M. et al. Onset potential for electrolyte oxidation and Ni-rich cathode degradation in lithium-ion batteries. *ACS Energy Lett.* **7**, 3524–3530 (2022). 36277132 10.1021/acsenergylett.2c01722 PMC9578037

[48] Wandt, J., Freiberg, A. T. S., Ogrodnik, A. & Gasteiger, H. A. Singlet oxygen evolution from layered transition metal oxide cathode materials and its implications for lithium-ion batteries. *Mater. Today* **21**, 825–833 (2018).

[49] Ruff, Z., Xu, C. & Grey, C. P. Transition metal dissolution and degradation in NMC811-graphite electrochemical cells. *J. Electrochem. Soc.* **168**, 060518 (2021).

[50] Murbach, M., Gerwe, B., Dawson-Elli, N. & Tsui, L. impedance.py: A Python package for electrochemical impedance analysis. *J. Open Source Softw.* **5**, 2349 (2020).

