# Supplementary material for: The interplay between stack pressure, mechanical expansion and degradation pathways in lithium-ion batteries
Source: Nat Energy. 2026 Jun 29;11(7):1032–42. doi: 10.1038/s41560-026-02087-6 (PMC13391380; doi:10.1038/s41560-026-02087-6)
Supplement: Supplementary file 1 — Supplementary Figs. 1–36, Tables 1–3 and Notes 1–6. [file 41560_2026_2087_MOESM1_ESM.pdf]

nature energy

Supplementary information https://doi.org/10.1038/s41560-026-02087-6

The interplay between stack pressure, mechanical expansion and degradation pathways in lithium-ion batteries

In the format provided by the authors and unedited

Supplementary Information

Table of Contents

Supplementary Fig. 1: Different methods for applying stack pressure to pouch cells
Supplementary Fig. 2: Long-term cycling performance under different pressure-application rigs
Supplementary Fig. 3: Comparison of spring-loaded and pneumatic bellow stack pressure systems
Supplementary Fig. 4: Representative baseline pressures in coin cells
Supplementary Fig. 5: Cross-sectional view of our dilatometer
Supplementary Fig. 6: Pouch cell structure and constant pressure distribution
Supplementary Fig. 7: Initial expansion analysis
Supplementary Fig. 8: Long-term cycling performance of cells with or without 2 wt% VC electrolyte
Supplementary Fig. 9: Electrochemical and mechanical response with different upper cut-off voltages
Supplementary Fig. 10: Additional dilatometry and electrochemistry data
Supplementary Fig. 11: Long-term cycling capacity for individual XLP, LP, MP, OP, and HP cells
Supplementary Fig. 12: Cumulative irreversible cell expansion for individual LP, OP, and HP cells
Supplementary Fig. 13: False colour 3D reconstruction of all XCT results
Supplementary Fig. 14: Micrometre screw gauge measurement of anode thickness
Supplementary Fig. 15: DVA for diagnosing capacity loss from active material loss
Supplementary Fig. 16: Additional differential expansion and differential voltage cures
Supplementary Fig. 17: Additional anode optical microscope images
Supplementary Fig. 18: Post-mortem sXRD patterns of pristine and cycled graphite anodes
Supplementary Fig. 19: Post-mortem SEM images of graphite anode surface morphology
Supplementary Fig. 20: Rate capability test performance under LP, OP and HP conditions
Supplementary Fig. 21: Fittings of XPS spectra of graphite anodes after 268 cycles
Supplementary Fig. 22: Additional XPS spectra of graphite anodes after 268 cycles
Supplementary Fig. 23: Analysis of hysteresis in anode dilation
Supplementary Fig. 24: EDX of graphite anode samples
Supplementary Fig. 25: EIS fitting results for LP, OP, and HP cells during long-term cycling
Supplementary Fig. 26: Cross-sectional SEM of cathode samples with segmented particle boundaries
Supplementary Fig. 27: Post-mortem cathode thickness and density analysis
Supplementary Fig. 28: Cathode particle grain boundary separation analysis
Supplementary Fig. 29: Additional cross-sectional SEM images highlighting cathode particle cracking
Supplementary Fig. 30: Surface area estimation of LP, OP and HP cathode samples after 268 cycles
Supplementary Fig. 31: Crack initiation and propagation mechanism
Supplementary Fig. 32: Relationship between mechanical and electrochemical parameters
Supplementary Fig. 33: Pictograms illustrating the three key applications of our tool
Supplementary Fig. 34: Long-term cycling performance of PC-NMC811/graphite cells
Supplementary Fig. 35: Demonstration of scale-up module for 5-Ah LFP/graphite pouch cells
Supplementary Fig. 36: Long-term cycling performance of OP cell at a 4.2 V UCV

Supplementary Table. 1: Pouch cell specifications
Supplementary Table. 2: Key dilatometer features
Supplementary Table. 3: Summary of phases and phase transitions captured by differential curves

Supplementary Note. 1: Different Methods for Applying Stack Pressure to Pouch Cells
Supplementary Note. 2: Details of Our Dilatometry Experimental Setup
Supplementary Note. 3: Effect of Upper Cut-off Voltage on Cycle Life and the Optimal Pressure
Supplementary Note. 4: Differential Voltage Analysis
Supplementary Note. 5: Analysis of Hysteresis in Anode Dilation
Supplementary Note. 6: A Mechanistic Explanation for Crack Initiation and Propagation

Supplementary Note. 1 | Different Methods for Applying Stack Pressure to Pouch Cells

There are three standard formats of cells with liquid electrolytes widely used in the electric vehicle industry: pouch, cylindrical, and prismatic.[1,2] The format, along with the winding technique, profoundly impacts the initial physical boundary conditions that electrode stacks are subjected to. In cylindrical and prismatic cells, stack pressure is applied by the jellyroll winding and the module itself.[3,4] In pouch cells, external fixtures and mechanical joints are typically required to ensure sufficient initial stack pressure. The majority of these mechanical constraints apply stack pressure by fixing the thickness of the cell.[5–7]

Existing methods for applying stack pressures to pouch cells, as summarised in Supplementary Fig. 1, fall into three categories: Fixed Displacement, Spring-loaded Pressure, and Pneumatic Pressure.

Constant Gap or Fixed Displacement: As the most commonly adopted technique for pouch cell testing and formation,[7–10] fixed displacement is typically achieved through simple mechanical joints, such as bolts and nuts, to sandwich pouch cells between two rigid plates.

Spring-loaded Pressure: This is the semi-constant pressure setup that aims to address the significant pressure variations in fixed displacement setups by introducing springs to control the initial stack pressure.[10–13] This increasingly popular approach mitigates drastic stack pressure variation during cell cycling. However, the improvement is limited since the force exerted by the spring still varies as the electrodes deform during cycling. This would result in much less repeatable dilatometry data as the long-term degradation-related thickness growth could still significantly impact the mechanical properties of the electrodes. In addition, if the springs are not calibrated carefully to similar k-values, inhomogeneous loading across the surface could arise over time, resulting in poorer data consistency.

Pneumatic Pressure: The most advanced setups utilise pneumatic actuators, which provide a constant pressure independent of volume changes in the cell to maintain constant pressure, which would decouple the stack pressure applied to the pouch cells from the thickness change.[5,6,14] This setup accommodates the long-term irreversible displacement, thus relieving self-generated pressure within the pouch cell to ensure the same stack pressure conditions are applied from the start to the end of the long-term cycling, resulting in more reliable and repeatable dilation and electrochemical cycling data.

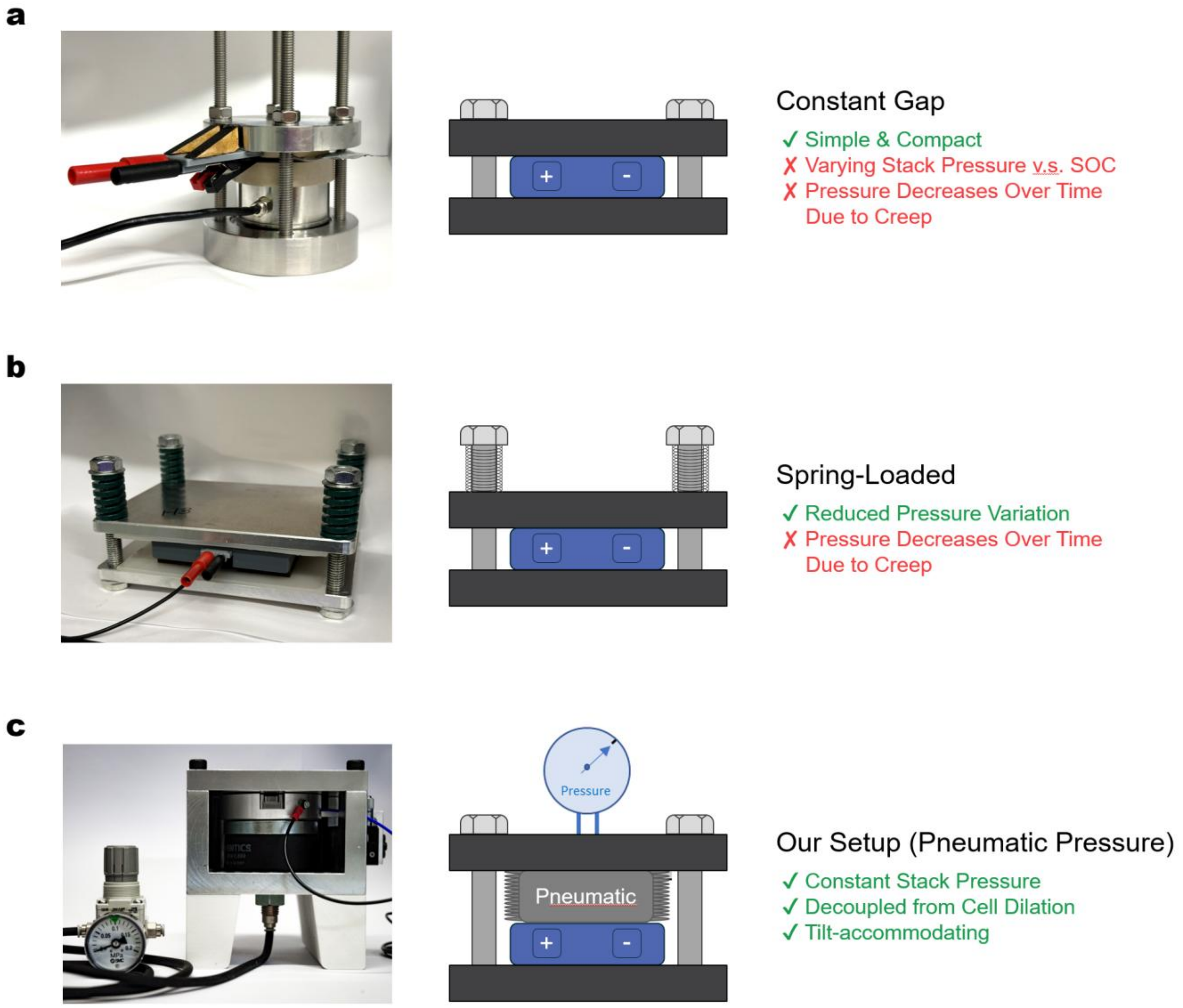

Supplementary Fig. 1 | Different methods for applying stack pressure to pouch cells (from left to right: photos of representative setup, schematics, competitive analysis). (a) Constant-gap setup. (b) Spring-loaded setup. (c) Pneumatic-pressure setup (this work).

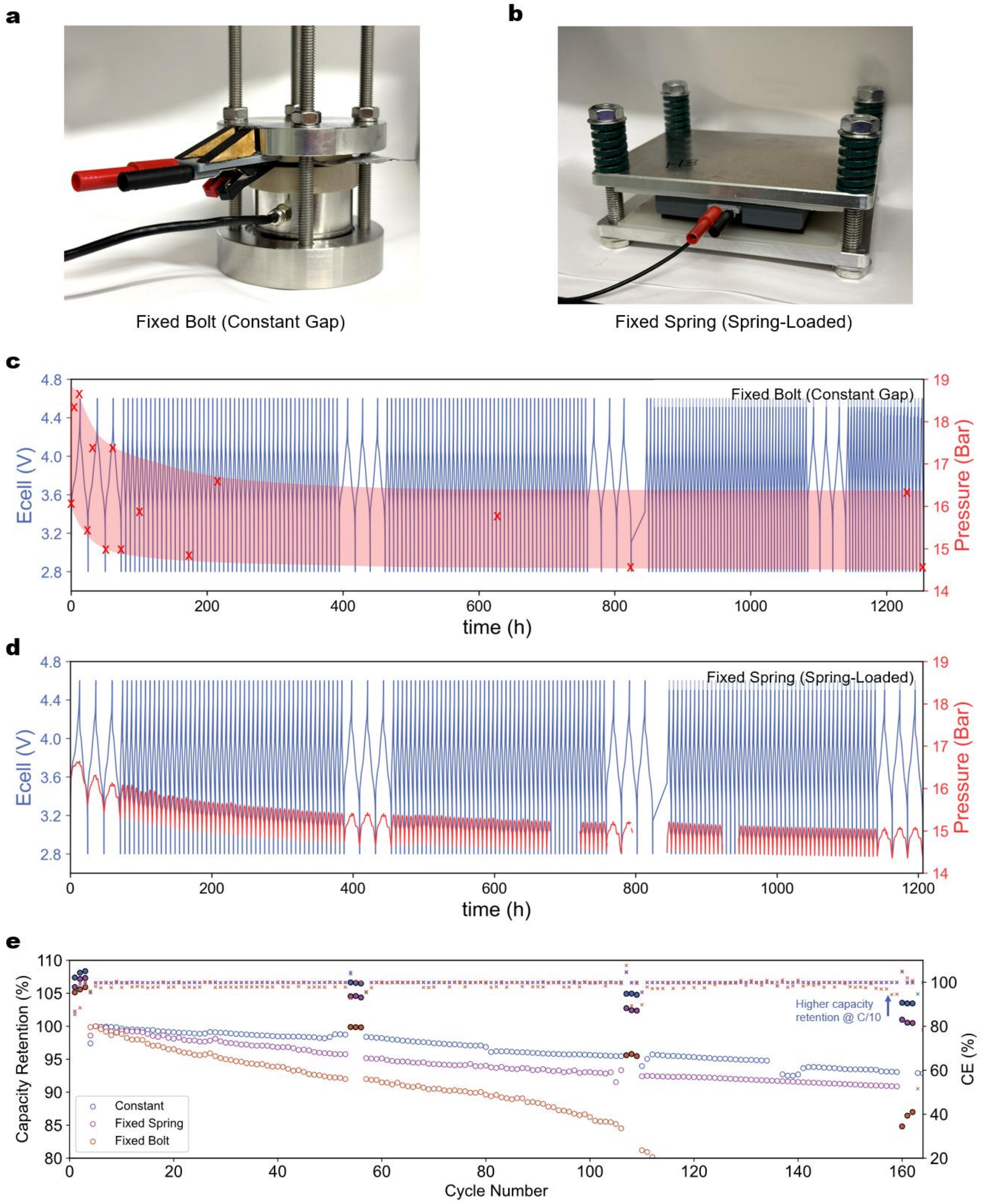

Supplementary Fig. 2 | Long-term cycling performance of graphite || single-crystal NMC811 pouch cells under different pressure-application rigs. (a) Commercially available fixed four-bolt stack pressure rigs for solid-state cell testing, equipped with a readout to monitor the applied pressure. (b) Commercially available spring-based stack pressure test rig, integrated with a custom-built pressure sensor for real-time stack pressure measurement. Cell voltage profile (blue) and stack pressure reading (red) under (c) fixed four-bolt rig, where red crosses show real-time pressure value and shaded areas show estimated pressure range, and (d) spring-based rigs, where the solid red lines show the in-situ reading from the pressure sensor. (e) Long-term capacity retention and coulombic efficiency (CE) of cells tested under different pressure rigs, with constant pneumatic pressure rigs showing significantly better retention after 162 cycles. All cells were cycled between 2.8 V and 4.6 V in a standard LP57 electrolyte (1 M $LiPF_6$ in 3: 7 EC/EMC) with no additives.

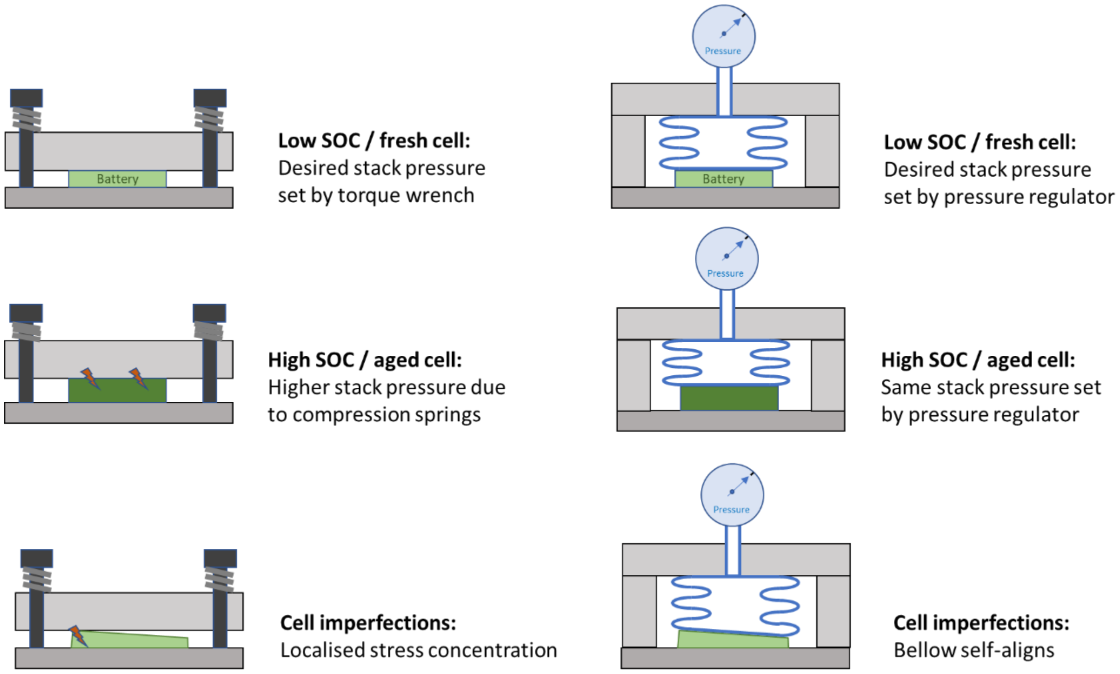

Supplementary Fig. 3 | Comparison of classic spring-loaded and our pneumatic bellow stack pressure systems. Left: Current stack pressure control systems (spring-loaded) and issues they experience with increased stack pressure at high state of charge, aged cells, and cells with non-uniform thickness or manufacturing imperfections.[14–16] Right: Our pneumatic bellow actuators with schematic illustrations of how they avoid limitations in current designs.

**Name**	**S204070C**	
**Cell Type**	**402035-size**	
**Nominal Capacity**	210 mAh	
**Cell Dimension (L x W x T)**	29mm*18mm*4mm	
**Voltage Window**	2.8-4.6V	
**Folding**	Jellyroll-rolling	
	**Cathode**	**Anode**
**Active materials**	Single-Cystal NMC811	AG
**AM Ratio**	95.50%	94.80%
**AM Loading (mg/cm^2)**	16.71	11.3
**Specific Capacity (mAh/g) @3.0 V-4.3 V, 0.2C.**	195	340
**Press Density (g/cc)**	3.3	1.5
**Single Side Coating Thickness (µm)**	50.5	76.5
**Number of Layers**	17 layers<br>3 Single-coated<br>7 Double-coated	18 layers<br>2 Single-coated<br>8 Double-coated
**Coating Dimension (L x W)**	Side A: 169mm*26mm<br>Side B: 127mm*26mm	Side A: 170mm*28mm<br>Side B: 141mm*28mm
**Total Area (mm^2)**	7696	8708
**Theoretical Capacity (mAh)**	250.8	334.6
**Actual Capacity @0.1C (mAh)**	230	N.A.
**Current Collector**	Al	Cu
**Thickness of Current Collector (µm)**	12	8

Supplementary Table. 1 | Pouch cell specifications.

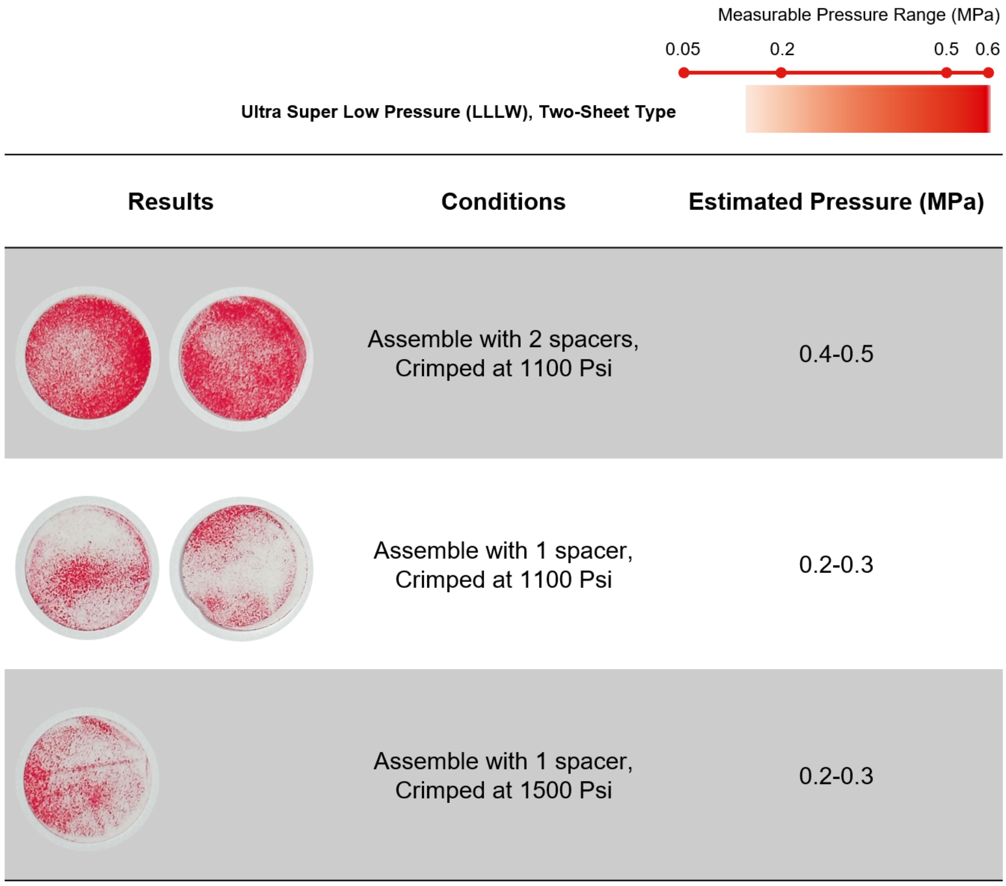

Supplementary Fig. 4 | Representative baseline pressures in coin cells: Pressure was measured by crimping a 2-sheet type LLLW Fujifilm Prescale Pressure Paper (colour scale by Fujifilm[17]) within the coin cell set-up. The resulting colour distribution indicates the pressure magnitude, based on the calibrated colour scale provided by the manufacturer.

Supplementary Note. 2 | Compliant Pneumatic Pressure Electrochemical Dilatometer

Our Compliant Pneumatic Pressure Electrochemical Dilatometer (CPP-ECD) further improves upon the state-of-the-art pressure application setup by incorporating a compliant rubber gasket and a tilt-accommodating bellow actuator (Aventics), as shown in Supplementary Fig. 3 and Supplementary Fig. 5. These additions facilitate more homogeneous stack pressure across the tested pouch cell surfaces (see Supplementary Fig. 6b), minimising the impact from potential edge-to-edge thickness variation and pressure points arising from manufacturing imperfections such as Kapton tapes on the jellyroll.[14–16]

The Micro-Epsilon displacement sensor (CSH05FL-CRm1,4 Capacitive Sensor, flat type) is positioned at the centre of each pouch cell target. With a manufacturer-specified resolution of 0.38 nm (at 2 Hz), a measurement range of 0.5 mm, linearity of less than 0.09 µm, and drift of less than 100 nm/month, this sensor is crucial for precise measurements. To maintain a consistent sensor distance across various experimental settings, 6.5 mm travel translation stages (MS2S/M) from Thorlabs are incorporated to fine-tune the sensor position. The tilt angle of the sensor probe was kept within 0.2° using a dial test indicator. All dilatometers operate within a climate chamber (SciQuip Incu-80S) set to 26°C to minimise temperature variation. To ensure measurements are taken under equilibrium temperature and pressure conditions, the setup rests for 24 hours at the beginning of every new round of experiments. See Supplementary Table. 2 for an overview of all the system's components and their respective functions. The key advantages of our system are summarised below:

- **Stable Pressure and High-Resolution Displacement Sensor**: The simultaneous use of a pneumatic loading system and a capacitive displacement sensor allows accurate, drift-free measurement of cell expansion, decoupled from mechanical noise and pressure variability (see Supplementary Fig. 3).
- **Long-Term Operation**: The system is thermally and mechanically stable for > 3000 hours of continuous operation—allowing degradation studies under realistic cycling conditions.
- **Designed for Pouch Cells**: Accommodates real-world, multi-layer pouch cells with consideration for surface irregularities, misalignment, and stack height variability.
- **Customisability**: Modular design allows adjustments in pressure range, cell format, and sensor positioning for different experiment types.

With the accurate in-situ measurement capability of our dilatometer, users can map the real-time expansion of the target cell under electrochemical cycling against cell voltage, capacity, or various other cycling parameters. In the case of Main Fig. 1b, expansion was plotted against the state-of-charge (SOC) capacity by first synchronising the expansion–time data with the electrochemical data. As a constant-current protocol was used, the capacity at each point was determined automatically by the potentiostat software based on the applied current and elapsed time. During discharge, the SOC capacity was calculated by subtracting the discharge capacity at a given time from the maximum charge capacity of the cycle. This allows the dilatometry data to be accurately aligned with electrochemical states throughout the cycle, enabling detailed analysis of material behaviour.

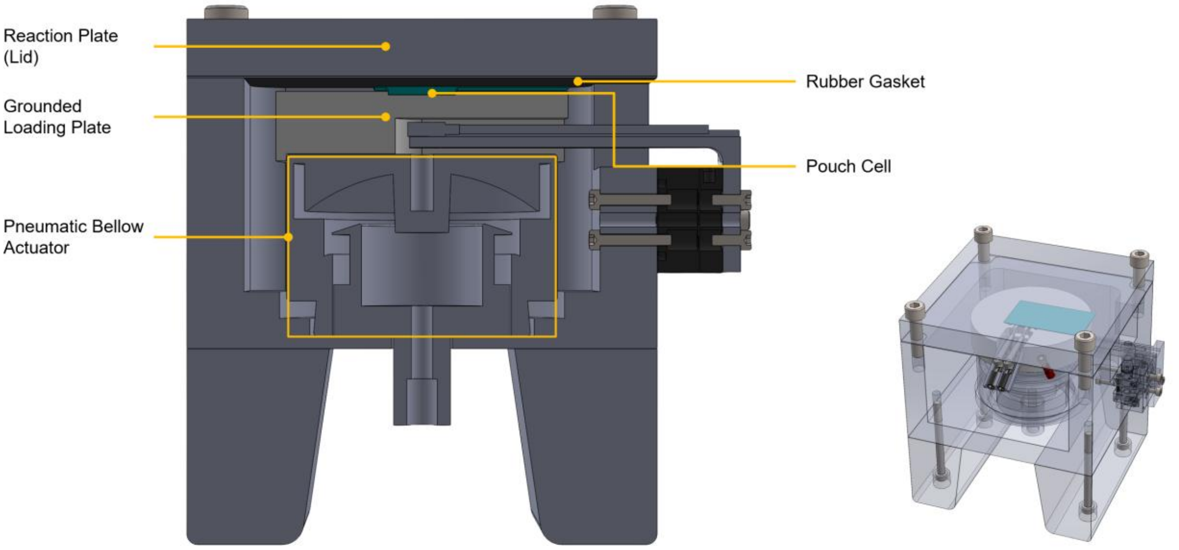

Supplementary Fig. 5 | Cross-sectional view of our dilatometer, highlighting the positioning of key components.

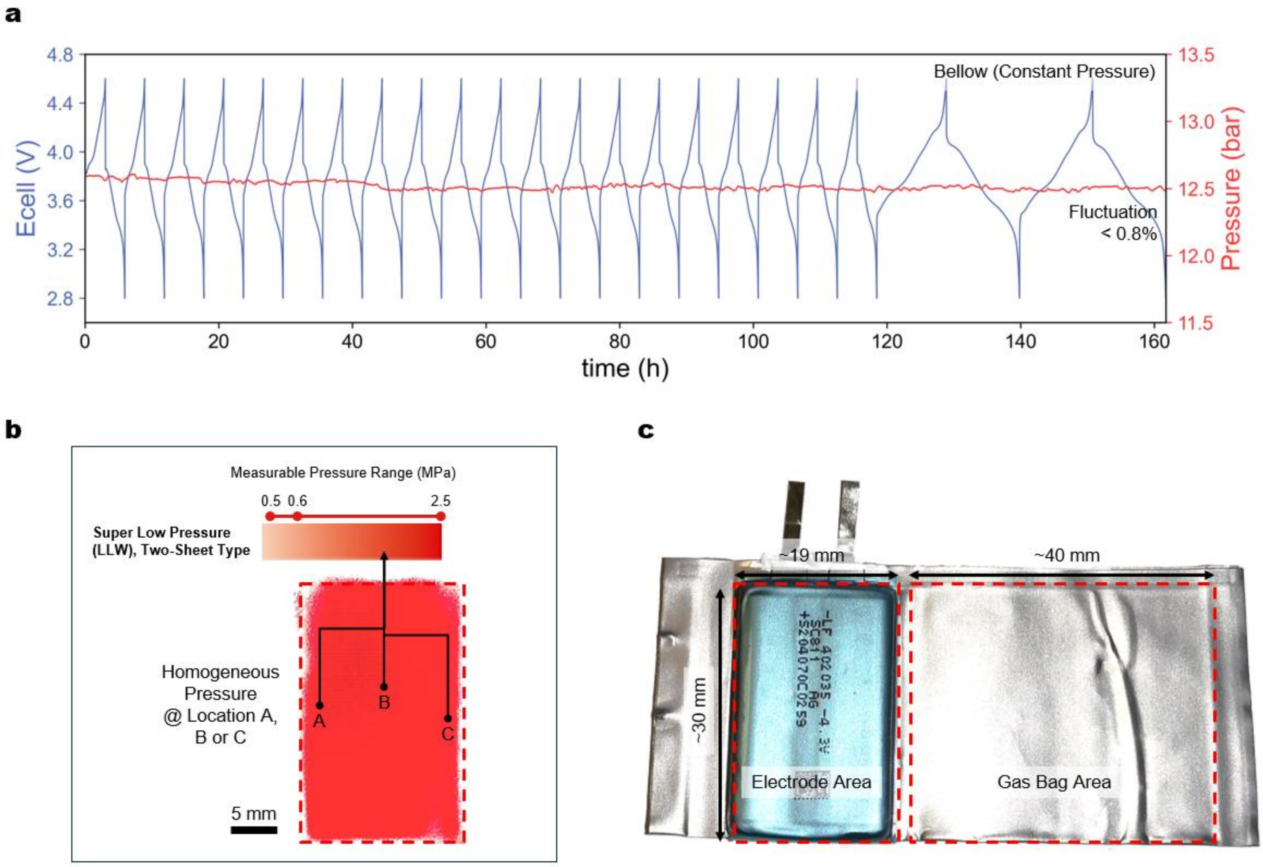

Supplementary Fig. 6| Pouch cell structure and constant pressure distribution: (a) Cell voltage profile (blue) and stack pressure reading (red) under our constant pressure below the rig, where the solid red lines show the in-situ reading from the pressure sensor. The result demonstrates constant pressure over 160 hours of cycling. (b) Pressure paper test demonstrating homogeneous stack pressure across the pouch cell electrode area under OP. (c) Photo of pouch cell showing distinct electrode (19 mm × 30 mm) and gas bag (40 mm × 30 mm) areas, outlined by red dashed lines. The electrode area contains active materials for electrochemical reactions, while the gas bag accommodates gas generated during operation.

Component	Feature / Specification	Role
**Pneumatic Bellow Actuator**	Smooth, stable loading with 2 rotational DOF	Maintains uniform stack pressure; accommodates minor cell tilt
**Capacitive Sensor (Micro-Epsilon CSH05FL)**	Resolution: 0.38 nm; Drift: <100 nm/month	Detects cell expansion with high precision
**Translation Stage (Thorlabs MS2S/M)**	6.5 mm adjustment range	Aligns the sensor within the optimal measurement range
**Pressure Regulator and Valve**	Fine pneumatic control	Enables independent pressure stability during long-term cycling
**Frame, Reaction Plate (Lid), Legs**	Machined and bolted assembly	Provides a rigid, stable support structure
**Compliant Rubber Gasket**	Flexible interface	Helps distribute pressure evenly across the pouch cell surface
**Grounding Ring**	Earthing the loading plate	Reduces electrical noise for accurate sensor readout
**Climate Chamber (SciQuip Incu-80S)**	26°C setpoint	Minimises thermal fluctuation over the test duration

Supplementary Table. 2 | Key dilatometer features.

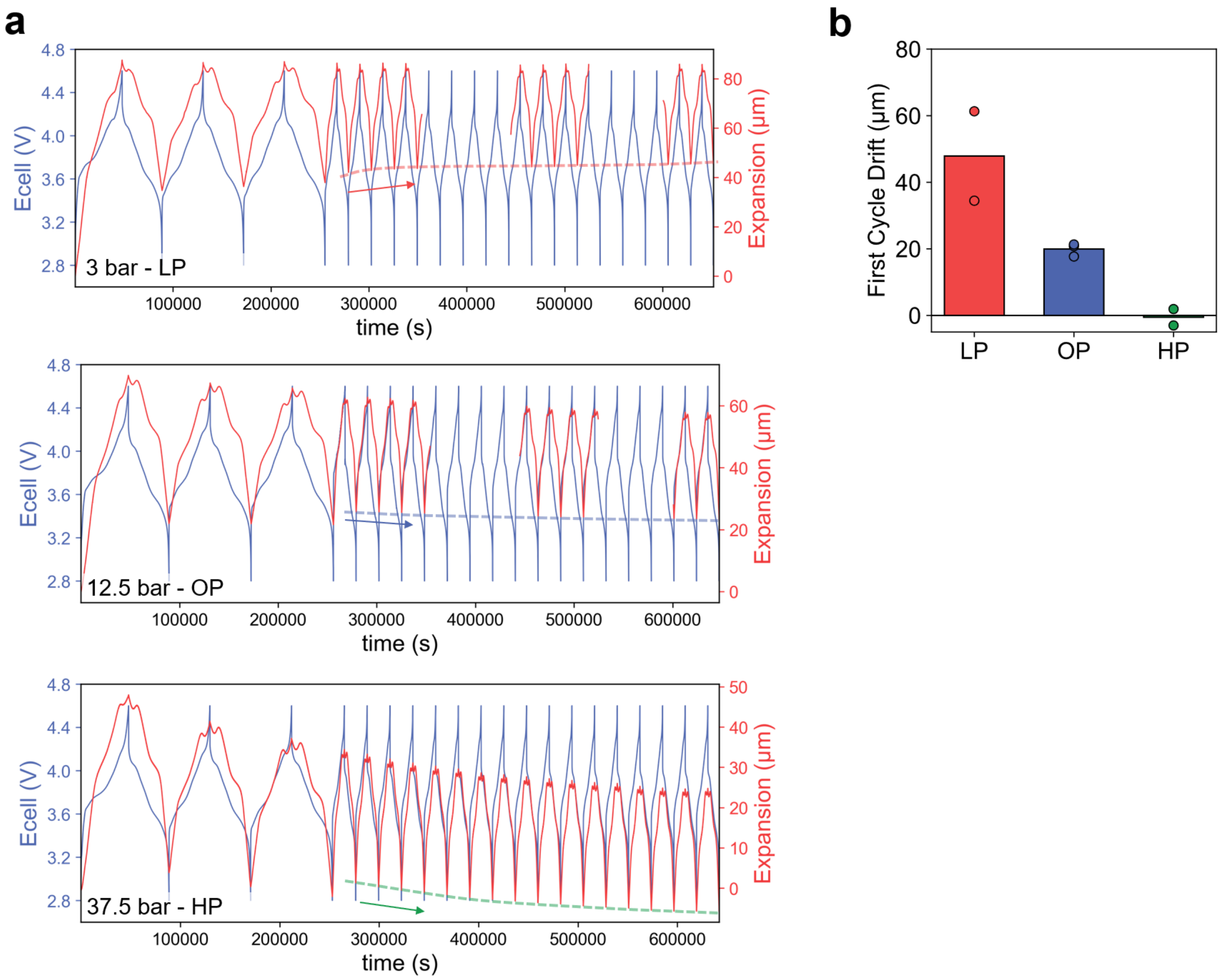

Supplementary Fig. 7 | Initial expansion analysis: (a) Cell voltage profile (blue) and thickness expansion profile (red) during the initial three C/10 (21 mA) slow formation cycles and subsequent 17 C/3 (70 mA) normal cycles under LP, OP, HP stack pressure conditions. (b) First-cycle drift under LP, OP and HP. Bars represent mean values calculated from the individual data points shown.

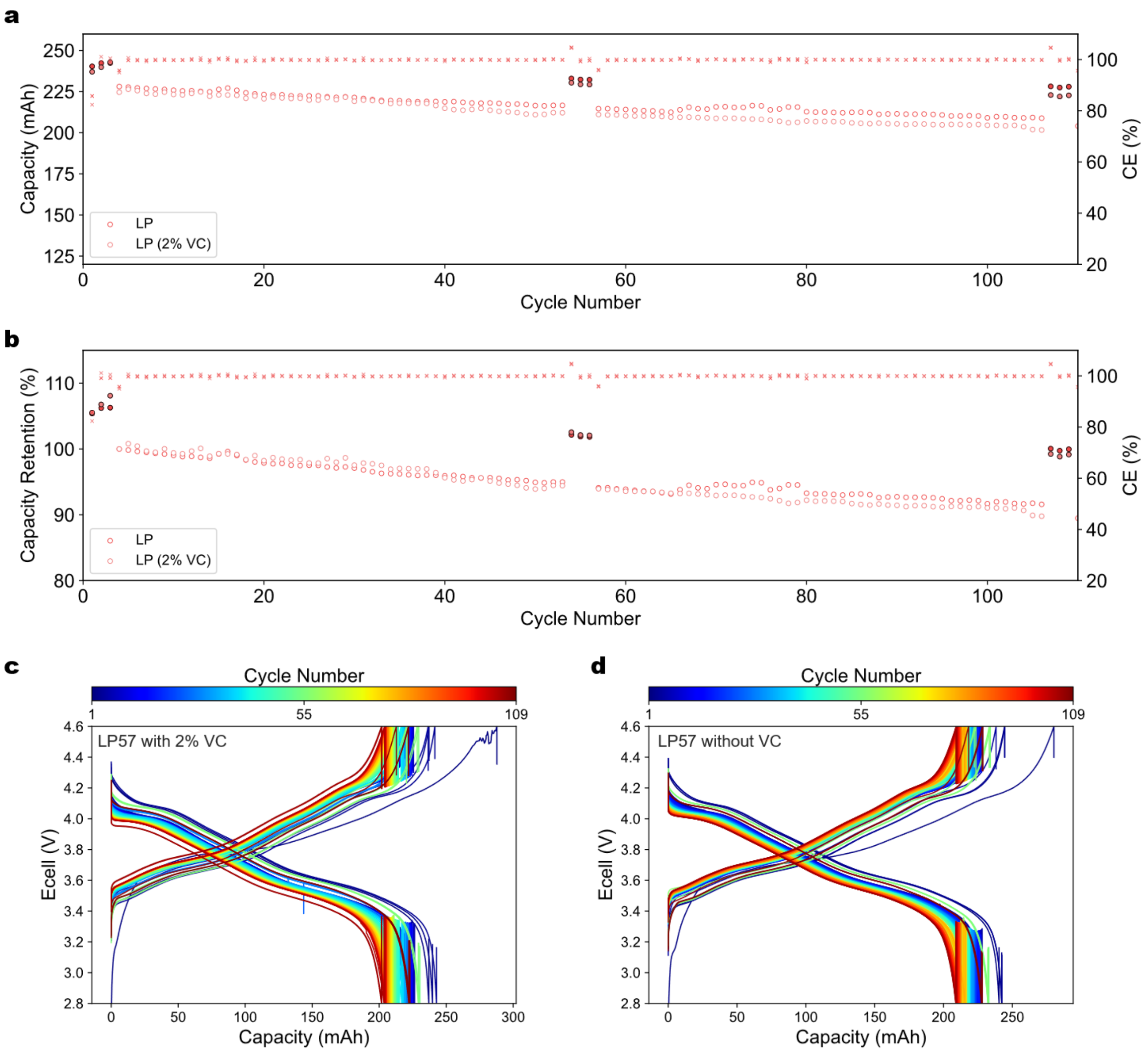

Supplementary Fig. 8 | Long-term cycling performance of cells with LP57 electrolyte or LP57 + 2 wt% VC electrolyte, cycled under LP condition at a 4.6 V upper cut-off voltage (UCV). (a) Discharge capacity and coulombic efficiency (CE). (b) Capacity retention. Voltage profiles of (c) first 109 cycles with VC and (d) first 109 cycles without VC.

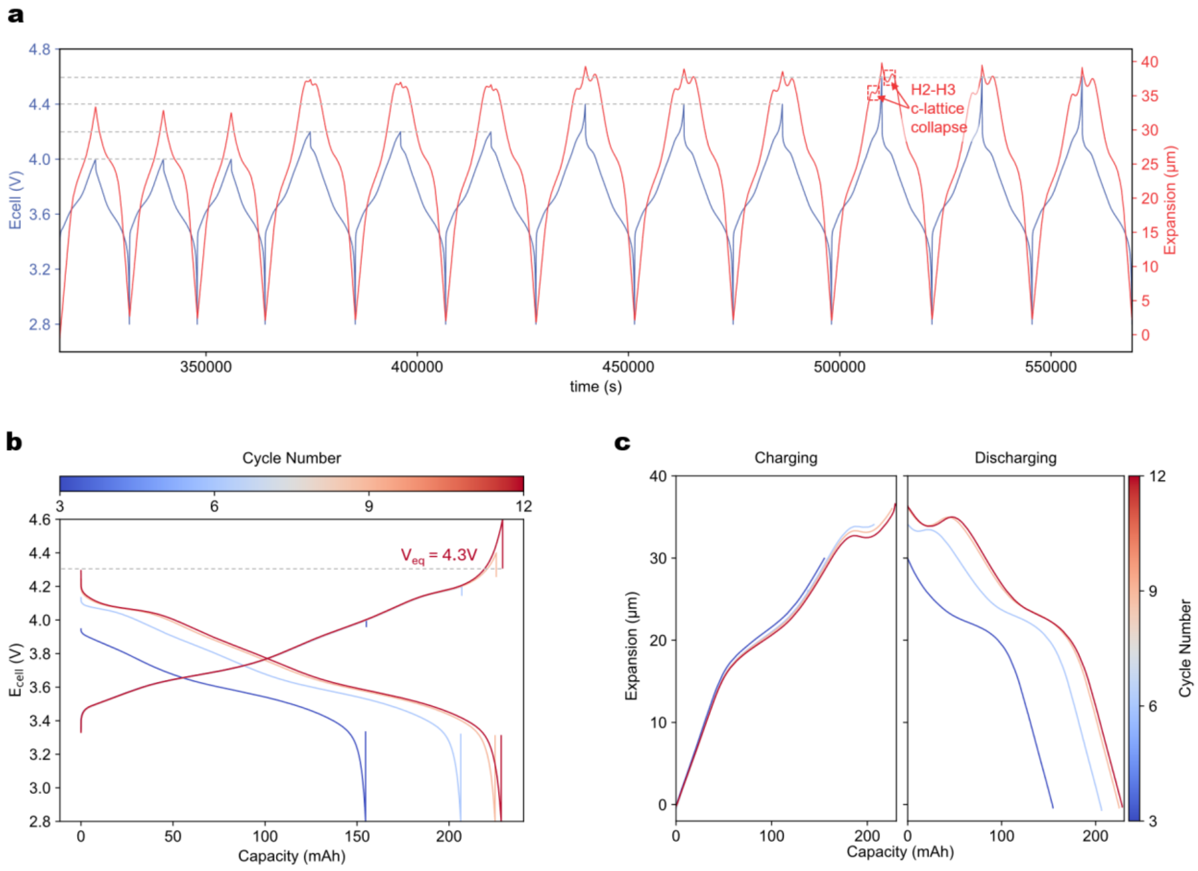

Supplementary Fig. 9 | Electrochemical and mechanical response with different upper cut-off voltages (UCV) of 4.0 V, 4.2 V, 4.4 V, and 4.6 V of a newly formed NMC811/graphite pouch cell cycled with optimal pressure (OP) conditions at C/3. (a) Voltage (blue) and electrode thickness expansion (red) as a function of time. A sharp contraction in electrode thickness is more clearly observed at the top of charge under UCV of 4.6V, corresponding to the H2–H3 transition and associated c-lattice collapse, as highlighted. (b) Voltage profiles plotted against capacity, coloured by cycle number. The profiles highlight a relatively high overpotential of approximately 200–400 mV for the cell charged to 4.6V, as indicated by the voltage drop observed during the 60-second rest period following the end of charge. (c) Thickness expansion as a function of capacity during charging and discharging.

Supplementary Note. 3 | Effect of Upper Cut-off Voltage on Cycle Life and the Optimal Pressure

It is well established that NMC811 cathodes are mechanically weakest at high states of charge (SOC), particularly at the top of charge under high UCV conditions.[18,19] Specifically, our previous work reported a 55% reduction in the shear strength of Single Crystal $Li_yNi_{0.8}Mn_{0.1}Co_{0.1}O_2$ cathode — dropping from 86 ± 12 MPa in the fully lithiated state (at y = 1) to 39 ± 5 MPa at high SOC (at y = 0.14).[19] As a result, lowering the UCV is expected to mitigate this mechanical weakening and thereby reduce the extent of degradation. Indeed, as shown later in Supplementary Fig. 36, the cell cycled under optimal pressure at a UCV of 4.2 V exhibits even milder degradation than those cycled to 4.6 V, supporting this hypothesis. While lowering the UCV clearly offers further lifetime benefits, this does not access important pressure-related degradation mechanisms, and the resulting extension in cycle life would make a systematic long-term degradation study impractical within a reasonable experimental timeframe. We therefore focus on higher UCV conditions, where degradation processes can be meaningfully interrogated using the limited number of available high-resolution dilatometry platforms.

Our long-term cycling experiments have shown that the optimal pressure for graphite ‖ single-crystal NMC811 cells cycled to a 4.6 V upper cut-off voltage (UCV) is approximately 12.5 bar. This naturally raises the question of whether this optimal pressure shifts when the voltage window is reduced. While the NMC cathode may tolerate higher stack pressures more readily at lower UCVs, which would further mitigate cathode-side degradation, the overall pressure tolerance of the full cell may still be limited by other components, such as the separator or anode. For example, even though the cathode shows no additional cracking at HP (see Main Fig. 4e–f), such high pressure may still lead to undesirable porosity reduction or separator pore closure in the cell.[15,20,21] Therefore, despite the possibility of further improving the structure stability of the cathode at lower voltages with even higher stack pressure, the previously identified optimal pressure may remain appropriate when balancing mechanical and electrochemical stability across the full cell architecture.

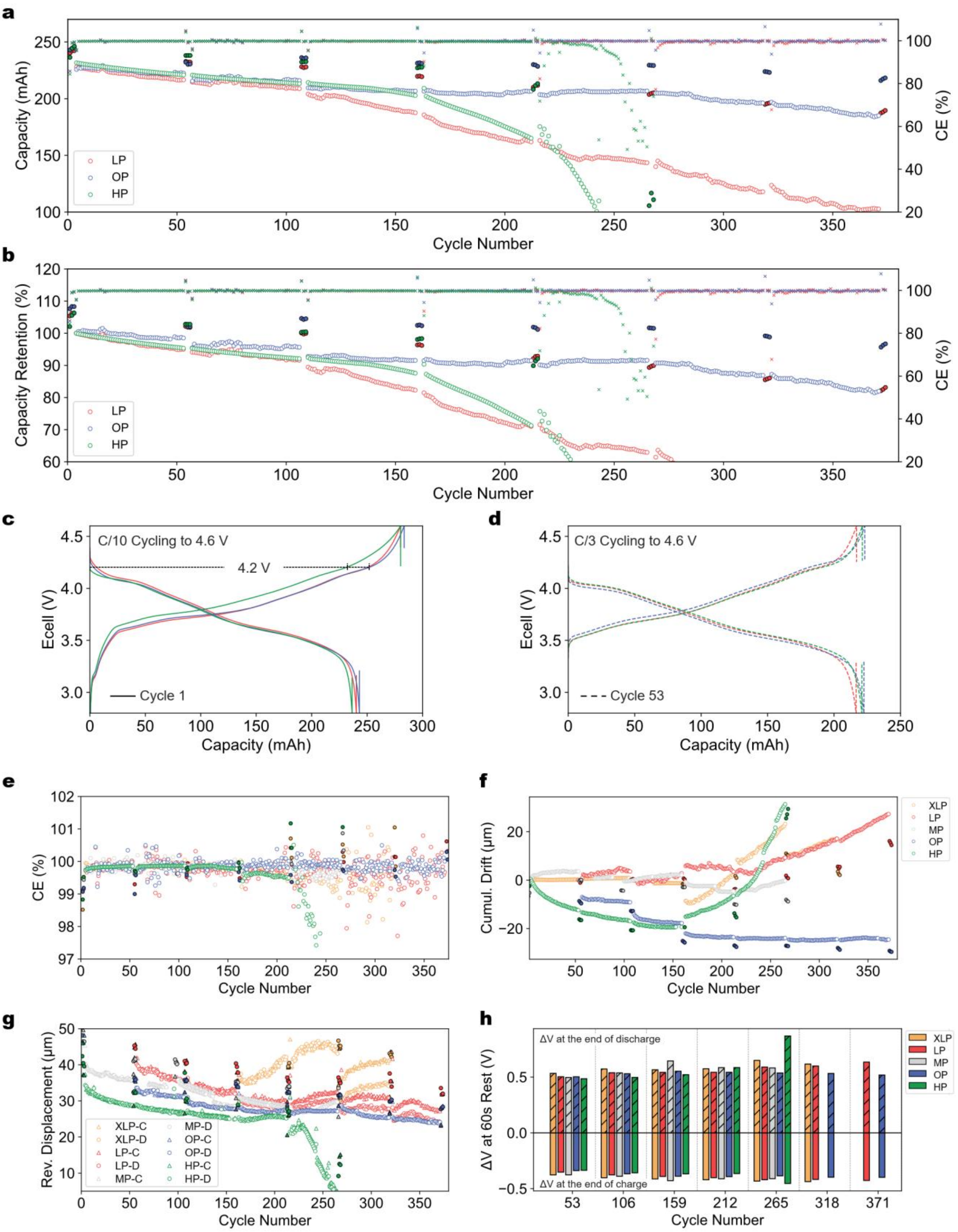

Supplementary Fig. 10 | Additional dilatometry and electrochemistry data: (a) Discharge capacity and coulombic efficiency (CE), (b) capacity retention from the long-term cycling of LP, OP and HP cells at a 4.6 V upper cut-off voltage. Charge–discharge voltage profiles of LP, OP and HP for (c) the first C/10 formation cycle, and (d) an early C/3 cycle (Cycle 53). In the first cycle, the charge capacities in all test conditions are all within 1.1% (LP: 280.2mAh; OP: 283.4mAh; HP: 280.2mAh) and the discharge capacities within 2.7% difference (LP: 240.3mAh; OP: 243.0mAh; HP: 236.5mAh). (e) Zoomed-in columbic efficiency, (f) irreversible expansion, (g) reversible expansion and (h) voltage drop during 60s rest

step at the end of every charge and discharge during representative C/3 cycles, under XLP, LP, MP, OP, and HP conditions. [Note the XLP cell showed negligible true expansion (panel g) before cycle 162 because low stack pressure prevented plate–electrode contact, so the dilatometer mostly measured gas-bag swelling instead.]

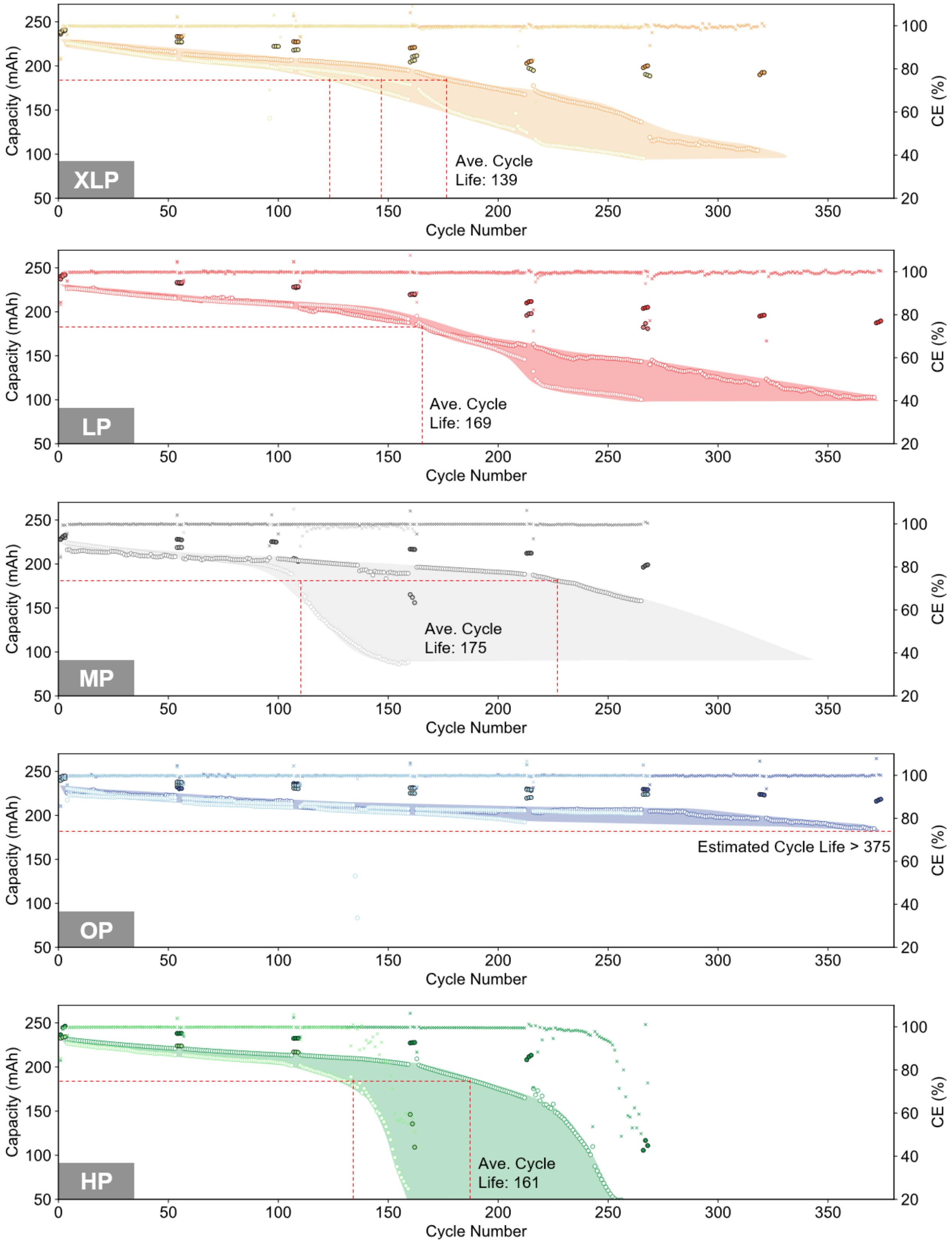

Supplementary Fig. 11 | Long-term cycling capacity for individual XLP, LP, MP, OP, and HP cells (with shades representing the range).

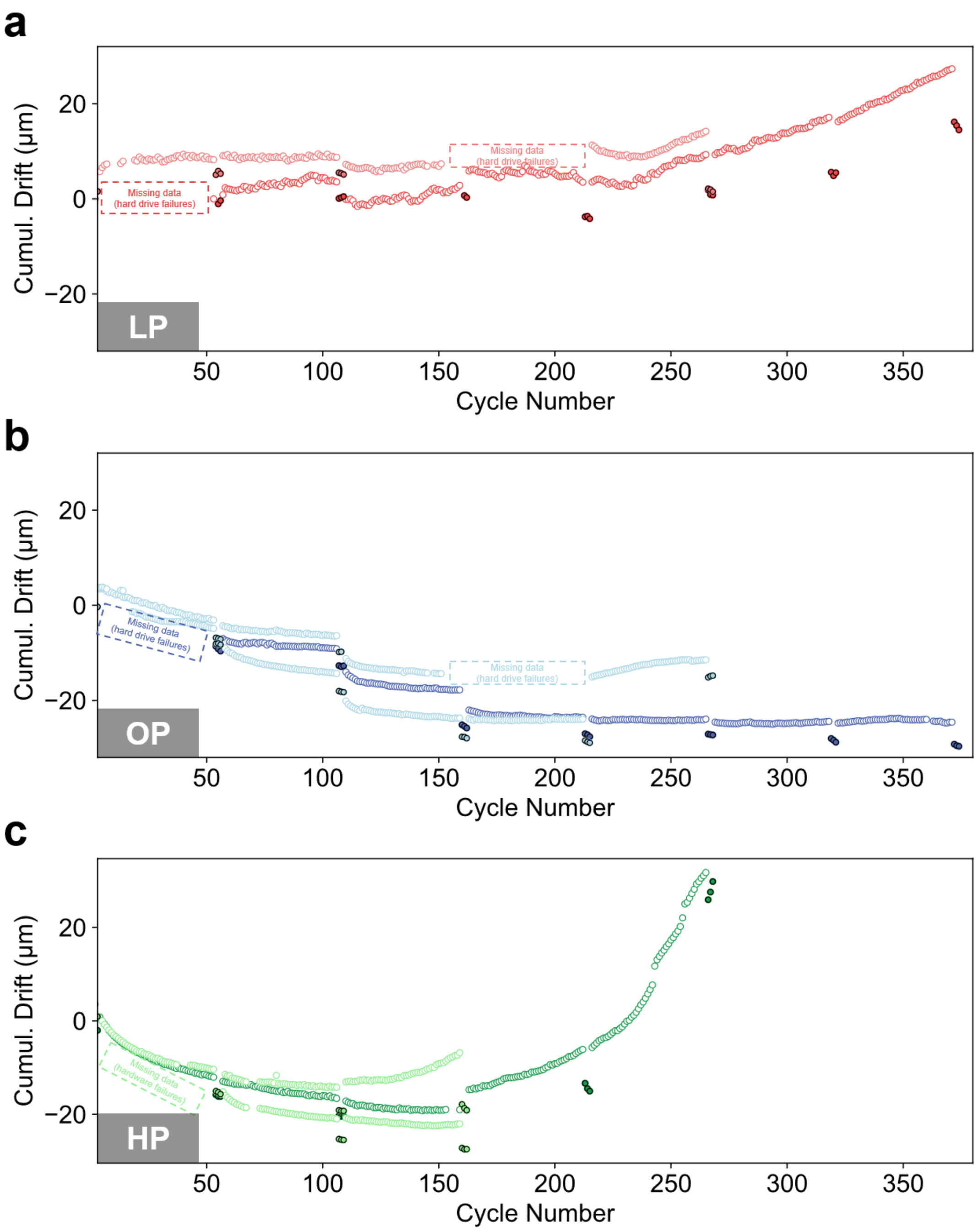

Supplementary Fig. 12 | Cumulative irreversible cell expansion for LP, OP, and HP cells: Data trends are demonstrated using repeated measurements.

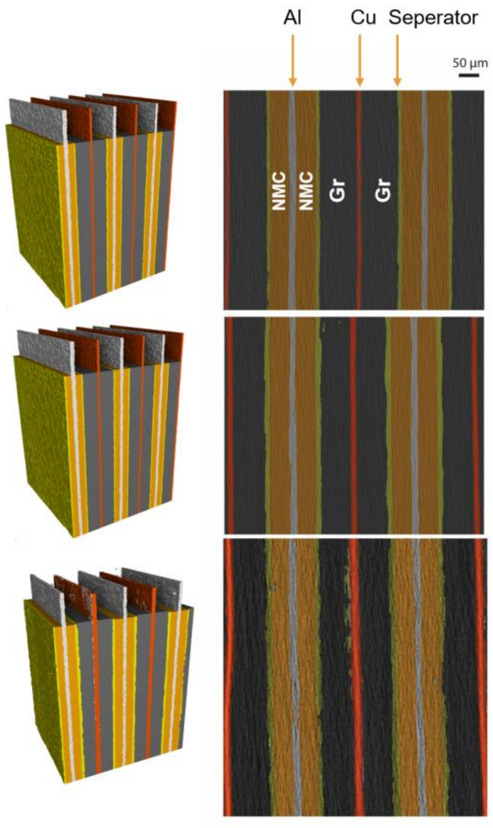

LP	
Anode	~89 µm
Cathode	~45 µm
Al	~20 µm
Separator	~10 µm
Cu	~12 µm

OP	
Anode	~67 µm
Cathode	~44 µm
Al	~20 µm
Separator	~10 µm
Cu	~12 µm

HP	
Anode	~72 µm
Cathode	~44 µm
Al	~22 µm
Separator	~12 µm
Cu	~11 µm

Supplementary Fig. 13 | False colour 3D reconstruction of all XCT results on anodes under LP, OP, HP after 268 cycles.

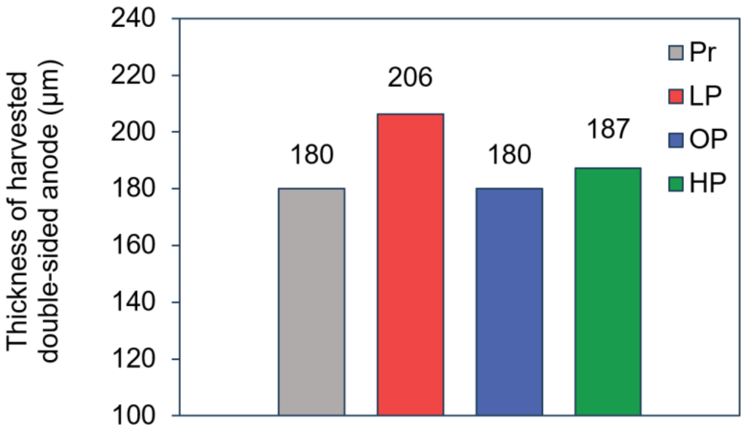

Supplementary Fig. 14 | Thickness of pristine and cycled LP, OP, HP anodes (after 268 cycles) measured using micrometre screw gauge.

Supplementary Note. 4 | Differential Voltage Analysis

Cathode active material loss and lithium inventory loss (also referred to as "slippage") were identified using Differential Voltage Analysis (DVA), which leverages voltage steps occurring as the anode and cathode change SOC (typically measured first in half-cells).[22–24] Tracking the positions of voltage transitions using dV/dQ plots allows deconvoluting capacity fade in the cathode, anode and slippage in full-cells. This technique involves aligning and scaling the voltage–capacity derivative curves (dV/dQ) obtained from individual NMC811 cathode and graphite anode half-cells (cycling protocol detailed in the methodology section) to reconstruct the full-cell response, minimising the error between experimental and modelled full-cell data. This methodology is consistent with our prior studies conducted on similar NMC811/graphite systems,[25,26] and is implemented using the relationships[22–24]:

$$V_{cell} = V_{cathode} - V_{anode} \quad (1)$$

$$\left(\frac{dV}{dQ}\right)_{cell} = \left(\frac{dV}{dQ}\right)_{cathode} - \left(\frac{dV}{dQ}\right)_{anode} \quad (2)$$

To ensure sufficient resolution in identifying dQ/dV peaks (Supplementary Fig. 15a) and reduce impedance-related distortion, 3 diagnostic cycles at C/10 are incorporated in our cycling protocol after every 50 standard cycles at C/3. Specifically, the third cycle within each diagnostic set—performed every 53 cycles—was used as the primary reference for DVA. Our C/3 data does not voltage transitions sufficiently clearly to extract cathode or anode transitions clearly. We plot the Cycle 3 formation cycle (LP) and Cycle 215 under optimal-pressure (OP) operation, both of which exhibited distinct anode (b, d, h) and cathode features (g, j), as shown in Supplementary Table. 3 and Supplementary Fig. 15b-c. The independently obtained half-cell reference data for both NMC811 and graphite electrodes show good feature alignment with the actual full cell.

The key benefit of DVA is that it enables the assignment of individual features in the full-cell voltage profile to the respective electrodes, facilitating a quantitative evaluation of two different modes of degradation:

(a) Electrode slippage reflects the misalignment of anode and cathode voltage profiles over time, attributed primarily to the irreversible consumption of lithium—especially during SEI growth and repair on the graphite electrode—as previously described in foundational studies by Bloom et al.[22–24]

(b) In contrast, capacity fade from either cathode or anode is represented by the compression of its respective half-cell dV/dQ curve along the capacity axis (i.e. a scalar multiplier to the half-cell capacity), indicating a reduction in active material. To further isolate the origin of degradation, we fitted a separate parameter for high-voltage loss in the cathode to capture capacity loss occurring specifically at voltages exceeding 4.1 V vs. Li/Li^+ in accordance with literature.[25,26]

A comprehensive discussion of how each fitting parameter influences the modelled voltage derivatives is available in both Bloom et al.[22–24] and our previous work.[25,26]

To illustrate the DVA approach, the full-cell capacity-normalised differential voltage (Q dV/dQ) is plotted in Supplementary Fig. 15b-c. Supplementary Fig. 15d compares dV/dQ curves at Cycles 3 and 215 for LP, OP, and HP cells. The cathodic peak separation between features *g* and *j*, associated with voltage transitions in the NMC811 cathode, narrows slightly from Cycle 3 to 215—most prominently under LP—indicating low-voltage cathode capacity loss. All cells also show a more pronounced compression of feature *k*, suggesting a dominant high-voltage cathode degradation. In parallel, a rightward shift of the anodic peak *h* (graphite stage 2 lithiation) in the OP cell indicates slippage loss due to anodic side reactions. Overall, the results demonstrate that LP conditions lead to greater anode capacity loss (Supplementary Fig. 15e) and more significant cathode degradation above 4.1 V (Supplementary Fig. 15f), underscoring the influence of stack pressure on distinct degradation modes.

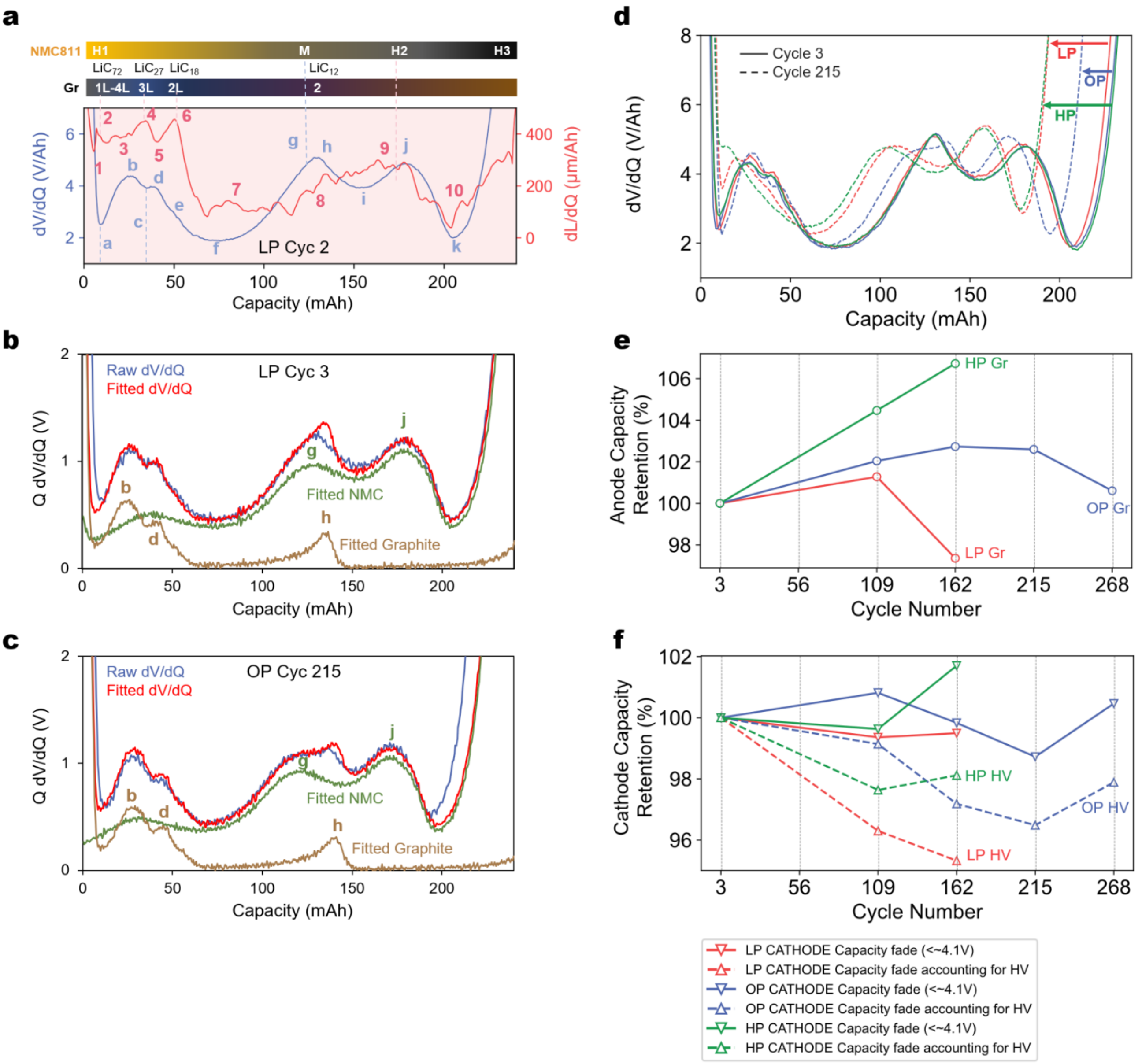

Supplementary Fig. 15 | Differential voltage analysis (DVA) for diagnosing capacity loss from active material loss in LP, OP, and HP cells: (a) Representative DVA and differential expansion (dL/dQ) curves from LP cell at Cycle 2, annotated with characteristic peaks corresponding to graphite (Gr) and NMC811 phase transitions. (b) Illustration specific fitted half-cell data for DVA on LP cell Cycle 3. (c) Illustration specific fitted half-cell data for DVA on OP cell Cycle 215. (d) Comparison of dV/dQ profiles between Cycle 3 and Cycle 215 for LP, OP, and HP cells, showing peak shifts indicative of electrode degradation and active material loss. (e) Anode (graphite) capacity retention over cycling extracted from peak fitting of DVA curves. The HP cell shows improved graphite capacity retention, while the LP cell experiences loss at Cycle 162. (f) Cathode (NMC811) capacity retention split into low-voltage (<4.1 V) and high-voltage (>4.1 V, dominating) regions. The LP cell exhibits pronounced capacity loss at high voltage, suggesting loss of electrochemically active NMC.

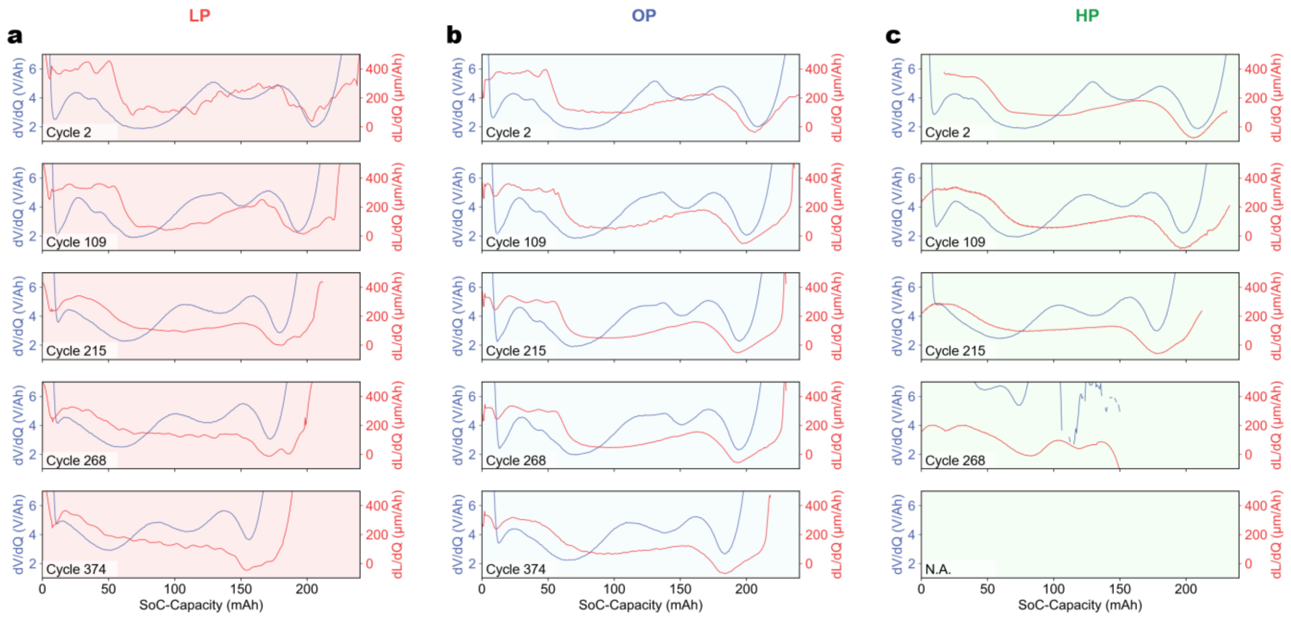

Supplementary Fig. 16 | Additional differential cell thickness expansion (dL/dQ) and differential voltage (dV/dQ) curves: Under (a) LP, (b) OP and (c) HP conditions, plotted against capacity during charging for cycles 2, 109, 215, 268 and 374.

Step dL/dQ	Step dV/dQ	Process
1	a	Gr (C_6) → 1L – 4L (LiC_{72})
2		Gr 1L – 4L (LiC_{72})
	b	Gr 4L (LiC_{36})
3		Gr 1L – 4L (LiC_{72}) → 4L (LiC_{36}) → 3L (LiC_{27})
	c	Gr 4L (LiC_{36}) → 3L (LiC_{27})
4	d	Gr 3L (LiC_{27})
5		Gr 3L (LiC_{27}) → 2L (LiC_{18})
6	e	Gr 2L (LiC_{18})
7	f	Gr 2L (LiC_{18}) → 2 (LiC_{12})
	g	NMC M (by analogy to $LiNiO_2$)
8	h	Gr 2 (LiC_{12}) [after this: 2(LiC_{12}) → 1(LiC_6)]
	i	NMC M → H2 (by analogy to $LiNiO_2$)
9	j	NMC H2 (by analogy to $LiNiO_2$)
10	k	NMC H2 → H3 (by analogy to $LiNiO_2$)

Supplementary Table. 3 | Summary of phases and phase transitions captured by differential curves.[27,28]

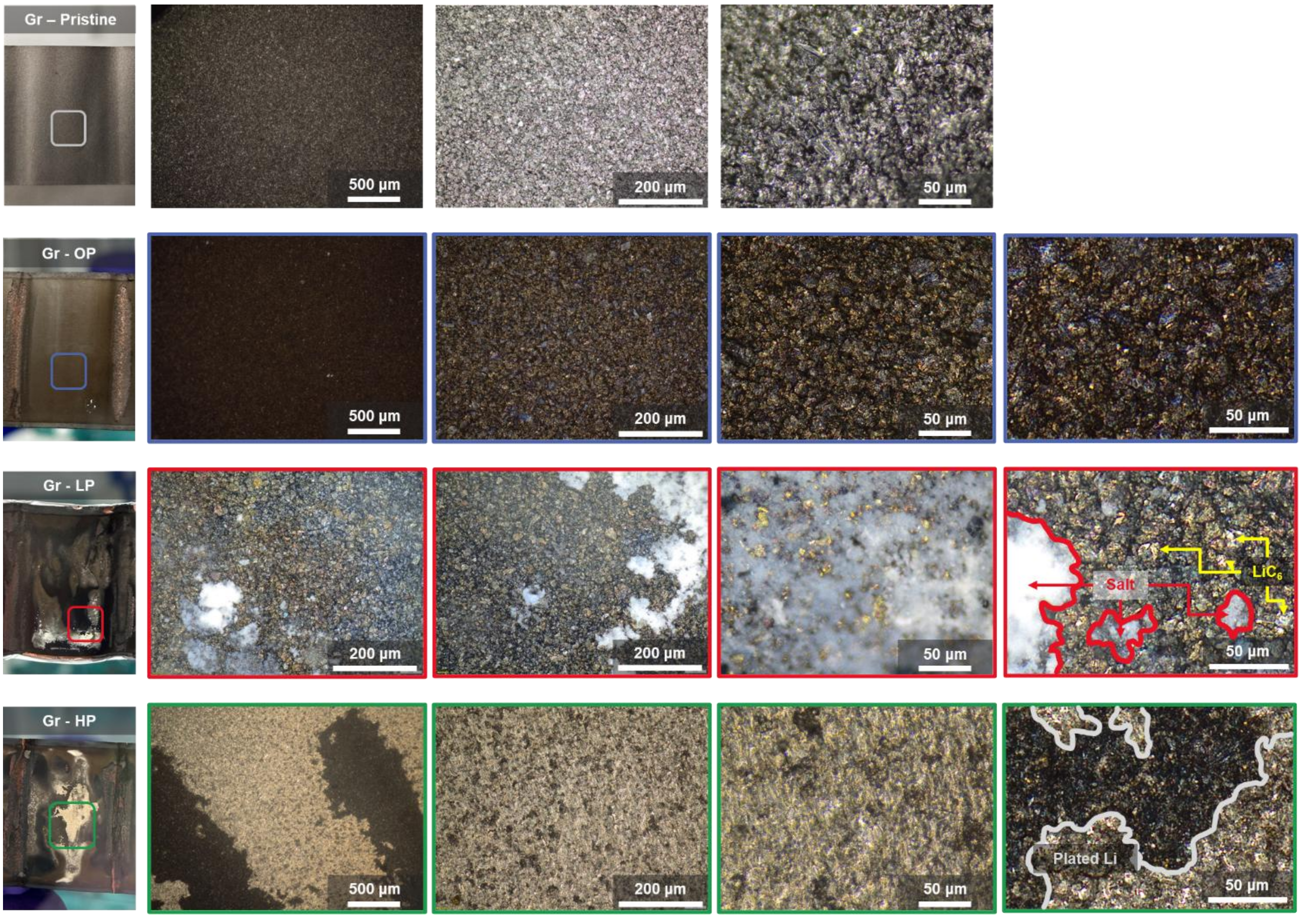

Supplementary Fig. 17 | Additional anode optical microscope images: Pristine, OP, LP and HP graphite anodes (from top to bottom) after 268 cycles.

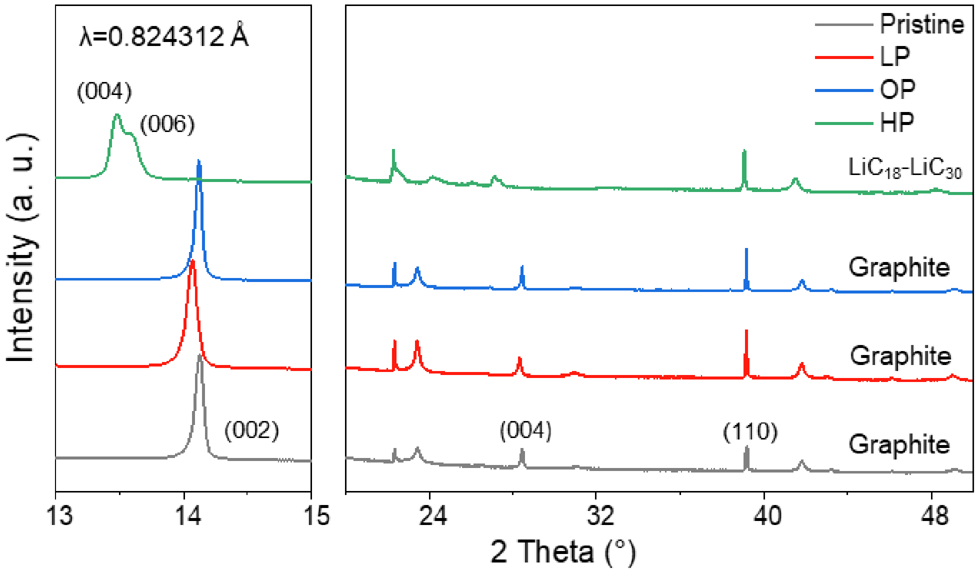

Supplementary Fig. 18 | Post-mortem sXRD patterns of pristine and cycled graphite anodes under LP, OP, and HP conditions after 268 cycles: The sXRD experiments were conducted at Beamline I11 of the Diamond Light Source, UK, using an X-ray wavelength of 0.82686 Å.

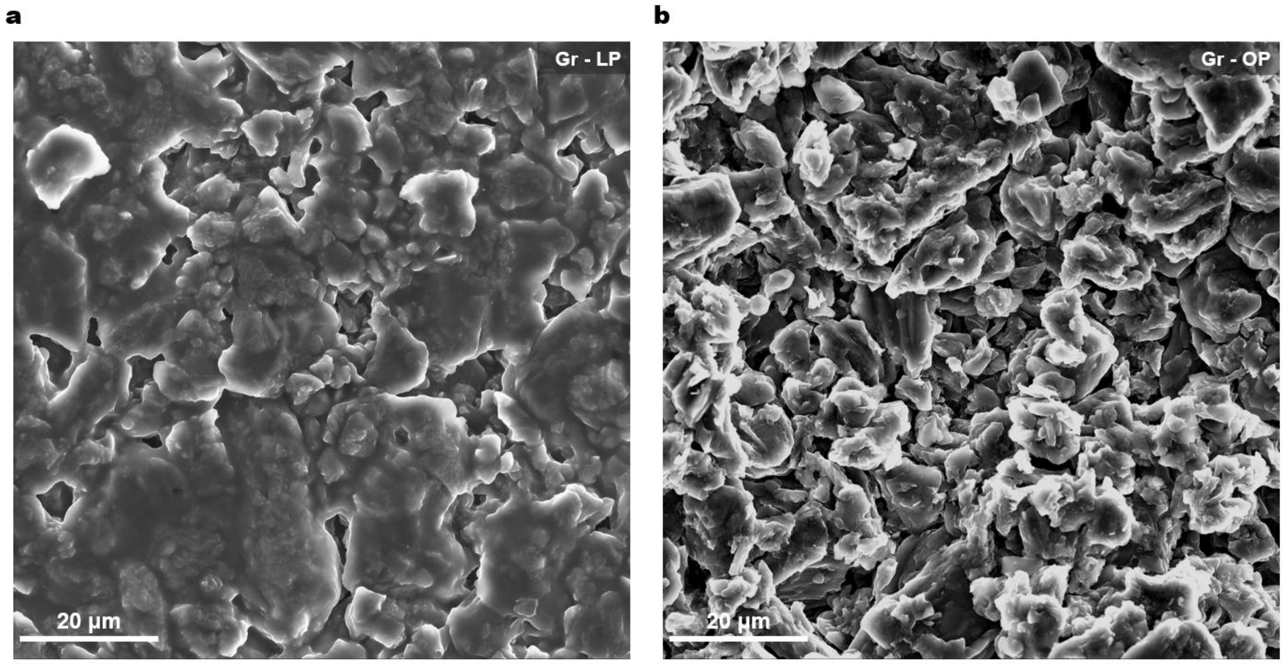

Supplementary Fig. 19 | Post-mortem SEM images of graphite anode surface morphology after 374 cycles under (a) LP and (b) OP conditions. The pronounced morphological differences indicate significantly greater electrolyte decomposition on the LP anode surface.

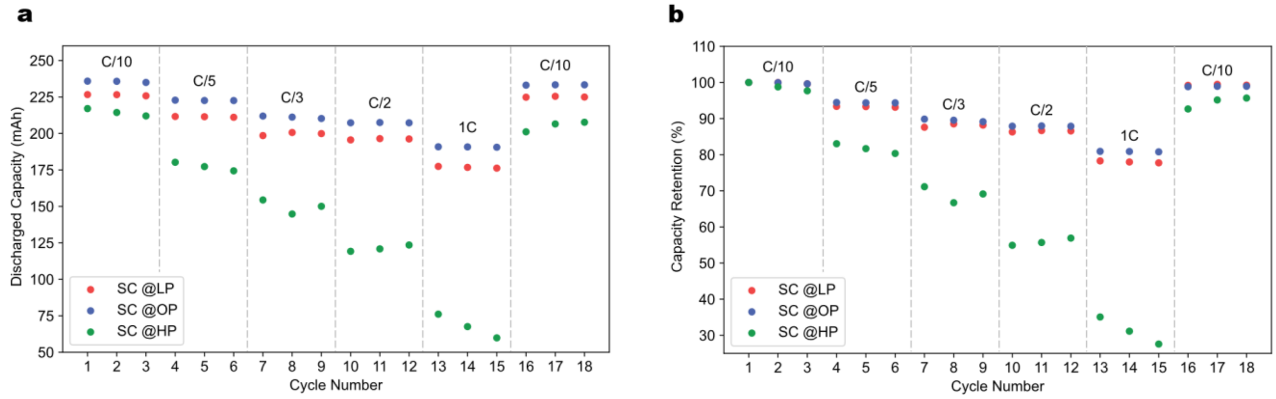

Supplementary Fig. 20 | Rate capability test performance under LP (109 cycles), OP (109 cycles), and HP conditions (162 cycles) of the cycled NMC811/Graphite cells: (a) Discharge capacity at C/10, C/5, C/3, C/2, 1C. (b) Capacity retention, with HP showing the lowest retention at 1C.

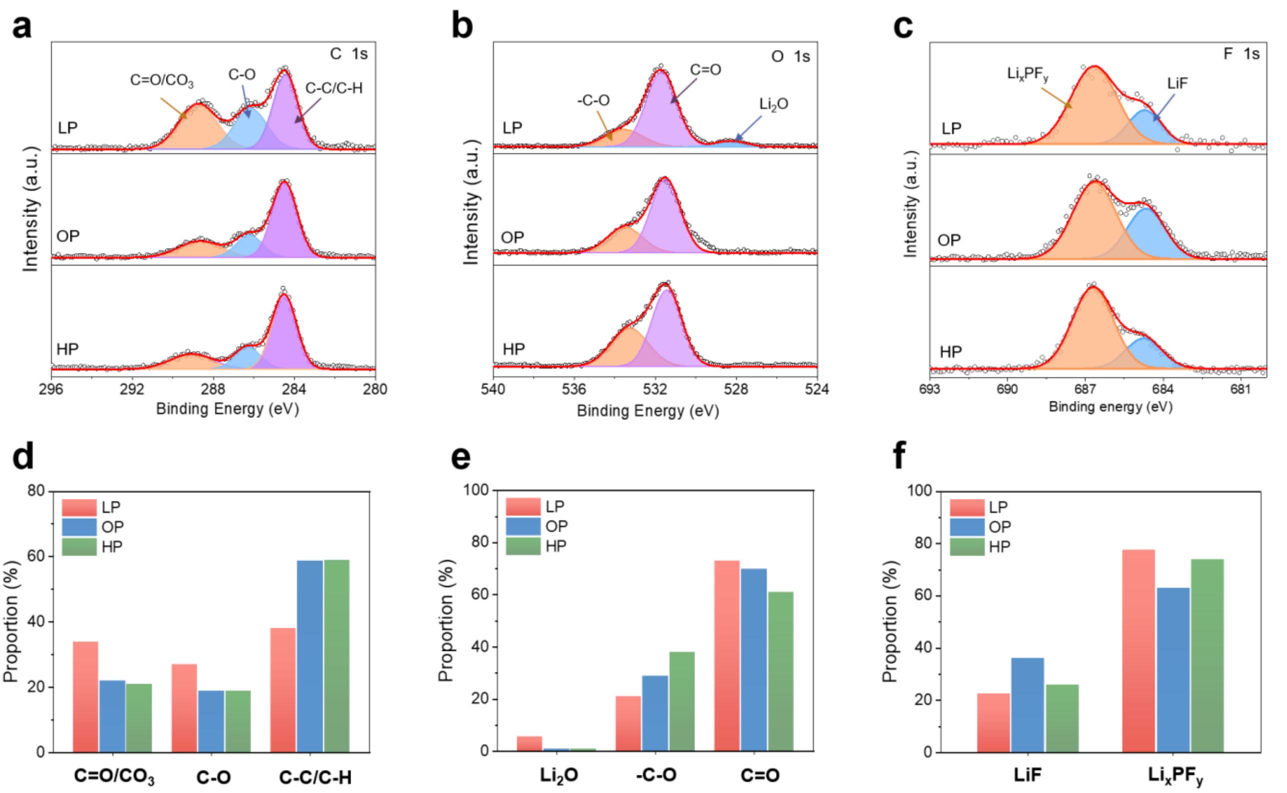

Supplementary Fig. 21 | Fittings and proportion of the deconvoluted components of XPS spectra of graphite anodes after 268 cycles: (a,d) C 1s spectra, (b,e) O 1s spectra, (c,f) F 1s spectra under LP, OP, and HP conditions.

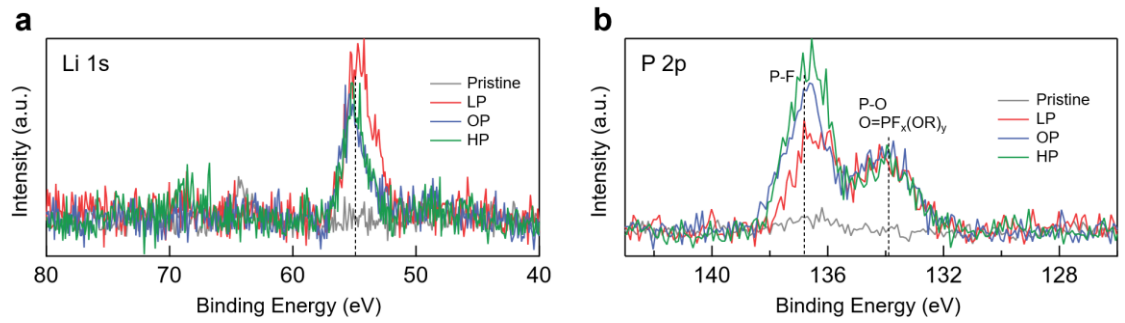

Supplementary Fig. 22 |Additional XPS spectra of graphite anodes after 268 cycles: (a) Li 1s spectra, (b) P 2p spectra under LP, OP, and HP conditions. The presence of more salt decomposition taking place on higher stack pressure electrodes is also seen from the higher intensity of the P 2p spectra, supporting the observations of the F 1s spectra. The XPS confirms that optimal stack pressure condition (OP) promotes the formation of a more stable LiF-rich SEI, which contains fewer organic species.

Supplementary Note. 5 | Analysis of Hysteresis in Anode Dilation

To better quantify the difference in anode expansion and contraction, a more detailed breakdown of the expansion data from the dilatometer is conducted. Supplementary Fig. 23a shows the asymmetry in the expansion and contraction curves. This hysteresis in expansion is particularly noticeable between 25-80% SOC, with the electrode retaining a higher thickness, during discharge. The greater difference happens around 60 mAh, which is a standard anodic feature mainly contributed from Stage 2L-2 transition of graphite.[29]

The dilation profiles of NMC811-Graphite full cells have been well-reported and studied by various groups in the past.[27,29] Between 0 to 60 mAh, the majority of the expansion is dominated by the graphite anode liquid-like stages, from dilute phase 1 (1L) to 4L, 3L and 2L liquid-like stages. The cells expand at a slower rate during charging than they contract during discharging during these stages (thickness change captured in Supplementary Fig. 23c), resulting in the expansion hysteresis observed. This microstructural change is likely partly correlated with porosity variation in the anodes,[2] as illustrated in the schematic of Supplementary Fig. 23b. The hysteresis value (Supplementary Fig. 23d) can be estimated using the equation below. This hysteresis can be viewed as the "pore breathing", indicating the change of porosity in the electrodes:

$$\Delta L_{pore} = \Delta L_{Gr,D} - \Delta L_{Gr,C}$$

During the early cycles, the lower the stack pressure, the higher the anodic expansion (Supplementary Fig. 23c) as well as the "pore breathing" (Supplementary Fig. 23d) This greater anode expansion and contraction in LP cells could cause damage to the SEI and contribute to more fresh graphite surfaces exposed to the electrolyte repeatedly every cycle.

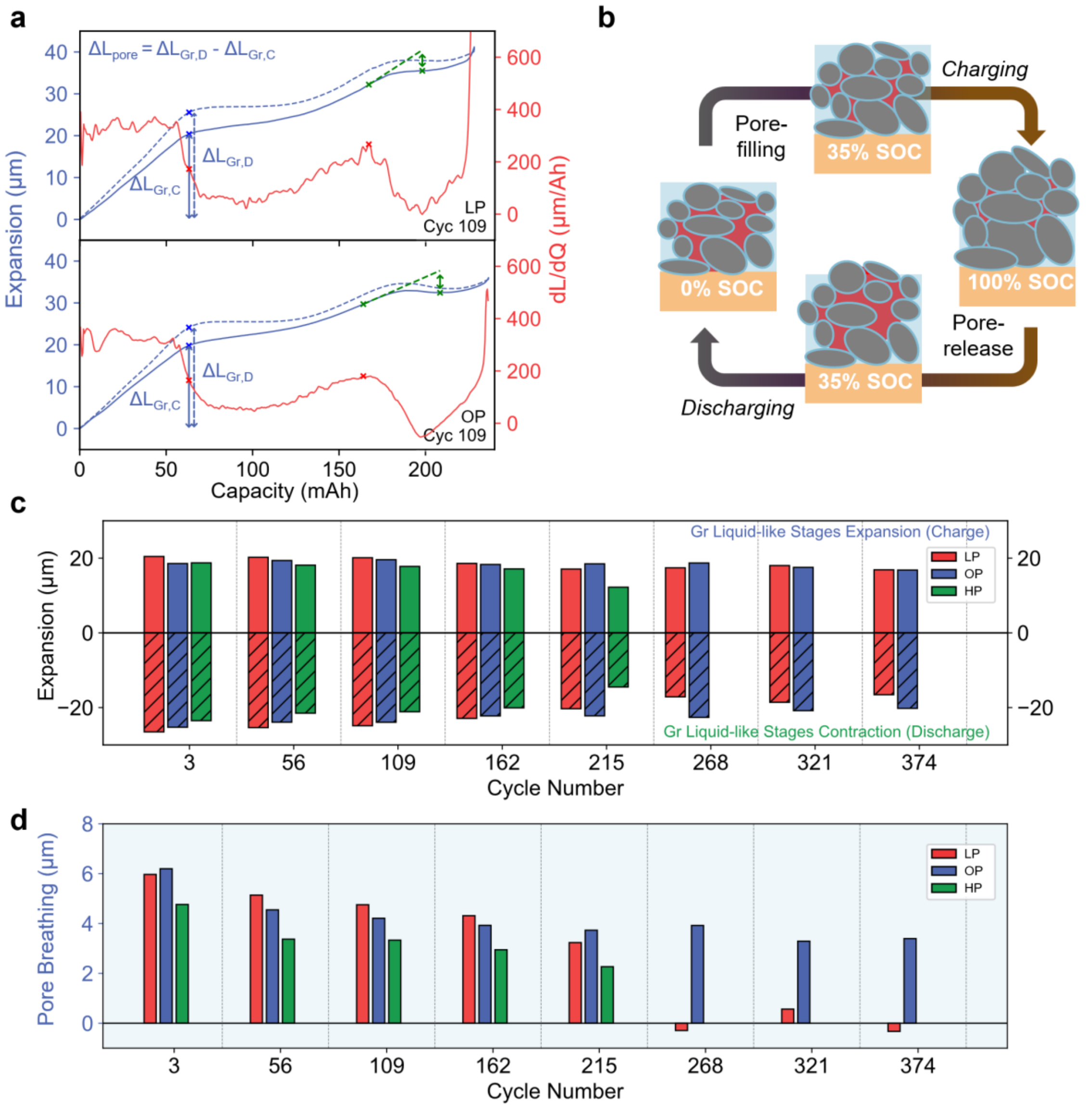

Supplementary Fig. 23 | Analysis of hysteresis in anode dilation: (a) Representative cycles from LP and OP conditions, illustrating how specific anode dilation features can be extracted. (b) Schematic of the “pore breathing” process (responsible for expansion hysteresis) in the anodes. (c) Graphite expansion and contraction during the liquid-like phases. (d) Cell thickness changes associated with porosity variations (“pore breathing”) during a full charge-discharge cycle under LP, OP, and HP conditions.

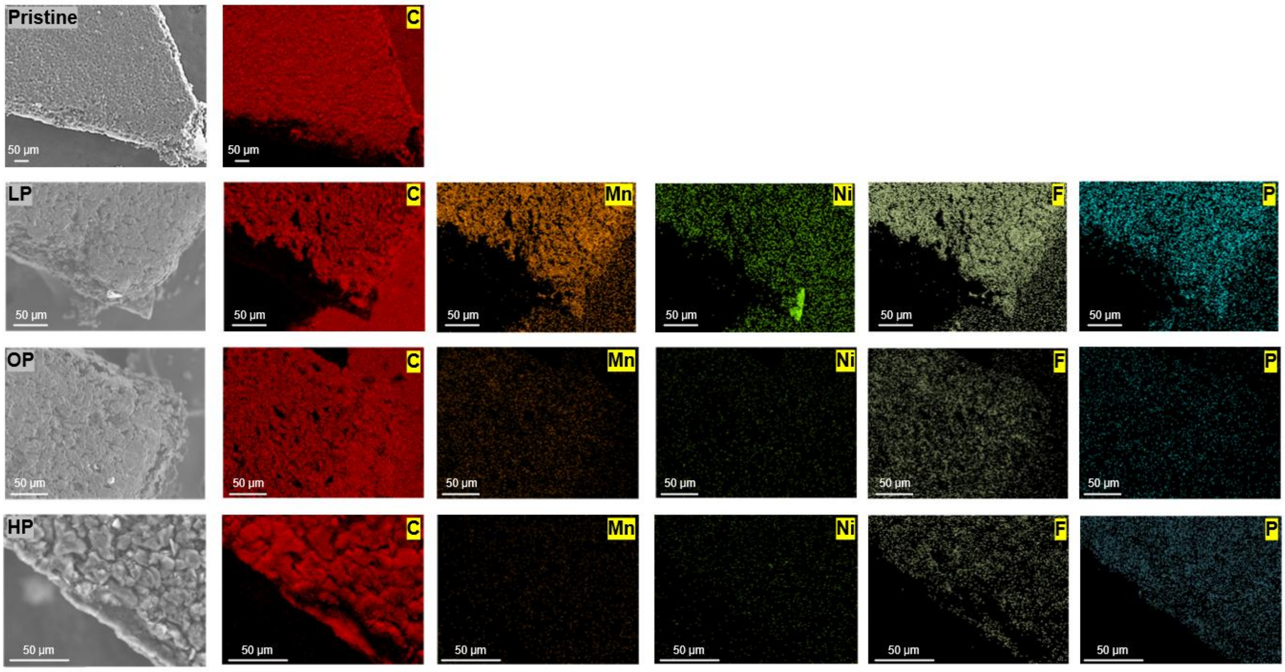

Supplementary Fig. 24 | SEM-EDX Analysis of graphite anode samples: (a)Pristine, (b)LP after 268 cycles, (c)OP after 268 cycles, (d)HP after 268 cycles. Surface morphology was observed by SEM (Zeiss Gemini FEG), and elemental mapping of C, Mn, Ni, F, and P was performed using an EDX detector (Oxford Instruments) to evaluate surface composition and distribution after cycling. Scale bars: 50 µm.

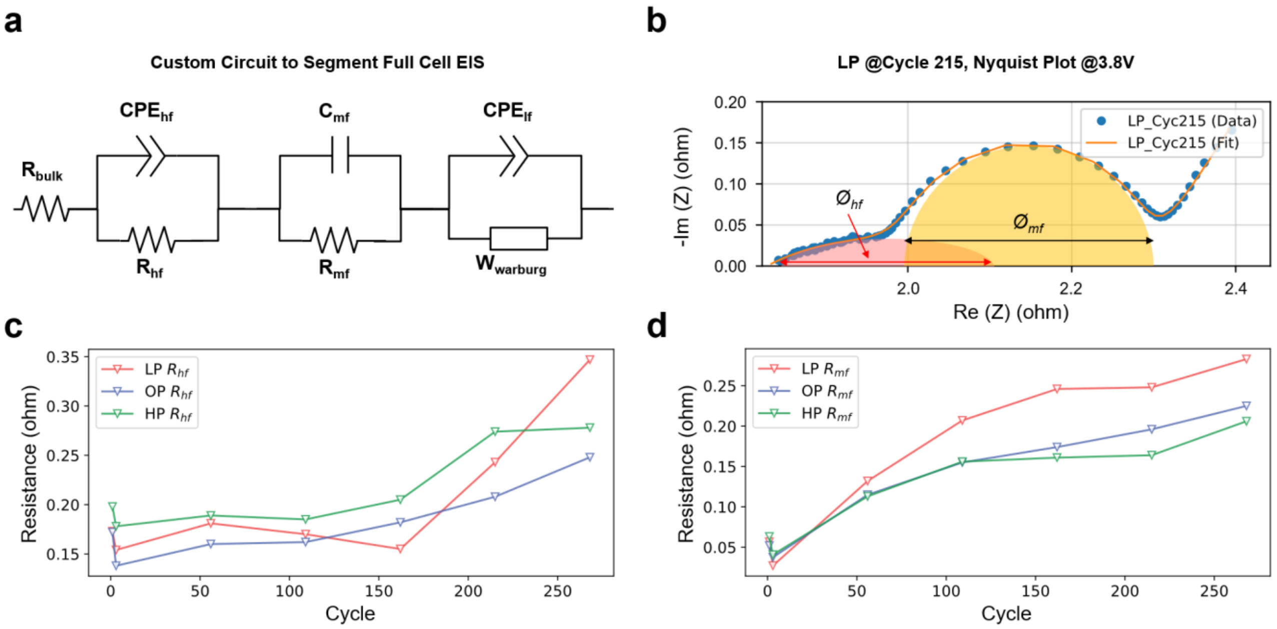

Supplementary Fig. 25 | EIS fitting results for LP, OP, and HP cells during long-term cycling: (a) Equivalent circuit model used to fit the full-cell EIS data, comprising elements representing bulk resistance (R_{bulk}), high-frequency resistance (R_{hf}), mid-frequency resistance (R_{mf}), and Warburg diffusion element. (b) Representative Nyquist plot of an LP cell at Cycle 215 measured at 3.8 V, showing the fitting of the experimental data (blue dots) using the circuit model (orange curve). The high-frequency ($Ø_{hf}$) and mid-frequency ($Ø_{mf}$) semicircles are annotated. (c) Evolution of fitted high-frequency resistance and (d) mid-frequency resistance over cycling for LP, OP, and HP cells. OP and HP show slower resistance growth compared to lower pressure (LP), particularly in the mid-frequency region associated with charge-transfer resistance likely related to the cathode.[26,30,31] EIS spectra were fitted using the Python software package impedance.py.[32]

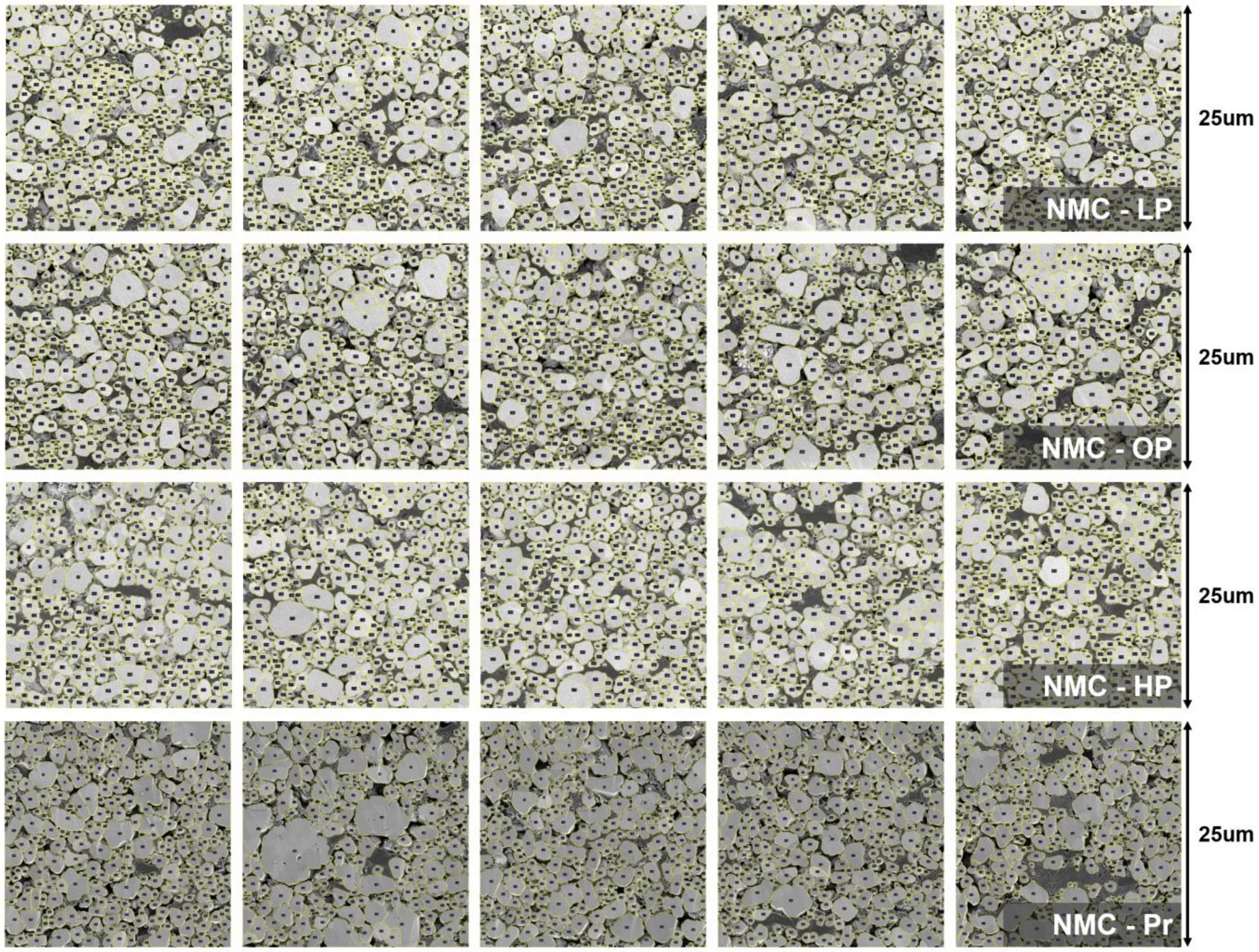

Supplementary Fig. 26 | Cross-sectional SEM of cathode samples with segmented particle boundaries: From top to bottom: LP cathodes after 374 cycles, OP cathodes after 374 cycles, HP cathodes after 268 cycles and Pristine cathodes. Particle boundaries are extracted and analysed using image J and segmented with Cellpose3 package[33] (predicted using cyto3 model with flow threshold of 0.9 and cellprob threshold of 0.1).

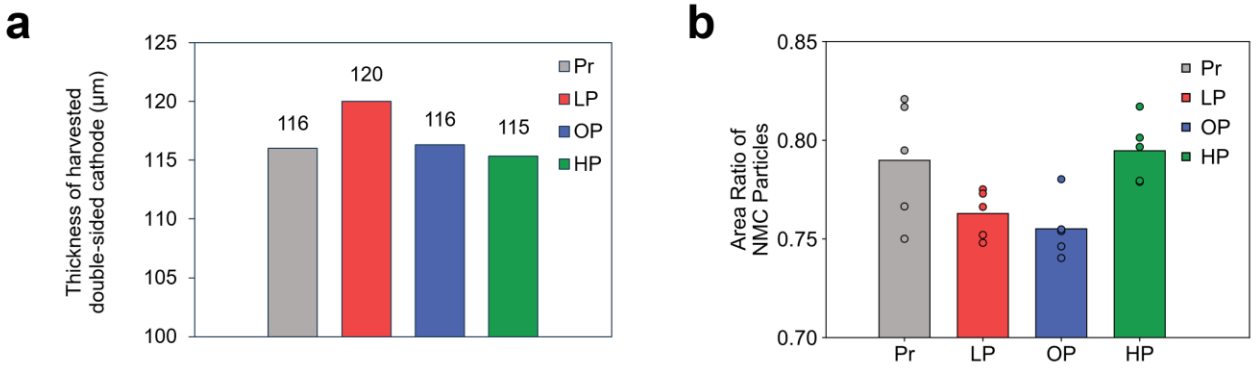

Supplementary Fig. 27| Post-mortem cathode thickness and density analysis: (a) average results from micrometre screw gauge measurement of pristine, HP (268 cycles), OP (268 cycles), LP cathodes (268 cycles). The aluminium current collector has a thickness of 12 µm. (b) Area ratio of cathode particles (corresponding to the packing density) calculated from the cross-sectional SEM under pristine, HP (268 cycles), OP (374 cycles), LP (374 cycles) conditions. Bars represent mean values calculated from the individual area ratios of each SEM image from Supplementary Fig. 26. The results highlight that the HP cathodes are likely the least porous (indicated by the lowest thickness and higher particle packing density.

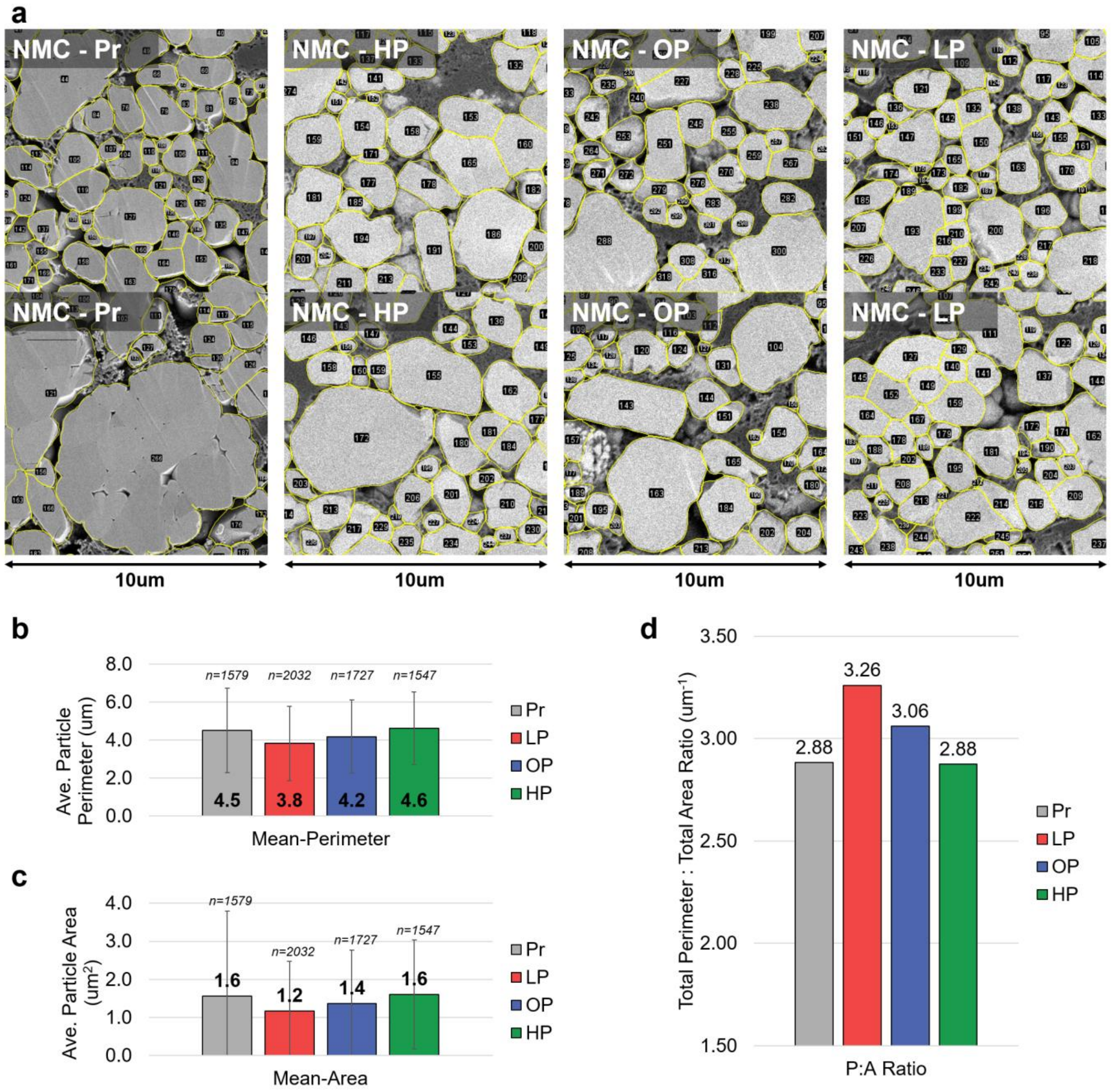

Supplementary Fig. 28| Cathode particle grain boundary separation analysis: (a) Representative 10um*10um cross-sectional SEM images of pristine, HP (268 cycles), OP (374 cycles), LP cathodes(374 cycles) from left to right, showing higher pressure better protected single crystal NMC particles. (b) Average particle perimeter with error bars representing the standard deviation (n indicated), (c) average particle area with error bars representing the standard deviation (n indicated) and (d) ratio of sum of particle perimeter to sum of particle area (P:A) in pristine, HP (268 cycles), OP (374 cycles), LP cathodes(374 cycles). The ratio indicates the degree of grain separation within the cathode structure after cycling. The results suggest that higher stack pressure reduces grain separation in the cathode particles. All particles extracted from Supplementary Fig. 26. Particle boundaries are extracted using image J and segmented with Cellpose3 package[33] (predicted using cyto3 model with flow threshold of 0.9 and cellprob threshold of 0.1).

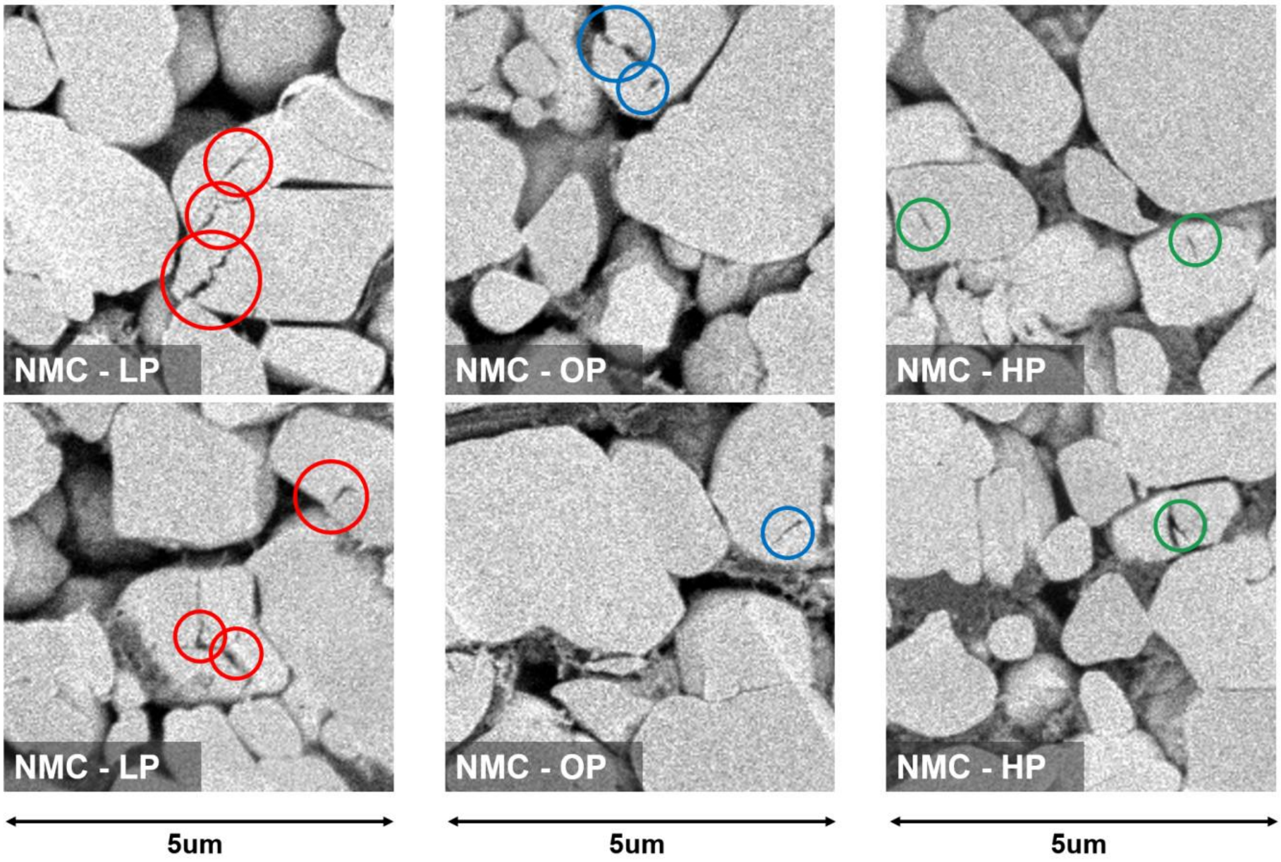

Supplementary Fig. 29| Additional cross-sectional SEM images highlighting cathode particle cracking: Representative 5um*5um cross-sectional SEM images of LP cathodes(374 cycles), OP (374 cycles), and HP (268 cycles) from left to right.

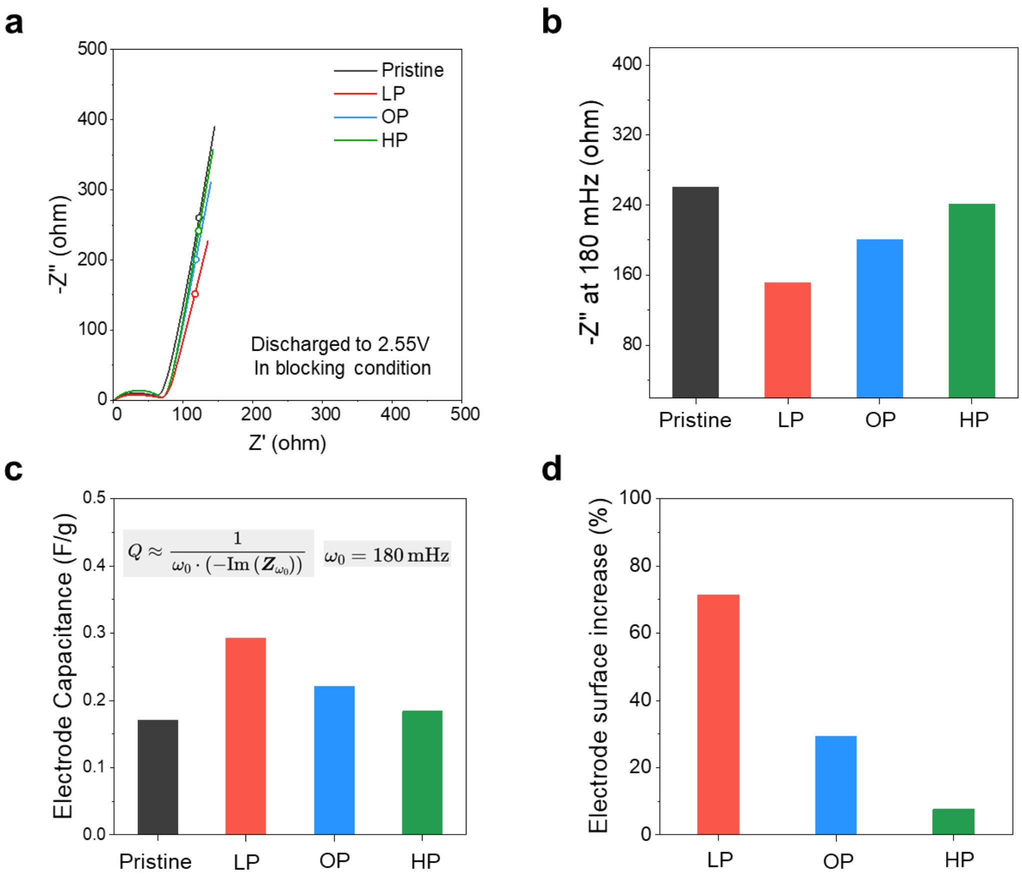

Supplementary Fig. 30 | Surface area estimation of pristine and cycled cathode samples using impedance methods[34]: (a) Nyquist plots recorded in blocking conditions at 2.55V for Pristine, LP (268 cycles), OP (268 cycles) and HP (268 cycles) electrodes. (b) The value of the negative imaginary (-Z”) contribution to the 180 mHz point in the Nyquist plots for Pristine, LP, OP and HP electrodes. (c) Electrode capacitance for Pristine, LP (268 cycles), OP (268 cycles) and HP (268 cycles) electrodes in blocking conditions. (d) The relative increase in electrode surface area for LP (268 cycles), OP (268 cycles) and HP (268 cycles) electrodes compared to the pristine electrode, calculated from capacitance measurements. To conduct this experiment, pristine electrodes and cycled electrodes were harvested from the pouch cells. These electrodes were assembled with fresh lithium chips into half-cells, which were then subjected to constant-voltage discharge at 2.55 V to ensure both electrodes reached a block condition. The capacitance values were measured at a frequency of 180 mHz, directly correlating to the specific surface area of the electrodes.[34] By comparing the capacitance values of the pristine, LP, OP, and HP electrodes, the increase in surface area for the cycled electrodes was determined. The results show the greatest increase in the electrode's specific surface area after cycling under low pressure.

Supplementary Note. 6 | A Mechanistic Explanation for Crack Initiation and Propagation in Cathode

To understand why cathodes crack more under low pressure and why more cracks are between 40° and 50° after cycling, we first consider the force chain theory developed for granular materials.[35,36] This theory suggests that in granular media under compression, the force is transmitted through chains of particles that are touching each other, forming load-bearing chains through the thickness of the electrode. The NMC particles in the cathode can be considered as a granular material. Applying external pressure to the pouch does not result in a uniform stress distribution within the cathode. Instead, the stress is transmitted through force chains,[35] which form preferred pathways for load transfer (Supplementary Fig. 31a).

Since NMC particles are embedded in a liquid electrolyte, the effective stress acting on the cathode is given by the equation $p_{eff} = p_{applied} - p_{fluid}$, where $p_{applied}$ is the externally applied stack pressure and p_{fluid} is the pressure due to the electrolyte. For simplicity, we take $p_{eff} \approx p_{applied}$. In our cathode, NMC particles are packed in a granular structure, following Hertzian contact mechanics at the particle level.[37] At the contact point between these particles, local stress concentration (Hertzian contact, P_{Hertz}) is likely to initiate cracks. The Hertzian pressure at the contact point is given by:

$$P_{Hertz} = \frac{(3FE^2)^{\frac{1}{3}}}{2\pi(1-\nu^2)^{\frac{2}{3}}}, \qquad (1)$$

where E and ν are modulus and Poison ratio of a single NMC particle. F is the applied contact force. Eq (1) above shows that the local pressure at each contact depends on the applied force and material properties of the particles.

To understand the contact force F, it is important to consider how stress is transmitted at the bulk scale. The force distribution in NMC particles is not uniform, as force chains form preferred pathways through the granular network. This leads to localised stress concentrations, which strongly depend on the coordination number, Z, the average number of contacts per particle. The contact force, F, is related to the coordination number by the equation $F \propto (V_p/Z)$, where V_p is the average volume of a single NMC particle. Substituting in (1), we get the relationship between Hertzian contact and coordination number:[35,37–39]

$$P_{Hertz} \propto (V_p/Z)^{\frac{1}{3}} \propto Z^{-\frac{1}{3}} \qquad (2)$$

The coordination number plays a key role in stress distribution, follows a logarithmic increase, and can be empirically written as:[38,39]

$$Z = Z_0 + c \cdot \log(p_{applied}) \qquad (3)$$

Z_0 is the coordination number at zero pressure, c is a material constant of the NMC particles. $p_{applied}$ is the externally applied stack pressure as previously mentioned. The equation suggests that the coordination number would increase rapidly at low pressures and saturate slowly at higher pressures. This suggests that as external applied stack pressure increases, more contacts form between particles, the coordination number increases, and consequently the Hertzian pressure decreases, meaning that stress is distributed over more contacts. Supplementary Fig. 31b is a plot to show the qualitative variation of contact pressure P_{Hertz} with the applied pressure $p_{applied}$ based on equations (2) and (3) and an arbitrary choice of parameters. The plot is intended to illustrate the proposed behaviour qualitatively: it should not be used for comparison with experiments, as that would require more accurate estimation of various parameters in equations (1), (2) and (3), which is beyond the scope of the present study. For this purpose, we have normalised both axes by an average strength of NMC particles as ~200 MPa, while assuming $Z_0 = 4$ and $c = 0.06$.

In our study, at low pressure (LP), the coordination number is likely small, and therefore the Hertzian pressure is higher. This means that a small fraction of particles carries most of the load, leading to higher stress concentrations and an increased likelihood of crack initiation. In contrast, at higher pressure (OP or HP conditions), force chains become more interconnected, allowing more particles to share the load, reducing localised stress and lowering the likelihood of fracture. Thus, higher compression leads to better stress distribution, reducing the risk of crack formation, whereas low compression results in stress hotspots, leading to faster crack initiation.

To explain the observed 40 - 50° crack propagation in NMC cathodes, we propose the following granular-scale stress transmission mechanism. The stress tensor in the cathode is given by:

$$\sigma_{ij} = \frac{1}{V}\sum_{contacts} F_i \ell_j \qquad (4)$$

where V is the volume of the granular medium, F_i is the contact force at a particle and ℓ_j is the branch vector between particle centres. Empirical studies and simulations suggest that branch vector length scales with coordination number,[40] $\ell_{avg} \propto Z^{-\frac{1}{3}}$, for a simple monodisperse granular network. This means that as stack pressure increases, the coordination number, Z increases, leading to shorter branch vectors. A higher coordination number results in a more interconnected granular network, reducing stress localisation. However, under low pressure, the coordination number Z is low, the branch vector length ℓ_j increases, which in turn amplifies deviatoric stress components, leading to higher shear stresses.

In addition, due to the irregular shape of NMC particles, forces are not always applied directly between particle centres. Instead, most forces act at an angle, introducing large shear stresses. The stress tensor component responsible for shear stress is, $\sigma_{xy} = \frac{1}{V}\sum_{contacts} F_x \ell_y$. The deviatoric stress tensor, which influences shear-induced cracking, is given by:

$$s_{ij} = \sigma_{ij} - \frac{1}{3}\sigma_{kk}\delta_{ij} \qquad (5)$$

The maximum shear stress is $\tau_{max} = \frac{1}{2}(\sigma_1 - \sigma_3)$, where σ_1 and σ_1 are the principal stresses. Cracks therefore tend to form at around 45° to the maximum compressive stress when the shear stress is high. At low pressure, the deviatoric stress $(\sigma_1 - \sigma_3)$ is high, leading to higher shear stress. This increases the likelihood of cracks forming at 40 - 50°. Since stress transmission is less uniform, localised fractures occur more frequently, weakening the cathode structure due to fatigue loading (charge/discharge cycles). High pressure suppresses deviatoric stress, resulting in better connectivity between particles, which stabilises the structure, delaying crack propagation and improving the mechanical stability of the cathode.

When applying classic granular medium theory to battery electrodes, it is important to keep in mind that, unlike in the case of passive materials, the mechanical properties of the battery particles change both as a function of SOC and state of health. In particular, in this work, we charge our batteries to a high SOC, which means lower yield shear strength at the top of charge. Second, as discussed above, the cathodes form a surface reduced layer over time, which may, for instance, increase the chances of crack initiation. Finally, we observed a decrease in electrode thickness over time, which suggests that as particles swell and shrink during the cycle, they reorganise, which can lead to changes in force chains over time. These effects can lead to deviations from classic granular media mechanical theory.

Note that the above analysis does not directly apply to crack formation during the calendering stage, as both crack initiation and crack propagation follow fundamentally different mechanisms under constant load (one-off calendering) compared to cyclic load (charge-discharge testing). Under cyclic loading, cracks typically initiate through the accumulation of micro-damage over repeated cycles, and can form even below the material's yield stress (often referred to as "fatigue"). In contrast, under constant loading, crack initiation generally occurs only when the applied stress exceeds the yield strength. Second, calendering is performed on discharged electrodes, where the cathode is mechanically strongest. During cycling, however—particularly at the top of charge—the Single Crystal $Li_yNi_{0.8}Mn_{0.1}Co_{0.1}O_2$ cathode exhibits a 55% reduction in shear strength (from 86 ± 12 MPa at y = 1 to 39 ± 5 MPa at y = 0.14 as reported in our previous work[19]). In this work, pushing the single crystal layered oxide cathode to high UCV, combined with the higher deviatoric stress under low external compression, contributes to a higher likelihood of crack initiation and propagation. Third, the mechanisms governing crack growth also differ: cyclic loading is often described by Paris' Law, while constant loading is governed by creep growth laws, which are time-dependent.[41,42] Given the short duration of pressure application during calendering, it is not surprising that cathodes subjected to a brief, high-pressure calendering step exhibit fewer cracks than those exposed to prolonged low-pressure cycling over months—especially as the material becomes mechanically weakened each time it reaches a high SOC.

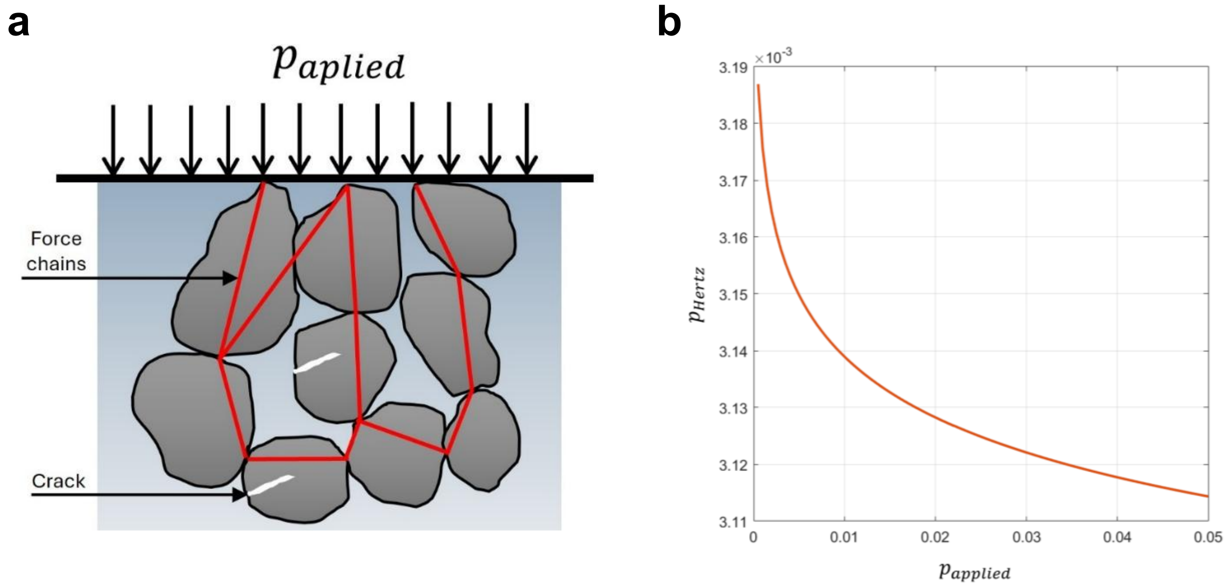

Supplementary Fig. 31 | Crack initiation and propagation mechanism: (a) Schematic of the NMC particles and the formation of force chains under external pressure. (b) A trend line for showing the variation of local contact pressure with applied pressure (both normalised by the average strength of NMC particles taken as ~200 MPa). $\boldsymbol{P_{Hertz}} \propto \boldsymbol{Z}^{-\frac{1}{3}}$, where $\boldsymbol{Z} = \boldsymbol{Z_0} + \mathbf{0.06}\,\mathbf{log}\,(\boldsymbol{p_{applied}})$. This suggests that the local contact pressure initially decreases sharply as the applied pressure rises from low levels and becomes largely insensitive to further increases in applied pressure.

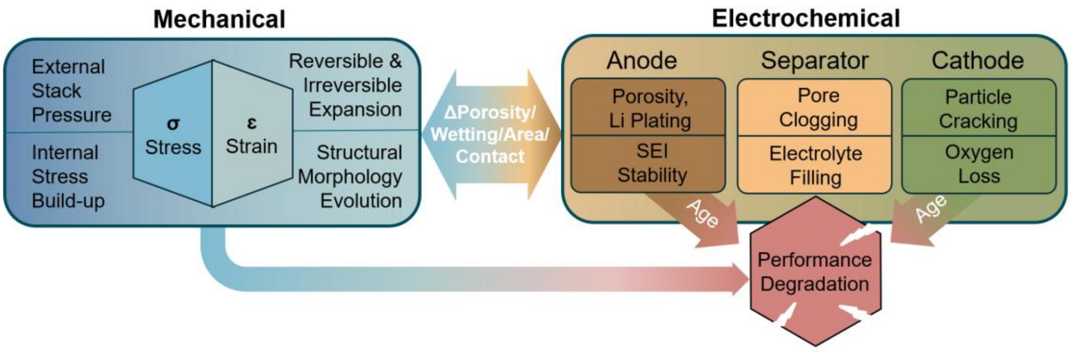

Supplementary Fig. 32 | Relationship between mechanical and electrochemical parameters. The diagram highlights the relations between mechanical stress and strain (Left) and the electrochemical performance and ageing of the battery anode, cathode and separator (right).

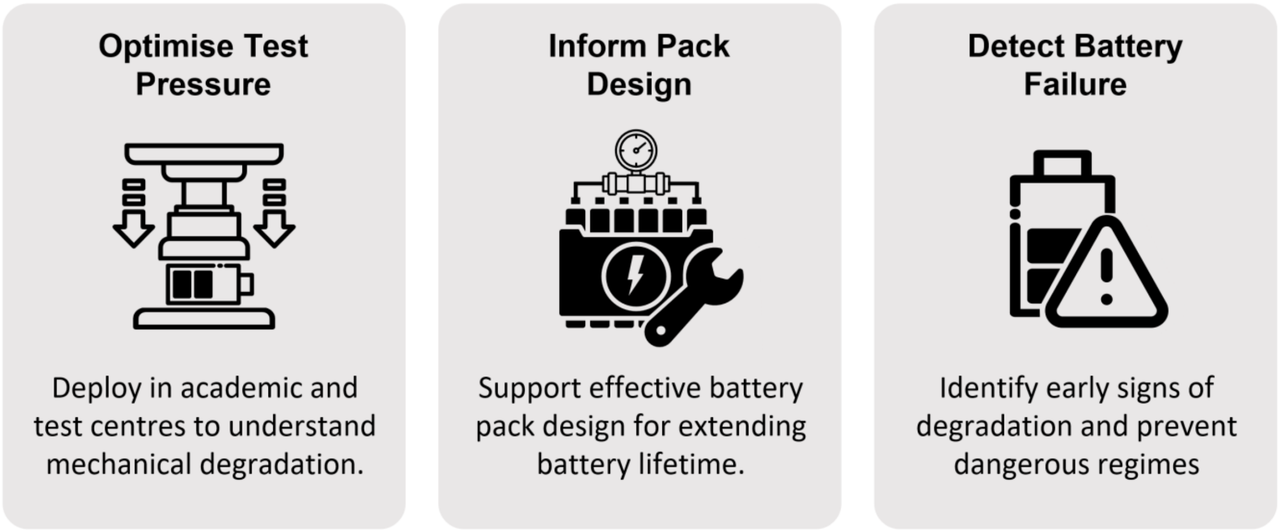

Supplementary Fig. 33 | Pictograms illustrating the key applications of our tool, including optimisation of stack pressure, pack design and identification of cell failure signals.

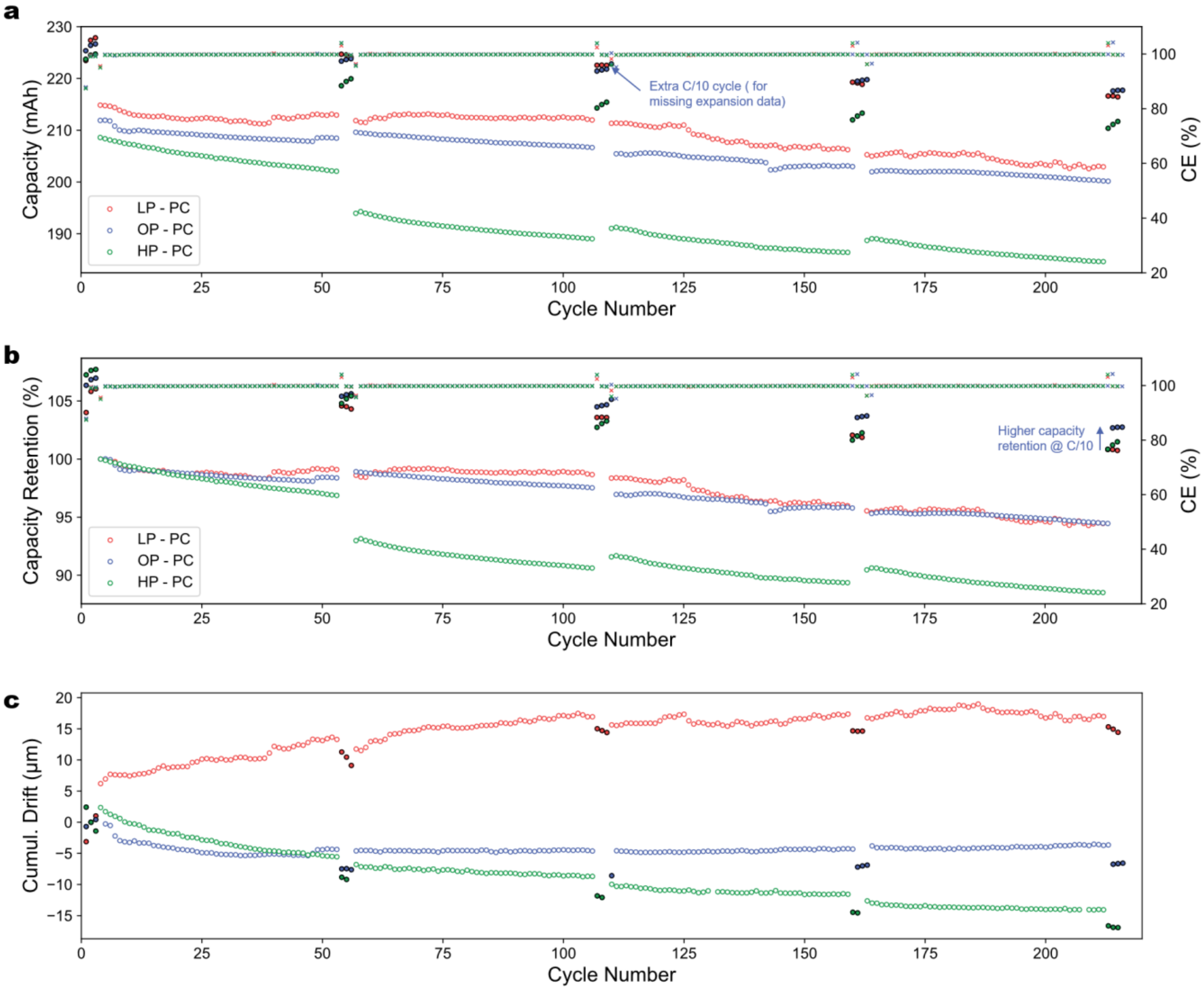

Supplementary Fig. 34 | Long-term cycling performance of PC-NMC811/graphite pouch cells under LP, OP and HP. The cells were cycled at a C-rate of C/3 (70 mA) in a voltage window of 2.8 - 4.3 V, with slow C/10 (21 mA) cycles during formation and recovery, indicated in solid points. (a) Discharge capacity and coulombic efficiency (CE). (b) Capacity retention, with OP showing the highest retention at C/10 after 215 cycles. (c) Cumulative thickness drift, exhibiting a similar trend to SC cells: HP cells continue compacting, while LP cells gradually expand over time.

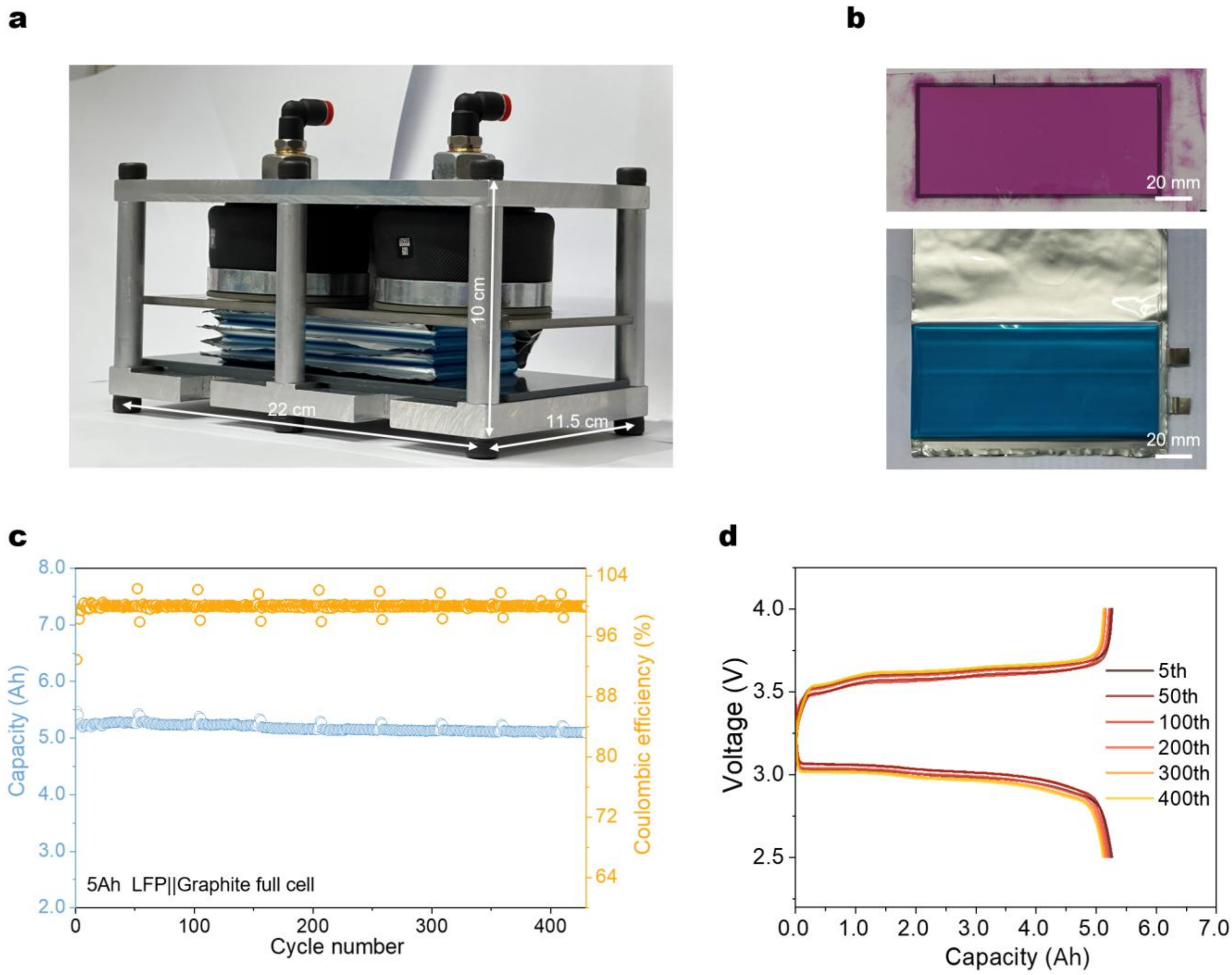

Supplementary Fig. 35 | Demonstration of scale-up module for 5-Ah LFP/graphite pouch cells. (a) The mock-up module consists of four 5-Ah LFP-Graphite pouch cells (individual cell dimensions: 125 mm × 60 mm × 6 mm). (b) Pressure paper test demonstrating homogeneous stack pressure across the pouch cell electrode area. (c) Cycling discharge capacity and (d) voltage curves at C/3, after the first two formation cycles at C/10.

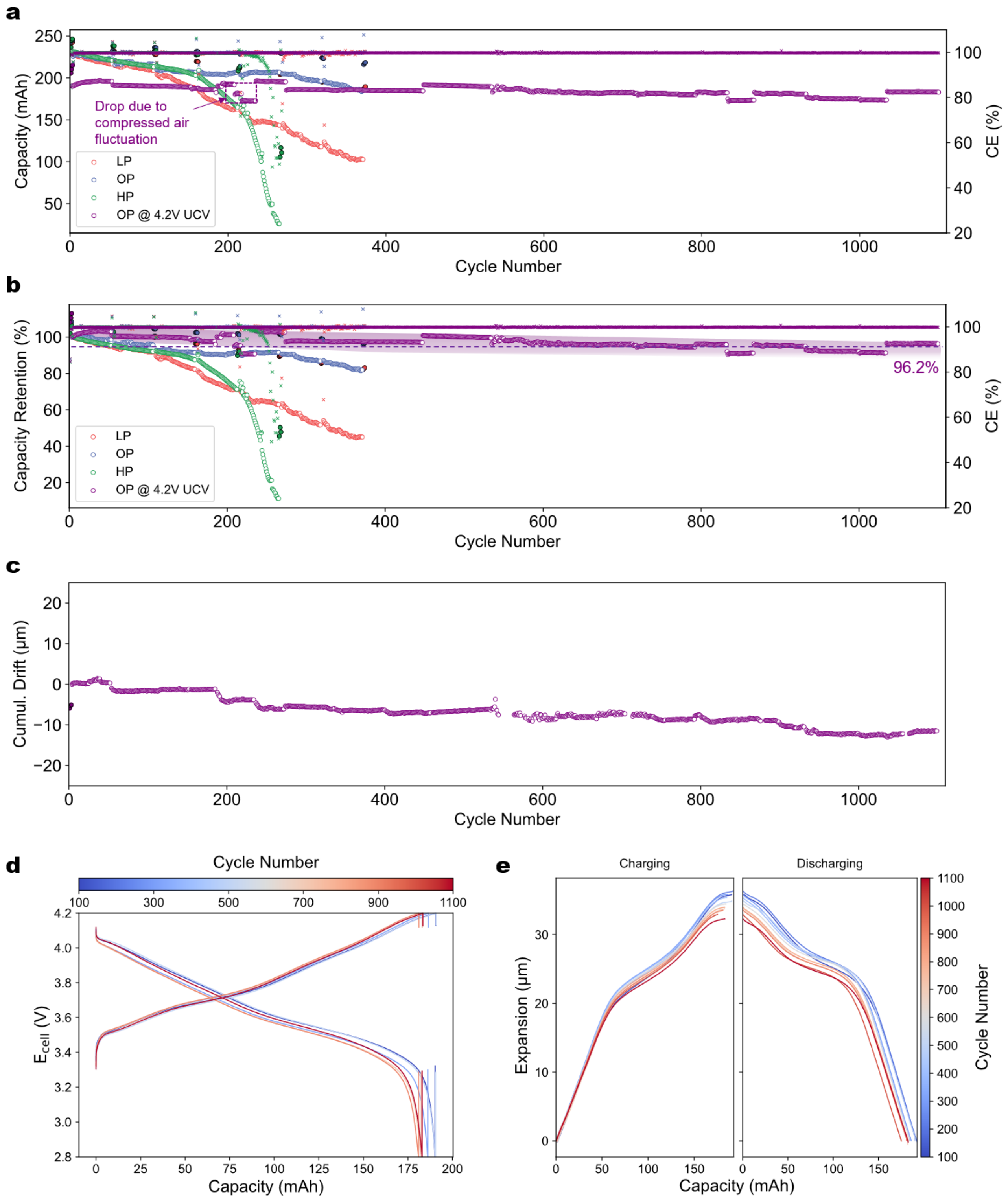

Supplementary Fig. 36 | Long-term cycling performance of OP cell at a 4.2 V UCV compared to the cells at a 4.6V UCV under LP, OP and HP. (a) Discharge capacity and coulombic efficiency (CE). (b) Capacity retention, with OP @ 4.2 V UCV achieving 96.2% after 1100 cycles. The electrolyte used for the 4.2V UCV OP cell is 1.3M $LiPF_6$ in DEC/EC/EMC (2/3/5, v/v/v) + 9.1 wt% FEC + 0.2 wt% $LiBF_4$ (lithium tetrafluoroborate) + 0.5 wt% VC. (c) Cumulative thickness drift highlighting mechanical stability and absence of irreversible cell expansion in OP at a 4.2 V UCV. (d) Representative voltage curves and (e) expansion curves of OP cell cycled to 4.2V UCV.

Supplementary Reference

1. Widyantara, R. D., Zulaikah, S., Juangsa, F. B., Budiman, B. A. & Aziz, M. Review on Battery Packing Design Strategies for Superior Thermal Management in Electric Vehicles. *Batteries* **8**, (2022).
2. Li, R. *et al.* Effect of external pressure and internal stress on battery performance and lifespan. *Energy Storage Mater.* **52**, 395–429 (2022).
3. Nadimpalli, S. P. V., Sethuraman, V. A., Abraham, D. P., Bower, A. F. & Guduru, P. R. Stress Evolution in Lithium-Ion Composite Electrodes during Electrochemical Cycling and Resulting Internal Pressures on the Cell Casing. *J. Electrochem. Soc.* **162**, A2656–A2663 (2015).
4. Chen, Z. *et al.* Detection of jelly roll pressure evolution in large-format Li-ion batteries via in situ thin film flexible pressure sensors. *J. Power Sources* **566**, 232960 (2023).
5. Leonard, A., Planden, B., Lukow, K. & Morrey, D. Investigation of constant stack pressure on lithium-ion battery performance. *J. Energy Storage* **72**, 108422 (2023).
6. Pegel, H. *et al.* Volume and thickness change of NMC811|SiOx-graphite large-format lithium-ion cells: from pouch cell to active material level. *J. Power Sources* **537**, (2022).
7. Cannarella, J. & Arnold, C. B. Stress evolution and capacity fade in constrained lithium-ion pouch cells. *J. Power Sources* **245**, 745–751 (2014).
8. Louli, A. J. *et al.* Exploring the Impact of Mechanical Pressure on the Performance of Anode-Free Lithium Metal Cells. *J. Electrochem. Soc.* **166**, A1291–A1299 (2019).
9. Martin, C., Genovese, M., Louli, A. J., Weber, R. & Dahn, J. R. Cycling Lithium Metal on Graphite to Form Hybrid Lithium-Ion/Lithium Metal Cells. *Joule* **4**, 1296–1310 (2020).
10. Hahn, S., Theil, S., Kroggel, J. & Birke, K. P. Pressure Prediction Modeling and Validation for Lithium-Ion Pouch Cells in Buffered Module Assemblies. *J. Energy Storage* **40**, 102517 (2021).
11. Zhou, L. *et al.* A study of external surface pressure effects on the properties for lithium-ion pouch cells. *Int. J. Energy Res.* **44**, 6778–6791 (2020).
12. Müller, V., Scurtu, R. G., Memm, M., Danzer, M. A. & Wohlfahrt-Mehrens, M. Study of the influence of mechanical pressure on the performance and aging of Lithium-ion battery cells. *J. Power Sources* **440**, 227148 (2019).
13. Mussa, A. S., Klett, M., Lindbergh, G. & Lindström, R. W. Effects of external pressure on the performance and ageing of single-layer lithium-ion pouch cells. *J. Power Sources* **385**, 18–26 (2018).
14. Aufschläger, A. *et al.* High precision measurement of reversible swelling and electrochemical performance of flexibly compressed 5 Ah NMC622/graphite lithium-ion pouch cells. *J. Energy Storage* **59**, 106483 (2023).
15. Cannarella, J. & Arnold, C. B. The Effects of Defects on Localized Plating in Lithium-Ion Batteries. *J. Electrochem. Soc.* **162**, A1365–A1373 (2015).
16. Zhao, Y., Spingler, F. B., Patel, Y., Offer, G. J. & Jossen, A. Localized Swelling Inhomogeneity Detection in Lithium Ion Cells Using Multi-Dimensional Laser Scanning. *J. Electrochem. Soc.* **166**, A27–A34 (2019).
17. Pressure measurement film Prescale - Fujifilm. https://www.fujifilm.com/uk/en/business/inspection/measurement-film/prescale.
18. Stallard, J. C. *et al.* Mechanical properties of cathode materials for lithium-ion batteries. *Joule* **6**, 984–1007 (2022).
19. Stallard, J. C. *et al.* Effect of Lithiation upon the Shear Strength of NMC811 Single Crystals. *J. Electrochem. Soc.* **169**, 040511 (2022).
20. Koo, J. K. *et al.* Detrimental electrochemical behavior caused by excessive high pressure on Li-ion pouch-type full cell. *Electrochem. commun.* **152**, 107518 (2023).
21. Müller, V. *et al.* Effects of Mechanical Compression on the Aging and the Expansion Behavior of Si/C-Composite|NMC811 in Different Lithium-Ion Battery Cell Formats. *J. Electrochem. Soc.* **166**, A3796–A3805 (2019).
22. Bloom, I. *et al.* Differential voltage analyses of high-power lithium-ion cells. 4. Cells containing NMC. *J. Power Sources* **195**, 877–882 (2010).
23. Bloom, I. *et al.* Differential voltage analyses of high-power, lithium-ion cells 1. Technique and application. *J. Power Sources* **139**, 295–303 (2005).
24. Bloom, I., Christophersen, J. & Gering, K. Differential voltage analyses of high-power lithium-ion cells 2. Applications. *J. Power Sources* **139**, 304–313 (2005).
25. Dose, W. M. *et al.* The influence of electrochemical cycling protocols on capacity loss in nickel-rich lithium-ion batteries. *J. Mater. Chem. A* **9**, 23582–23596 (2021).
26. Dose, W. M., Xu, C., Grey, C. P. & De Volder, M. F. L. Effect of Anode Slippage on Cathode Cutoff Potential and Degradation Mechanisms in Ni-Rich Li-Ion Batteries. *Cell Reports Phys. Sci.* **1**, 100253 (2020).

27. Laufen, H. *et al.* Correlation between Voltage, Strain, and Impedance as a Function of Pressure of a Nickel-Rich NMC Lithium-Ion Pouch Cell. *Adv. Mater. Technol.* **2301965**, 1–13 (2024).
28. Schweidler, S. *et al.* Volume Changes of Graphite Anodes Revisited: A Combined Operando X-ray Diffraction and in Situ Pressure Analysis Study. *J. Phys. Chem. C* **122**, 8829–8835 (2018).
29. Spingler, F. B., Kücher, S., Phillips, R., Moyassari, E. & Jossen, A. Electrochemically Stable In Situ Dilatometry of NMC, NCA and Graphite Electrodes for Lithium-Ion Cells Compared to XRD Measurements. *J. Electrochem. Soc.* **168**, 040515 (2021).
30. Dennis Dees, Evren Gunen, Daniel Abraham, Andrew Jansen, J. P. Alternating Current Impedance Electrochemical Modeling of Lithium-Ion Positive Electrodes. **2900**, (2005).
31. Gilbert, J. A. *et al.* Cycling Behavior of NCM523/Graphite Lithium-Ion Cells in the 3–4.4 V Range: Diagnostic Studies of Full Cells and Harvested Electrodes. *J. Electrochem. Soc.* **164**, A6054–A6065 (2017).
32. Murbach, M., Gerwe, B., Dawson-Elli, N. & Tsui, L. impedance.py: A Python package for electrochemical impedance analysis. *J. Open Source Softw.* **5**, 2349 (2020).
33. Stringer, C. & Pachitariu, M. Cellpose3: one-click image restoration for improved cellular segmentation. *bioRxiv* 2024.02.10.579780 (2024).
34. Oswald, S., Pritzl, D., Wetjen, M. & Gasteiger, H. A. Novel Method for Monitoring the Electrochemical Capacitance by In Situ Impedance Spectroscopy as Indicator for Particle Cracking of Nickel-Rich NCMs: Part I. Theory and Validation. *J. Electrochem. Soc.* **167**, 100511 (2020).
35. Peters, J. F., Muthuswamy, M., Wibowo, J. & Tordesillas, A. Characterization of force chains in granular material. *Phys. Rev. E - Stat. Nonlinear, Soft Matter Phys.* **72**, 1–8 (2005).
36. Zhang, L. *et al.* The role of force chains in granular materials: from statics to dynamics. *Eur. J. Environ. Civ. Eng.* **21**, 874–895 (2017).
37. Lundkvist, A., Larsson, P. L. & Olsson, E. A discrete element analysis of the mechanical behaviour of a lithium-ion battery electrode active layer. *Powder Technol.* **425**, 118574 (2023).
38. Agnolin, I. & Roux, J. N. Internal states of model isotropic granular packings. I. Assembling process, geometry, and contact networks. *Phys. Rev. E - Stat. Nonlinear, Soft Matter Phys.* **76**, 1–27 (2007).
39. Roux, J. N. Geometric origin of mechanical properties of granular materials. *Phys. Rev. E - Stat. Physics, Plasmas, Fluids, Relat. Interdiscip. Top.* **61**, 6802–6836 (2000).
40. Bagi, K. Stress and strain in granular assemblies. *Mech. Mater.* **22**, 165–177 (1996).
41. Mäkinen, T. *et al.* Crack Propagation by Activated Avalanches during Creep and Fatigue from Elastic Interface Theory. *Phys. Rev. Lett.* **134**, 98202 (2025).
42. Bradley, W., Cantwell, W. J. & Kausch, H. H. Viscoelastic Creep Crack Growth: A Review of Fracture Mechanical Analyses. *Mech. Time-Dependent Mater.* **1**, 241–268 (1997).
